# Mechanistic Study of Alkyne Insertion into Cu–Al and Au–Al Bonds: A Paradigm Shift for Coinage Metal Chemistry

**DOI:** 10.1021/acs.inorgchem.2c03713

**Published:** 2022-12-09

**Authors:** Diego Sorbelli, Leonardo Belpassi, Paola Belanzoni

**Affiliations:** † Department of Chemistry, Biology and Biotechnologies, University of Perugia, Via Elce di Sotto, 8-06123 Perugia, Italy; ‡ CNR Institute of Chemical Science and Technologies ″Giulio Natta″ (CNR-SCITEC), Via Elce di Sotto, 8-06123 Perugia, Italy

## Abstract

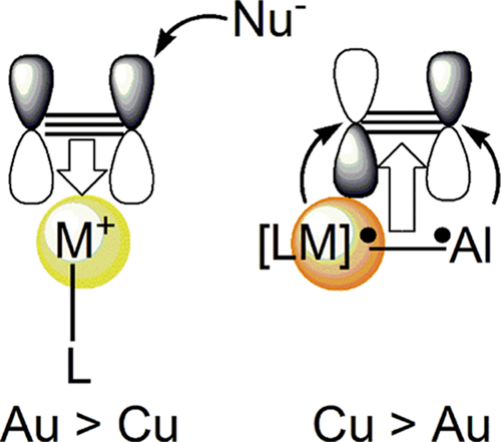

In this work, the mechanism of the insertion reaction of 3-hexyne into Cu–Al and Au–Al bonds in M–aluminyl (M = Cu, Au) complexes is computationally elucidated. The mechanism is found to be radical-like, with the Cu–Al and Au–Al bonds acting as nucleophiles toward the alkyne, and predicts a less efficient reactivity for the gold–aluminyl complex. The proposed mechanism well rationalizes the kinetic (or thermodynamic) control on the formation of the syn (or anti) insertion product into the Cu–Al bond (i.e., dimetallated alkene) which has been recently reported. A comparative analysis of the electronic structure reveals that the reduced reactivity at the gold site—usually showing higher efficiency than copper as a “standard” electrophile in alkyne activation—arises from a common feature, i.e., the highly stable 6s Au orbital. The relativistic lowering of the 6s orbital, making it more suitable for accepting electron density and thus enhancing the electrophilicity of gold complexes, in the gold–aluminyl system is responsible for a less nucleophilic Au–Al bond and, consequently, a less efficient alkyne insertion. These findings demonstrate that the unconventional electronic structure and the electron-sharing nature of the M–Al bond induce a paradigm shift in the properties of coinage metal complexes. In particular, the peculiar radical-like reactivity, previously shown also with carbon dioxide, suggests that these complexes might efficiently insert/activate other small molecules, opening new and unexplored paths for their reactivity.

## Abstract 2

The electron-sharing Au−Al and Cu−Al bonds in coinage metal−aluminyl complexes act as nucleophilic sites for the alkyne insertion, leading to a radical-like metal−aluminyl reactivity. The generally accepted factor determining superior “standard” electrophilic reactivity of gold complexes of [LAu]^+^ type in activating alkynes toward nucleophilic addition (i.e., the relativistic contracted and stabilized empty 6s orbital) is found here to be responsible for a less nucleophilic Au−Al bond (where the valence Au configuration is close to 6s^1^), which enables an Au > Cu to a Cu > Au shift for coinage metal reactivity.

## Introduction

**Scheme 1:**
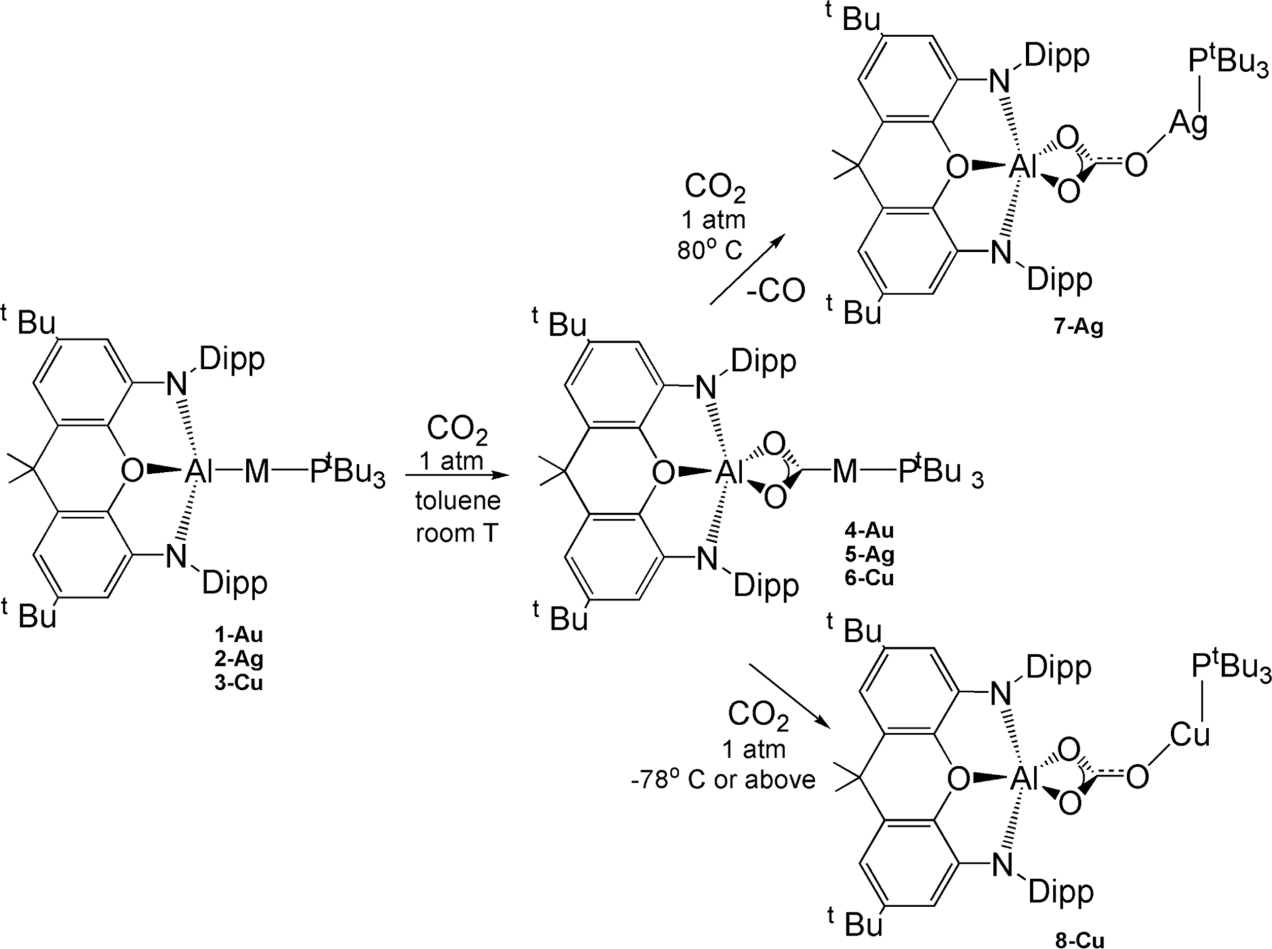
Reactions of Gold–Aluminyl **1-Au**, Silver–Aluminyl **2-Ag**, and Copper–Aluminyl **3-Cu** Complexes with CO_2_ Leading to Corresponding **4-Au**, **5-Ag**, and **6-Cu** Insertion Products

Recently, the CO_2_ insertion into M–Al bonds (M = Cu, Ag, Au) of coinage metal–aluminyl complexes has been reported.^1−4^ Depending on the group 11 metal nature, the product of carbon dioxide insertion, featuring the CO_2_ carbon atom coordinated to M and the two oxygen atoms bonded to the aluminyl moiety (**4-Au**, **5-Ag**, **6-Cu**), is stable to further reaction for the [*^t^*Bu_3_PAuAl(NON)] (NON = 4,5-bis(2,6-diisopropylanilido)-2,7-di-tert-butyl-9,9-dimethylxanthene) complex **1-Au**, whereas for the silver analogue [*^t^*Bu_3_PAgAl(NON)] complex **2-Ag**, the insertion product leads to the corresponding carbonate complex **7-Ag** (and CO), and for the copper system [*^t^*Bu_3_PCuAl(NON)] **3-Cu**, it proceeds rapidly to the carbonate **8-Cu** even at low temperatures (see Scheme 1).

For complex **1-Au**, we have demonstrated that the nucleophilic behavior of the electron-sharing, weakly polarized Au–Al bond combined with Al acting as an electrophile is the driving force for the CO_2_ insertion reactivity occurring via a cooperative radical-like mechanism.^5−8^ The main interaction in the CO_2_ insertion process has been shown to be electron donation from the Au–Al σ bond toward the CO_2_ LUMO, assisted by a secondary interaction where electron donation occurs from the CO_2_ HOMO toward the Al center (mostly an empty 3p_z_ orbital). These unique Au–Al electron-sharing bond nature and radical-like mechanism features, which are uncommon in gold(I) chemistry, make these species suitable candidates for insertion chemistry. In the same context, we have also analyzed the Cu–Al bond in **3-Cu**, which has been found to have an analogous electron-sharing nature with, however, a slight Cu(δ^+^)–Al(δ^–^) polarization that may lead to subtle differences in their reactivity.^5^

**Scheme 2:**
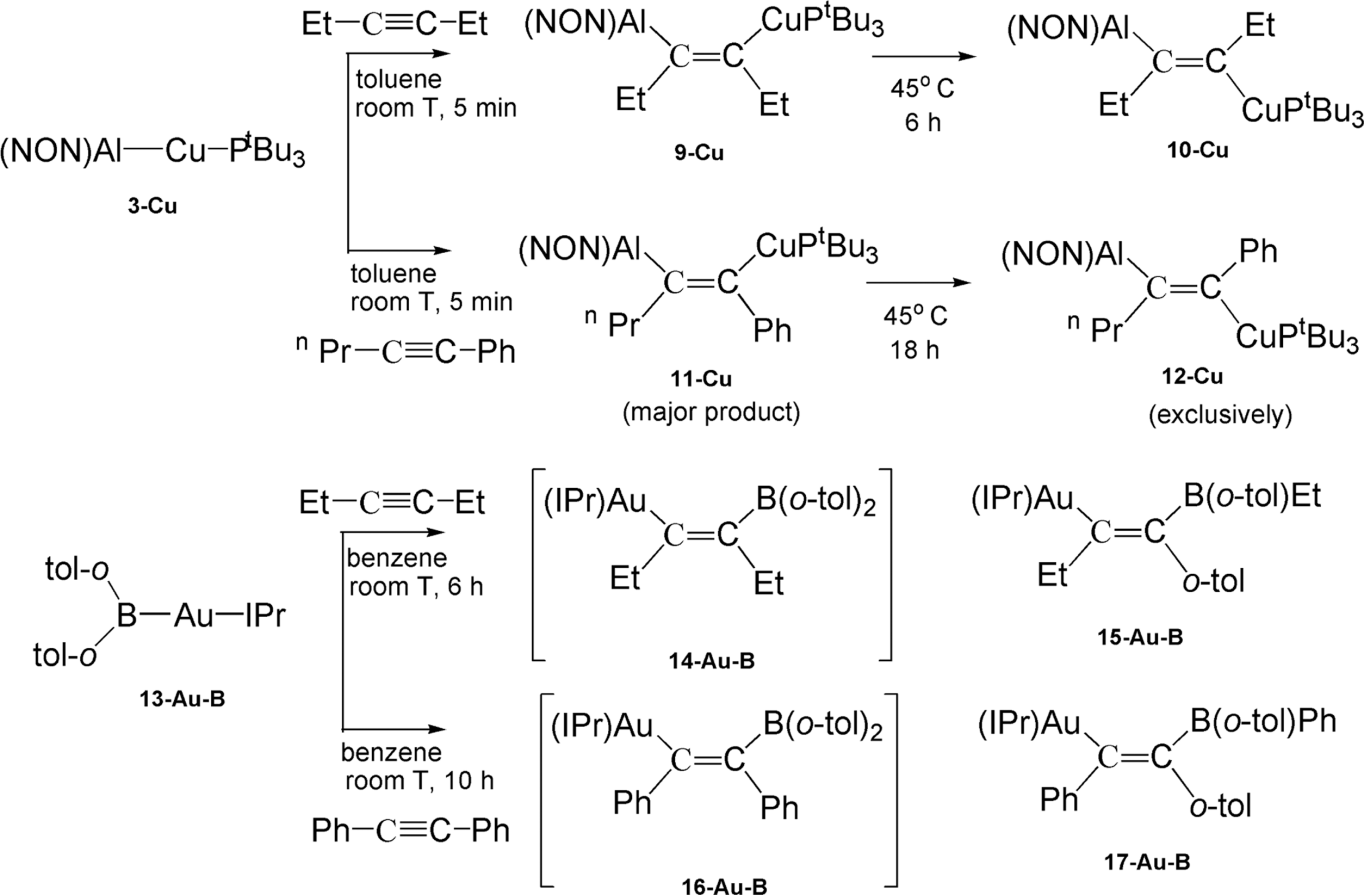
Reactions of Copper**–**Aluminyl **3-Cu** and Gold**–**Boryl **13-Au-B** Complexes with Internal Alkynes Leading to Corresponding Insertion Products (**9–12 Cu**) and Assumed Intermediates (**14-Au-B** and **16-Au-B**)

More recently, internal alkyne insertion into the Cu–Al bond of the copper–aluminyl complex **3-Cu** has been experimentally reported, producing (aluminylalkenyl)copper compounds which possess different reactivity at the two derived M–C functions.^9^ Complex **3-Cu** is able to control the nature of the formed (*syn* or *anti*) dimetallated alkenes, with *syn* isomers accessible through a kinetic control with high selectivity and *anti* isomers isolable through a thermodynamic control (Scheme 2). The *syn* dimetallated alkenes (**9-Cu**, **11-Cu**) can give a selective insertion of CO leading to the formation of copper acyl compounds.^9^

Strictly related gold silyl^10,11^ and boryl^12^ complexes have been also experimentally reported to insert alkynes into Au–Si and Au–B bonds. Amgoune and Bourissou reported that alkynes can be activated via insertion into the Au–Si bond of the gold silyl (R_3_P)AuSiR’Ph_2_ (R = Ph, Me; R’ = *^t^*Bu, Ph) systems with exclusively *syn* stereochemistry. Similarly, Yamashita et al. very recently reported an isomerization of a cis-(2-borylalkenyl)gold complex via a retro-1,2-metalate shift, where the first step is *syn* insertion of an internal alkyne into the Au–B bond of the [IPrAuB(Ar)_2_] (IPr = *N*,*N*’-bis(2,6-di-iso-propylphenyl)imidazole-2-ylidene; Ar = *o*-tolyl) gold boryl **13-Au-B** complex (Scheme 2).^12^

We have recently demonstrated that the Au–Al and Au–B bonds in [LAuX] (L = phosphine, *N*-heterocyclic carbene (NHC); X = Al(NON), B(*o*-tol)_2_) complexes possess a similar electron-sharing nature, with diarylboryl complexes displaying a slightly more polarized bond as Au(δ^+^)–B(δ^–^), which is responsible for a reduced radical-like reactivity toward CO_2_^8^ (consistent with the reaction of complex [IPrAuB(*o*-tol)_2_] with carbon dioxide having not been experimentally reported).^13^ The ancillary ligand of gold (phosphine or carbene) has been shown to have a negligible electronic trans effect on the Au–X bond and only a minor impact on the formation of the insertion product, although modification of the steric hindrance at the carbene site may exert a sizable control over the reaction.^8^

**Scheme 3:**
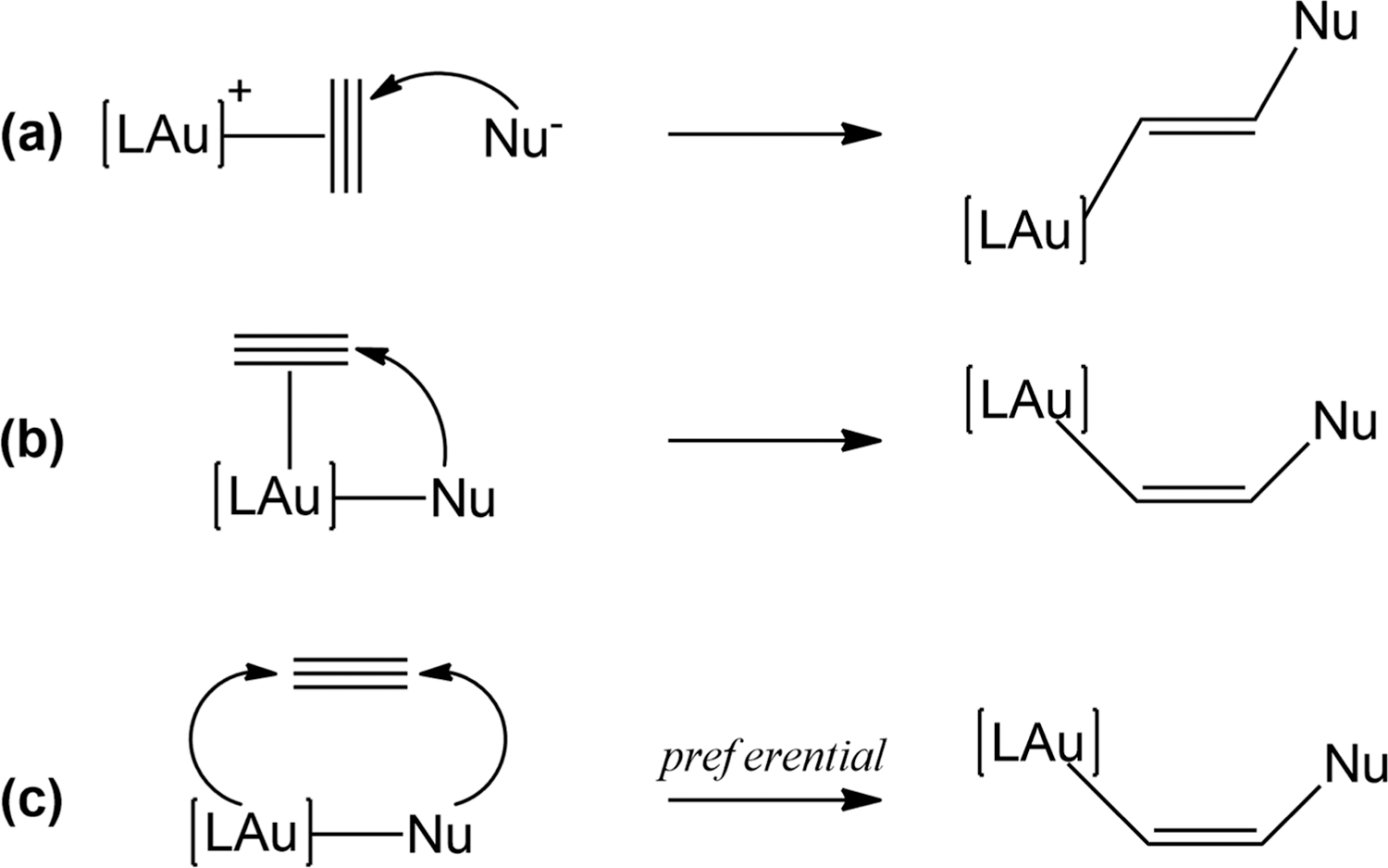
Schematic Representation of the (a) Outer-Sphere, (b) Inner-Sphere, and (c) Radical-like Mechanisms for the Nucleophilic Attack Step on the Alkyne Triple Bond

In this framework, one might surmise that the electron-sharing nature of the Au–Al bond in complex **1-Au** which allowed to rationalize its reactivity with CO_2_ may favor the reaction of complex **1-Au** with alkynes analogous to that of complex **3-Cu**. A comparative investigation of alkyne insertion in complexes **1-Au** and **3-Cu** would be relevant to shed light on the metal effect on this reactivity by a detailed comparison between Au–Al and Cu–Al bond nature and reaction mechanisms. As mentioned, a radical-like reactivity is new in gold chemistry, substantially differing from the well-known strong carbophilic Lewis acid behavior of gold complexes toward alkynes.^14^ Most of the reactions in which gold catalysts are involved can be classified as nucleophilic additions to a carbon–carbon unsaturated bond (alkynes, alkenes, and allenes), proceeding in a stepwise manner. In typical gold complexes of LAuX type (L = phosphine, carbene; X = counterion), the facile substitution of X with the alkyne substrate (pre-equilibrium step) allows for a η^2^ coordination of the CC triple bond to the Au center which, acting as an electrophile, activates it for the subsequent nucleophilic attack by Nu–H (for instance, alcohols, water, amines, etc.), with the formation of vinyl gold intermediates and subsequent cleavage of the gold–carbon bonds by a proton (protodeauration step), to give the desired products and regenerate the catalyst.^15−19^ Remarkably, the nucleophilic attack commonly occurs on the Au–CC π complex anti to gold, affording an organogold complex with *trans* arrangement of the nucleophile and gold center (outer-sphere mechanism, Scheme 3), as supported by several experimental and computational studies.^15−27^

Alternatively, the nucleophilic attack has been proposed to involve coordination of the nucleophile to gold and *syn* insertion of the substrate into the Au–Nu bond, leading to *cis* vinyl gold intermediates (inner-sphere mechanism, Scheme 3). However, no direct unambiguous evidence for the inner-sphere mechanism has been reported to date.^28−32^ Cu-catalyzed reactions of internal alkynes for the synthesis of different valuable alkenes and heterocycles have also appeared in the literature in the last decade and they follow analogous mechanisms, with the functionalization occurring via both an inner- and an outer-sphere mechanism.^33^

However, the M–alkyne (M = Cu, Au) bond energies vary generally in the order Cu < Au, indicating a more efficient activation of the triple bond by gold complexes, due to their higher electrophilicity. In addition, dinuclear gold (and heterobimetallic Cu/Au) complexes and their use in catalysis have recently received significant attention and comparative studies of the controversial mono- vs dual-metal-catalyzed pathways have appeared in the literature.^34−39^

From a more general perspective, insertion reactions of organic molecules into heterobimetallic M–M’ or M–E (E = B, Si) bonds represent an attractive approach to synthetic organic chemistry and industrial catalysis, although examples of this heterobimetallic reactivity are still scarce.^10−12,40,41^ One challenge in heterobimetallic catalysis is identifying a metal cooperative effect (if any) and its origin through mechanistic investigation, which can provide the necessary insight to understand how these catalysts work and possibly how modifications can be planned to increase both the activity and selectivity.

In this work, we computationally elucidate the mechanism of the experimentally observed alkyne (3-hexyne) insertion reaction into the Cu–Al bond in complex **3-Cu** which is compared to that of the analogous model insertion reaction into the Au–Al bond in complex **1-Au**. Detailed mechanistic analysis highlights the superior ability of the copper–aluminyl complex in activating (and inserting) the alkyne, leading to a more efficient formation of the *syn* product. The superior activity of **3-Cu** with respect to **1-Au** is found to be consistent with the relativistic stabilization of the gold valence atomic orbitals, in particular the 6s orbital, which is commonly known to enhance the electrophilicity of gold complexes and which, in this case, is responsible for a less efficient nucleophilic behavior of the gold–aluminum electron-sharing bond, thus inducing a paradigm shift in the coinage metal chemistry.

## Results and Discussion

### Reaction Mechanism of the Alkyne Insertion into the Cu–Al Bond

**Figure 1:**
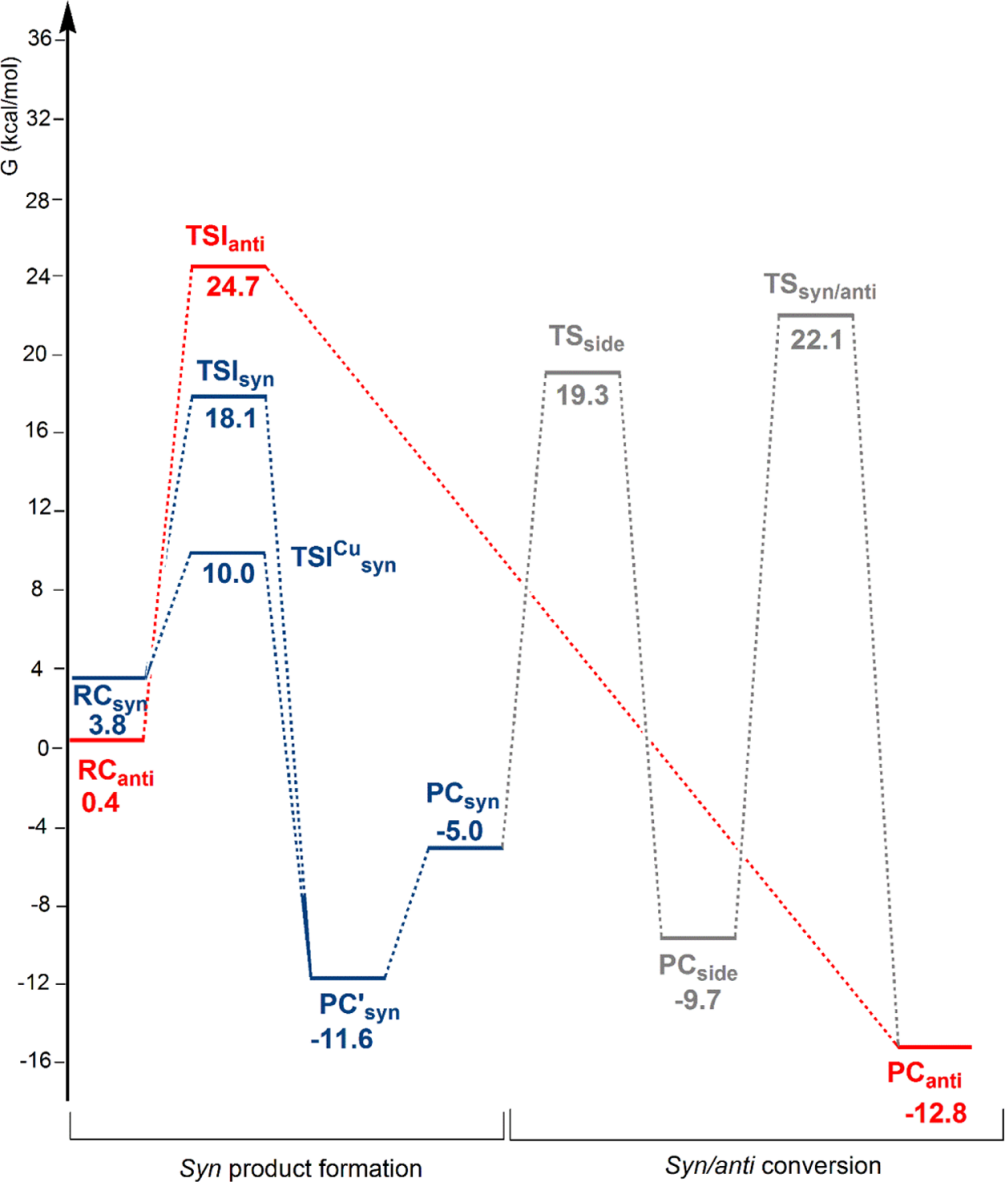
Free-energy reaction profile for the 3-hexyne insertion into the Cu–Al bond in [*^t^*Bu_3_PCuAl(NON)] (**3-Cu**) complex: the *anti* insertion path (red lines), the *syn* insertion path (blue lines), and the *syn*/*anti* conversion path (gray lines). Δ*G* values refer to the energy of the separated reactants taken as zero.

**Figure 2:**
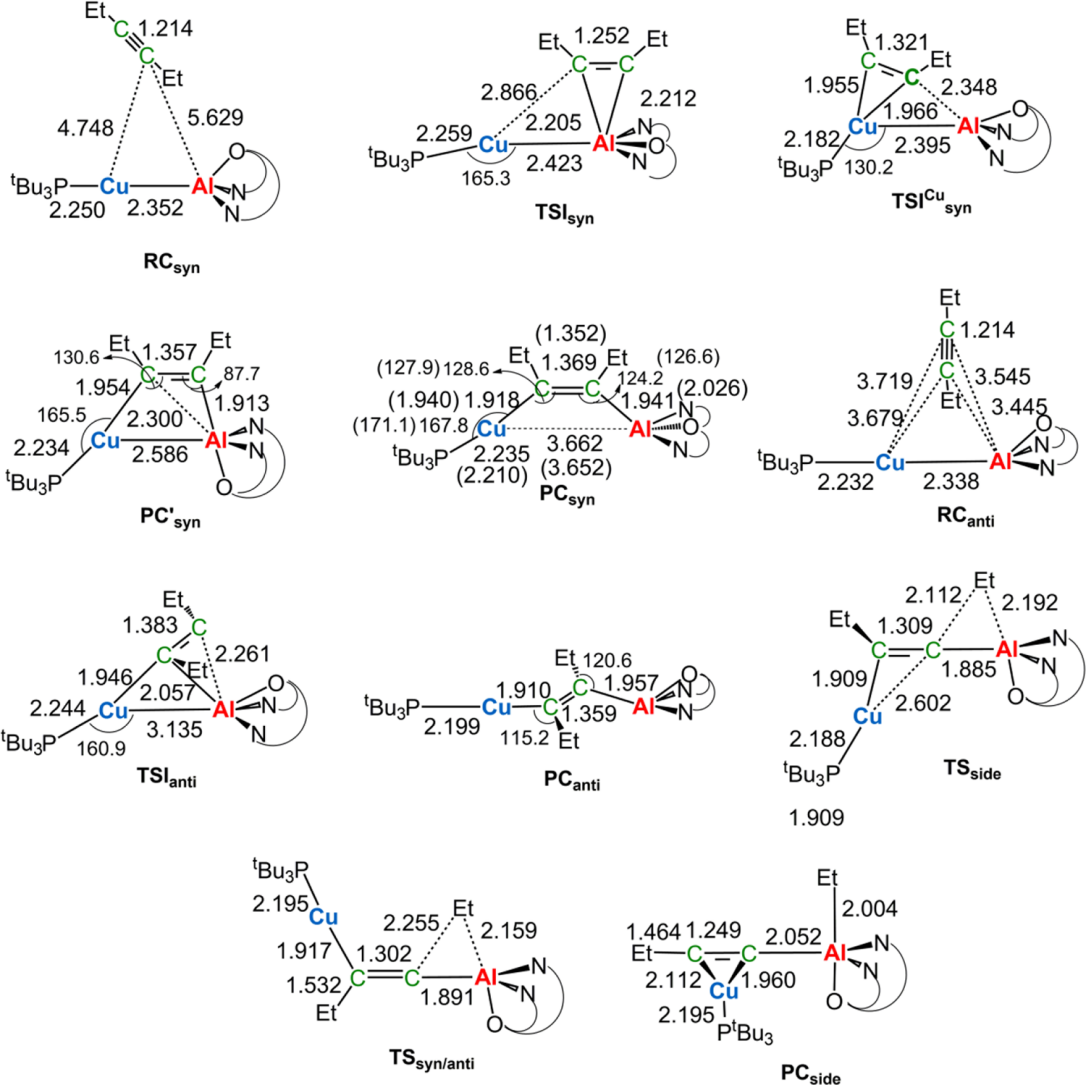
Sketched stationary point structures along the paths of Figure 1 with selected interatomic distances (Å) and bond angles (°). Values in parentheses refer to experimentally available data taken from ref (9).

The free-energy profile for 3-hexyne insertion into the Cu–Al bond of complex **3-Cu** is illustrated in Figure 1. It has been calculated using the same computational setup as that employed in ref (5) for the insertion reaction of CO_2_ into the Au–Al bond in complex **1-Au** (see the Computational Details Section). Optimized structures of stationary points along the paths are also sketched with selected geometrical parameters in Figure 2.

Starting from the reactant complex **RC**_**anti**_, where the substrate CC triple bond is oriented perpendicular to the Cu–Al bond, alkyne insertion occurs with an activation free-energy barrier Δ*G*^≠^ = 24.3 kcal/mol (via **TSI**_**anti**_) leading directly to the formation of the *anti* insertion product (**PC**_**anti**_) (red line profile). The process is exergonic with Δ*G* = −12.8 kcal/mol.

Reactant complex **RC**_**syn**_, featuring the CC triple bond parallel to the Cu–Al bond, allows for alkyne insertion (blue line profiles) occurring both at the Al (via **TSI**_**syn**_, Δ*G*^≠^ = 14.3 kcal/mol) and at the Cu (via **TSI**_**syn**_^**Cu**^, Δ*G*^≠^ = 6.2 kcal/mol) sites and leading to a stable intermediate species **PC’**_**syn**_ (Δ*G* = −11.6 kcal/mol) which, upon Cu–Al bond stretching, yields a less stable *syn* insertion product (**PC**_**syn**_, Δ*G* = −5.0 kcal/mol). This reaction mechanism is in good agreement with the experimental finding that formation of the *syn* dimetallated alkenes is under kinetic control (lower activation barriers are calculated for *syn* insertion). Notably, in the **TSI**_**syn**_ and **TSI**_**syn**_^**Cu**^ structures (Figure 2), both the Cu and Al sites are involved in a concerted mechanism, preferentially starting at the Cu site. As for the thermodynamic control for the formation of the *anti* isomer, comparison of the **PC**_**syn**_ main geometrical parameters with available X-ray crystallography data (see Figure 2) shows that this species is in good agreement with experimentally reported parameters, thus representing the isolated species, although **PC**_**syn**_ is found to be less stable than **PC**_**anti**_ by 7.8 kcal/mol (and **PC’**_**syn**_ by 6.6 kcal/mol). The interconversion of **PC**_**syn**_ to **PC**_**anti**_ has been proposed to occur through a C-to-Al migration of an Et group based on the isolated and structurally characterized (NON)AlEt species^9^ and in analogy with the reactivity of the boryl gold compound reported by Yamashita et al.^12^ Access to *anti* dimetallated alkene has been investigated through a two-step process via **TS**_**side**_ and **TS**_**syn/anti**_ transition states (gray line, Figure 1), i.e., thorough isomerization of the **PC**_**syn**_ species. The calculated barrier for the first step (denoted as “side reaction”) leading to (NON)AlEt and copper acetylide co-product **PC**_**side**_ is Δ*G*^≠^ = 24.3 kcal/mol, indicating that the migration of the Et group is viable from **PC**_**syn**_ along the path for the *syn*/*anti* conversion. Formation of **PC**_**side**_ is exergonic by 4.7 kcal/mol and dissociation into (NON)AlEt and [*^t^*Bu_3_PCuCCEt] separated species requires only 2.0 kcal/mol (see Figure S1). The **PC**_**side**_ conversion to **PC**_**anti**_ involves the Et group migration to the alkyne (via **TS**_**syn/anti**_) with a high activation energy Δ*G*^≠^ = 31.8 kcal/mol. These results indicate that the rate-determining step is the isomerization from **PC**_**side**_ to **PC**_**anti**_, which is consistent with the more forcing experimental conditions (heating to 45 °C for 6 h) needed to access the *anti* insertion product and with the experimental observation that (NON)AlEt can be isolated from the reaction mixture, whereas assignment of *anti* dimetallated alkene (**PC**_**anti**_) could not be confirmed crystallographically for 3-hexyne.

The preferential formation of **PC**_**syn**_ via **TSI**_**syn**_^**Cu**^ with respect to the other pathways described above can be rationalized by analyzing the evolution of the electronic structure along the reaction path. In particular, the analysis of the alkyne–complex interaction at the three initial transition states for each path (i.e., **TSI**_**syn**_, **TSI**_**syn**_^**Cu**^, and **TSI**_**anti**_) using energy decomposition analysis (EDA),^42^ natural orbitals for chemical valence (NOCV),^43^ and charge-displacement (CD)^44−46^ approaches, in combination with the activation strain model^47−49^ (ASM, see the Methodology section in the SI for details on these approaches), allows to gain quantitative insights into the factors controlling the reported reactivity.

**Figure 3:**
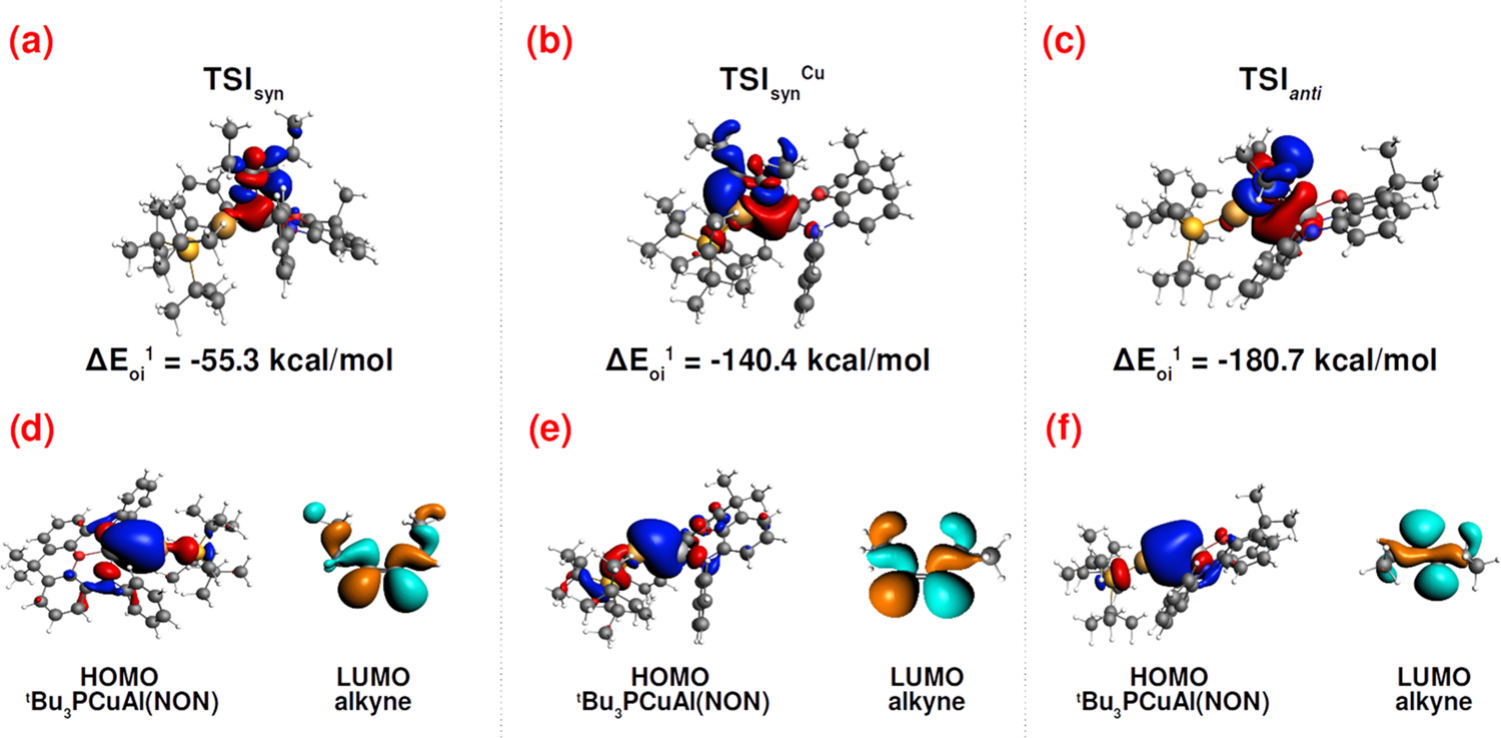
Main results of the NOCV analysis (Δρ_1_’ component) of the [EtCCEt]*−*[*^t^*Bu_3_PCuAl(NON)] interaction at **TSI**_**syn**_, **TSI**_**syn**_^**Cu**^, and **TSI**_**anti**_. The isosurfaces for the Δρ_1’_ component at **TSI**_**syn**_, **TSI**_**syn**_^**Cu**^, and **TSI**_**anti**_ (a, b, and c, respectively) are shown together with the associated orbital interaction energy and the corresponding main molecular orbitals contributions (d, e, and f for **TSI**_**syn**_, **TSI**_**syn**_^**Cu**^, and **TSI**_**anti**_, respectively). The charge flux is red → blue. Isovalue is 2 me/a_0_^3^ for (a*–*c), while it is 20 me/a_0_^3^ for (d–f). See Figures S2–S7 in the SI for the complete NOCV analysis.

As depicted in Figures 3 and S2–S7 and Table S1 in the SI, the alkyne–complex interaction at **TSI**_**syn**_, **TSI**_**syn**_^**Cu**^, and **TSI**_**anti**_ for the copper–aluminyl complex is, on a qualitative ground, of analogous nature.

Despite a different geometrical rearrangement of both the complex and alkyne moieties, the NOCV analysis reveals a qualitatively analogous interaction at the three TSs, where the main component Δρ_1_’ identifies a charge transfer arising from the electron-sharing Cu–Al bond (as it can be inferred by the large contribution of the σ Cu–Al centered HOMO of the complex in all cases) toward the π* LUMO of the alkyne fragment (Figures S2, S4, and S6). A second significant component of the complex–alkyne interaction for all TSs can be observed (Δρ_2_’), which is also qualitatively analogous in all cases and features a reverse alkyne-to-complex charge transfer toward an unoccupied molecular orbital of the complex with large contributions from the empty Al 3p_z_ atomic orbital (Figures S3, S5, and S7). It is worth noting that the alkyne–complex interaction scheme discussed here is highly reminiscent of that previously reported for the [*^t^*Bu_3_AuAl(NON)]–CO_2_ interaction,^5−8^ indicating a general scheme for the interaction of this class of complexes with small molecules.

**Figure 4:**
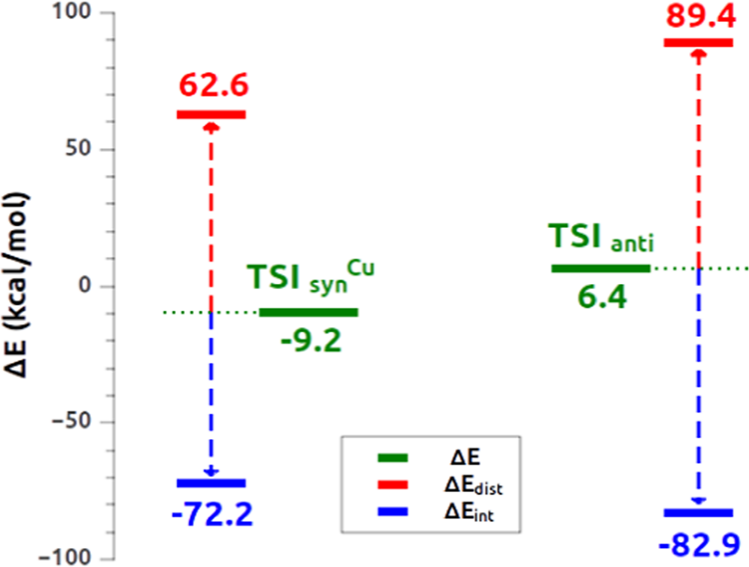
Activation strain model (ASM) decomposition of the relative energy (Δ*E*) of **TSI**_**syn**_^**Cu**^ (left) and **TSI**_**anti**_ (right) into distortion (Δ*E*_dist_) and interaction (Δ*E*_int_) contributions.

Despite these evident qualitative analogies, on a quantitative ground, the ASM approach (Table S2 in the SI and Figure 4) helps to rationalize the relevant differences between the disfavored *anti* pathway via **TSI**_**anti**_ and the *syn* pathway, particularly the preferential reactivity via **TSI**_**syn**_^**Cu**^.

Although the interaction energy between the alkyne and the complex appears to favor **TSI**_**anti**_ with respect to **TSI**_**syn**_^**Cu**^ (Δ*E*_int_ is −82.9 and −72.2 kcal/mol, respectively, see Figure 4), thus apparently suggesting a more stable **TSI**_**anti**_, the ASM results indicate that the balance between these favorable interactions and the disfavoring distortion penalty is key. As shown in Figure 4, the distortion penalty is much larger in the case of **TSI**_**anti**_ (89.4 kcal/mol), while it is lower by almost 30 kcal/mol in the case of **TSI**_**syn**_^**Cu**^ (62.6 kcal/mol), leading to **TSI**_**syn**_^**Cu**^ being more stable than **TSI**_**anti**_ in terms of relative electronic energy (−9.2 vs 6.4 kcal/mol, respectively), in agreement with the lower free-energy barrier associated with the *syn* path (see Figure 1) which determines the kinetic control discussed earlier.

The reason behind the reduced distortion destabilization at **TSI**_**syn**_^**Cu**^ can be found by inspection of the geometry of the two transition states in Figure 2. While at **TSI**_**syn**_^**Cu**^ the *syn* approach of the alkyne leads to a transition state in which the Cu–Al bond is unbroken (the Cu–Al bond length is 2.385 Å), at **TSI**_**anti**_ the copper–aluminum bond is cleaved (the Cu–Al bond length is 3.135 Å). Since the Cu–Al bond is a fairly strong bond (the calculated dissociation energy is −78.4 kcal/mol), this leads to a distortion penalty associated with the deformation of the complex which is clearly higher for **TSI**_**anti**_ (26.6 kcal/mol, Table S2) with respect to that for **TSI**_**syn**_^**Cu**^ (14.9 kcal/mol, Table S2). This difference, combined with the higher energy penalty at the alkyne site (Δ*E*_dist_^alkyne^ is 47.7 and 62.7 kcal/mol for **TSI**_**syn**_^**Cu**^ and **TSI**_**anti**_, respectively) due to the higher degree of alkyne deformation (the C–C bond length is 1.321 vs 1.383 Å for **TSI**_**syn**_^**Cu**^ and **TSI**_**anti**_, respectively), explains the preferential *syn* formation from a kinetic standpoint.

Finally, it is worth exploring the nature of the reactivity in relation to the formed products, especially considering that gold–aluminyl complexes have been reported to react with CO_2_ with a radical-like reactivity, i.e., with gold and aluminyl fragments behaving as doublet species when capturing CO_2_.^5−8^ Based on the scheme previously used for gold–aluminyl complexes,^5−8^ we describe the formation of **PC**_**syn**_, **PC**_**syn**_**’**, and **PC**_**anti**_ from both radical doublet [*^t^*Bu_3_PCu]· and [Al(NON)]· and closed shell charged [*^t^*Bu_3_PCu]^+^ and [Al(NON)]^−^ fragments (see Scheme S1 in the SI). The results, reported in Table S3 in the SI, strongly suggest that a similar radical-like reactivity is also observed for copper–aluminyl reacting with the alkyne. Indeed, the overall formation energy of the products (Δ*E*) is found to be lower starting from radical fragments (−102.2, −106.9, and −106.9 kcal/mol for **PC**_**syn**_, **PC**_**syn**_**’**, and **PC**_**anti**_, respectively) with respect to that from the corresponding closed shell fragments (−131.6, −136.3, and −136.3 kcal/mol for **PC**_**syn**_, **PC**_**syn**_**’**, and **PC**_**anti**_, respectively), thus indicating the higher stability of the radical fragments, in strict analogy with the reaction of **1-Au** with CO_2_. This finding further elucidates that (i) the alkyne insertion occurs neither with an outer- nor an inner-sphere but more properly via a radical-like mechanism relying on a nucleophilic Cu–Al bond as the driving force and (ii) the reactivity mode previously found for gold–aluminyl complexes with CO_2_ (i.e., a cooperative radical-like reactivity) emerges here as a more general paradigm for coinage metal–aluminyl complexes (and electron-sharing M–Al bonds) efficiently reacting with small molecules.

### Reaction Mechanism of the Alkyne Insertion into the Au–Al Bond

**Figure 5:**
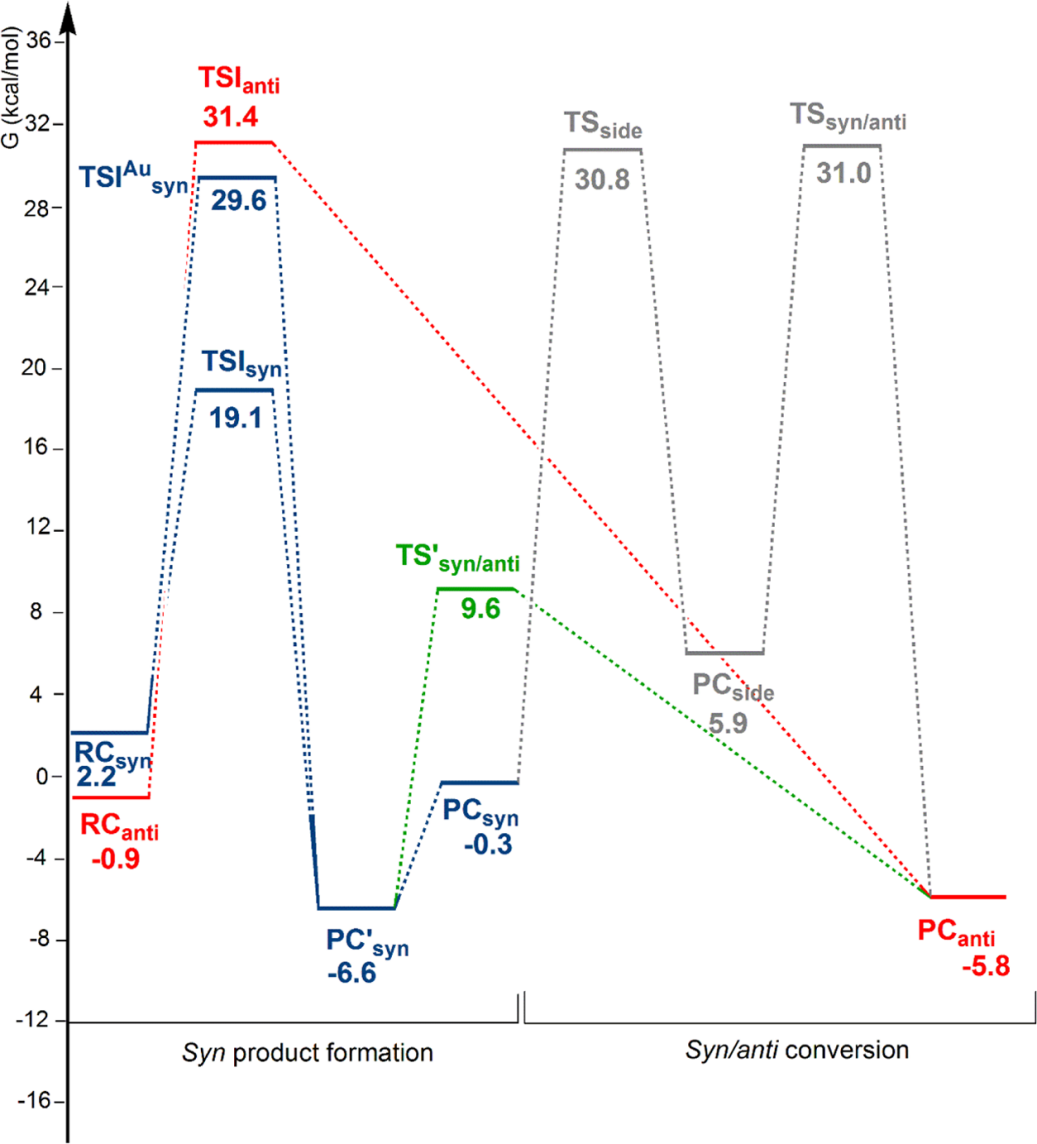
Free-energy reaction profile for the 3-hexyne insertion into the Au–Al bond in [*^t^*Bu_3_PAuAl(NON)] (**1-Au**) complex: the *anti* insertion path (red lines), the *syn* insertion path (blue lines), and the *syn*/*anti* conversion paths (direct path: green lines; two-step path: gray lines). Δ*G* values refer to the energy of the separated reactants taken as zero.

**Figure 6:**
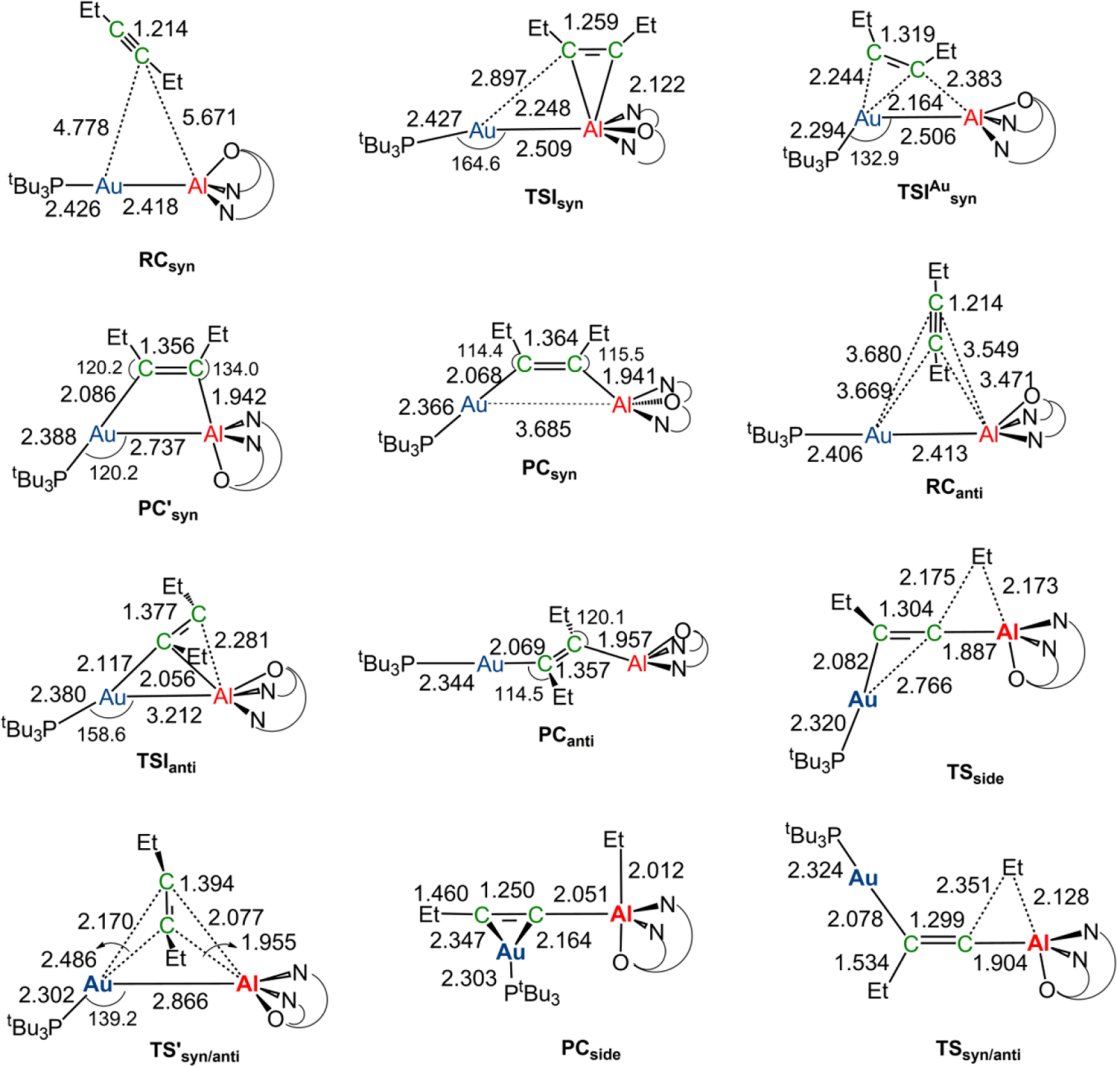
Sketched stationary point structures along the paths in Figure 5 with selected interatomic distances (Å) and bond angles (°).

The free-energy profile for 3-hexyne insertion into the Au–Al bond of complex **1-Au** is illustrated in Figure 5, with the optimized structures of stationary points along the paths sketched with selected geometrical parameters in Figure 6.

The reaction paths are qualitatively similar to those for **3-Cu**, with some relevant quantitative differences. Alkyne *anti* insertion occurs with an activation free-energy barrier of 32.3 kcal/mol (via **TSI**_**anti**_) leading directly to the formation of the *anti* insertion product (**PC**_**anti**_) (red line profile, Δ*G* = −5.8 kcal/mol). Analogously, the *syn* insertion via **TSI**_**syn**_ (Δ*G*^≠^ = 16.9 kcal/mol) or via **TSI**_**syn**_^**Au**^ (Δ*G*^≠^ = 27.4 kcal/mol) leads to a stable intermediate species **PC’**_**syn**_ (Δ*G* = −6.6 kcal/mol) which, upon Au–Al bond stretching, yields a less stable *syn* insertion product (**PC**_**syn**_, Δ*G* = −0.3 kcal/mol). As a result, formation of the *syn* dimetallated alkenes occurring at the Au–Al site is similarly predicted under kinetic control, with a higher activation barrier compared to that calculated for **3-Cu** (16.9 vs 6.2 kcal/mol). Similarly, in the **TSI**_**syn**_ and **TSI**_**syn**_^**Au**^ structures (Figure 6), both the Au and Al sites are involved, reflecting again a cooperative effect of the two centers in the alkyne insertion, with a more relevant role of Al. Notably, in the **RC**_**syn**_ structure, the Au–Al bond distance (2.418 Å) is close to the experimental value reported for complex **1-Au** (2.402 Å)^1^ and slightly shorter than the sum of the single-bond covalent radii of Au and Al (2.50 Å).^50^ Preferential formation of the *syn* product via kinetic control can be explained on the basis of what was discussed in the previous section for **3-Cu**, i.e., the enhanced distortion penalty at **TSI**_**anti**_ due to a substantially broken Au–Al bond (Au–Al bond length is 3.212 Å, see Figure 6) leading to a higher activation barrier (see Table S5 in the SI for the ASM results).

Analogously to **3-Cu**, **PC**_**syn**_ has a lower stability than **PC**_**anti**_ (by 5.5 kcal/mol) and **PC’**_**syn**_ (by 6.3 kcal/mol), and, remarkably, the **RC**_**syn**_**-to-PC**_**syn**_ conversion is practically thermoneutral, thus suggesting a possibly reversible reaction with a **PC**_**syn**_ that experimentally may be hardly isolable.

Interestingly, the formation of the *syn* products is predicted to occur via different paths for **3-Cu** and **1-Au**. Whereas the former is predicted to form more favorably the corresponding **PC’**_**syn**_ via **TSI**_**syn**_^**Cu**^ (i.e., via a transition state where the alkyne is coordinated to the metal in a roughly η^2^ fashion) with an activation barrier of 6.2 kcal/mol, the kinetically favored path for **1-Au** is predicted to be via **TSI**_**syn**_, which features a lower activation barrier (Δ*G*^≠^ = 16.9 kcal/mol) with respect to that via **TSI**_**syn**_^**Au**^ (Δ*G*^≠^ = 27.4 kcal/mol), thus suggesting that the alkyne η^2^-like coordination to Au leads to a less stable transition state.

EDA, CD, NOCV, and ASM approaches help to shed light on these differences (see Tables S4, S5, and Figures S8–S13 in the SI for the complete results). On a qualitative ground, the alkyne–complex interaction appears to be unaffected by replacing the copper metal center with gold, coherently with the very similar reported M–Al bond features. The main NOCV component of the interaction of the substrate with **1-Au** at all TSs (Δρ_1_’) consists of a charge transfer from the Au–Al σ-bonding orbital toward the LUMO of the alkyne (see Figures S8, S10, and S12 in the SI). In all cases, a second component (Δρ_2_’) is observed which consists of a charge transfer from the alkyne HOMO toward the LUMO of the complex (see Figures S9, S11, and S13 in the SI).

On a quantitative ground, however, the differences concerning the stability of **TSI**_**syn**_^**Cu**^ and **TSI**_**syn**_^**Au**^, which determine the different preferential paths for **3-Cu** and **1-Au**, respectively, become evident. In particular, comparison of ASM results (see Tables 1, S2, and S5 in the SI) clarifies that the interaction between the substrate and the complex contribution (Δ*E*_int_) is found to be the determining factor driving their different stability.

**Table 1:** Most Relevant ASM (Top) and EDA-NOCV (Bottom) Results for **TSI**_**syn**_^**Cu**^ and **TSI**_**syn**_^**Au**^^1^

	TSI_syn_^Cu^	TSI_syn_^Au^
ASM decomposition of Δ*E*		
Δ*E*	–9.6	14.2
Δ*E*_dist_	62.6	64.0
Δ*E*_int_	–72.2	–49.8
EDA decomposition of Δ*E*_int_		
Δ*E*^Pauli^	346.1	357.0
Δ*E*_elst_	–217.7	–218.5
Δ*E*_steric_	128.4	138.5
Δ*E*_oi_	–187.6	–176.0
Δ*E*_oi_^1^	–140.4	–127.2
Δ*E*_oi_^2^	–20.1	–22.9
Δ*E*_disp_	–13.0	–12.3

1 See Tables S2 and S5 in the SI for detailed ASM results and Tables S1 and S4 in the SI for detailed EDA-NOCV and CD results. All energies are reported in kcal/mol.

As shown in Table 1, while both **TSI**_**syn**_^**Cu**^ and **TSI**_**syn**_^**Au**^ feature analogous distortion penalties (62.6 and 64.0 kcal/mol, respectively), the stabilizing interaction contribution is different (−72.2 vs −49.8 kcal/mol, respectively), favoring an overall more stable **TSI**_**syn**_^**Cu**^ (Δ*E* is −9.6 and 14.2 kcal/mol, respectively). The EDA-NOCV approach allows to decompose the interaction energy term Δ*E*_int_, showing in detail that the complex–alkyne interaction mainly differs at the two TSs due to an orbital interaction increase (Δ*E*_oi_ is −187.6 vs −176.0 kcal/mol for **TSI**_**syn**_^**Cu**^ and **TSI**_**syn**_^**Au**^, respectively) and, in particular, to a more energetically stabilizing complex-to-alkyne charge transfer (Δ*E*_oi_^1^ is −140.4 vs −127.2 kcal/mol for **TSI**_**syn**_^**Cu**^ and **TSI**_**syn**_^**Au**^, respectively). The Pauli repulsion component also differs in the two TSs, being significantly lowered at **TSI**_**syn**_^**Cu**^ (Δ*E*_Pauli_ is 346.1 vs 357.0 kcal/mol for **TSI**_**syn**_^**Cu**^ and **TSI**_**syn**_^**Au**^, respectively).

**Figure 7:**
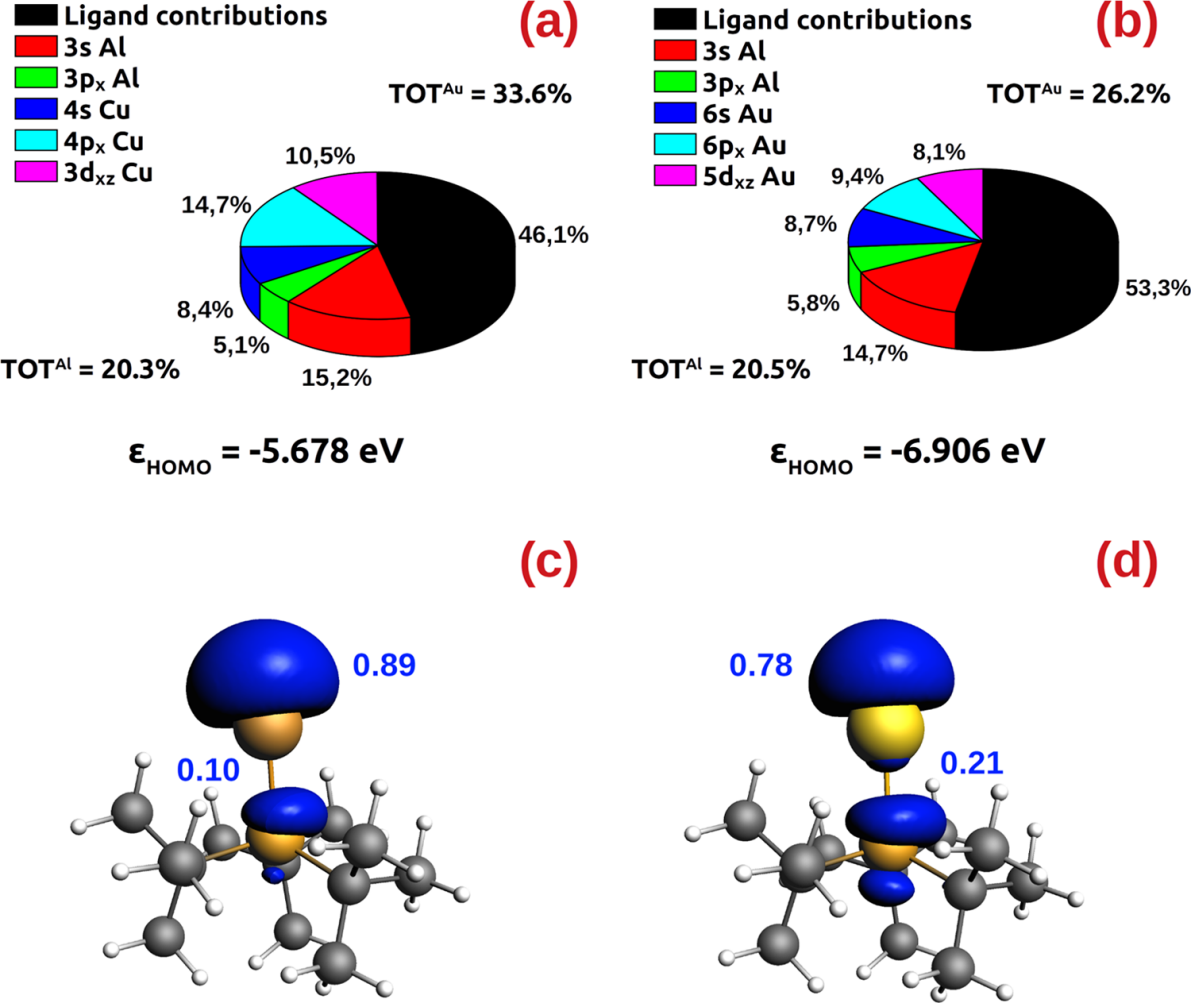
Atomic orbital contributions to the HOMO of **3-Cu** (a) and **1-Au** (b) at the **TSI**_**syn**_^**Cu**^ and **TSI**_**syn**_^**Au**^ structures, respectively, and HOMO energy. Spin density distribution in the radical [*^t^*Bu_3_M]· (M = Cu, Au) fragments at the **TSI**_**syn**_^**Cu**^ and **TSI**_**syn**_^**Au**^ geometry (c, d, respectively) (isodensity value 3 me/a_0_^3^) and Mulliken population on M and P.

The larger Pauli repulsion contribution at **TSI**_**syn**_^**Au**^ may be rationalized on the basis of the more spatially extended 5d Au compared to the 3d Cu orbitals, as recently highlighted by Toste, Head-Gordon and co-workers.^51^ On the other hand, the increased orbital energy stabilization coming from the increased Δ*E*_oi_ (and in particular Δ*E*_oi_^1^) at **TSI**_**syn**_^**Cu**^ with respect to **TSI**_**syn**_^**Au**^ can be explained by analyzing the HOMO (i.e., the main filled molecular orbital of the complexes involved in the Δρ_1_’ component) of the metal–aluminyl complexes at their corresponding transition state structure, as shown in Figure 7.

From Figure 7a, the composition of the **3-Cu** HOMO at the **TSI**_**syn**_^**Cu**^ structure features contributions from copper 4s, 4p*_x_*, and 3d*_xz_* atomic orbitals which sum up to 33.6% (8.4, 14.7, and 10.5% contributions from Cu 4s, 4p*_x_*, and 3d*_xz_* orbitals, respectively). Coupled with contributions from Al (20.3%), overall the **3-Cu** HOMO at **TSI**_**syn**_^**Cu**^ is mostly centered on the two metals (53.9%), quantitatively rationalizing the cooperative behavior of the two metals in the reported reactivity. Concerning **1-Au** (Figure 7b), while the Al orbitals give analogous contributions (20.5%), the **1-Au** HOMO at **TSI**_**syn**_^**Au**^ has less coinage metal character (overall 26.2%, with 8.7, 9.4, and 8.1% contributions from Au 6s, 6p*_x_*, and 5d*_xz_* atomic orbitals, respectively). This different composition leads to an overall lower bimetallic character of the HOMO (46.7%). As a result, the reactivity of **1-Au** via **TSI**_**syn**_^**Au**^ is less efficient and this can be quantitatively inferred also by the energy of the HOMO, which is more stable (and thus, according to the frontier molecular orbital theory, less reactive) for **1-Au** (−3.922 eV) than for **3-Cu** (−3.787 eV, see Figure 7a,b).

These results can be rationalized on the basis of the radical-like reactivity of the metal (and aluminyl) fragments and metal’s atomic orbitals. As shown in Scheme S1 and Tables S3 and S6 in the SI, the product formation is favored by starting from [*^t^*Bu_3_PM]· (M = Cu, Au) and [Al(NON)]^·^ radical fragments, suggesting a radical-like reactivity of both gold– and copper–aluminyl complexes, also consistent with gold’s valence 5d^10^6s^1^ configuration in gold–aluminyl complexes we recently discussed.^52^ Upon spin density analysis on the two isolated metal fragments at the geometry they have in **TSI**_**syn**_^**Cu**^ and **TSI**_**syn**_^**Au**^ (Figure 7c,d), clear differences emerge between gold and copper. The unpaired electron in the radical [*^t^*Bu_3_PCu]^·^ fragment is highly localized on Cu (0.89 e) with only a small delocalization on P (0.10 e). Conversely, the unpaired electron on [*^t^*Bu_3_PAu]^·^ is less localized on Au (0.78 e) and more delocalized on P (0.21 e). As a result, the unpaired electron on Au is less available for the reactivity with 3-hexyne at the nucleophilic site, leading to a reduced bimetallic character and to a lower-energy and less reactive HOMO with respect to Cu. This decreased availability of the 6s^1^ electron on gold is consistent with the relativistic stabilized 6s orbital in Au (−5.678 eV), which is remarkably close in energy to the 3p_x_ atomic orbital of the ligand’s phosphorus (−5.506 eV), leading to an efficient overlap and delocalization of the unpaired electron. By contrast, the copper 4s orbital is at higher energy (−4.640 eV), which reduces the overlap with the P orbital and the delocalization toward the ligand, and, consequently, it makes the electron at the copper site more available for the reaction with 3-hexyne.

Interestingly, the picture we describe here is well known in the framework of coinage metal chemistry. It is widely accepted that many of the unique properties of gold complexes, such as the high electrophilicity and carbophilicity, can be ultimately attributed to the relativistic enhanced stability of the 6s orbital of Au, at a variance with the less remarkable electrophilicity of analogous linear copper complexes, featuring higher-lying 4s orbitals.^14^ However, since copper– and gold–aluminyl complexes display a nucleophilic reactivity, where the M center also plays an active role, here the same features penalize gold with respect to copper and explain the more favorable reactivity of **3-Cu** with the alkyne substrate.

This unconventional situation is not uniquely related to the first part of the *syn* path, but it affects the whole reaction path. For instance, the reduced ability of gold in the aluminyl complex to interact with the alkyne has been investigated by optimizing a “reactant-like” species with the alkyne coordinated to Au and Cu in a η^2^ mode (see **RC**^**Cu**^ and **RC**^**Au**^ in Figure S1 in the SI) which shows a much more unstable complex (Δ*G* = 18.0 kcal/mol with respect to the separated reactants) formed by **1-Au** compared to that formed by **3-Cu** (Δ*G* = 8.0 kcal/mol).

Furthermore, at variance with **3-Cu**, for **1-Au** isomerization of the **PC’**_**syn**_ to **PC**_**anti**_ species can directly occur via the **TS’**_**syn/anti**_ transition state (green line, Figure 5) with a free-energy barrier Δ*G*^≠^ = 16.2 kcal/mol, remarkably close to that for the formation of **PC’**_**syn**_ (Δ*G*^≠^ = 16.9 kcal/mol). Attempts to calculate a similar direct path for **3-Cu** have led to a minimum energy species with a similar structure to that of **TS’**_**syn/anti**_ (see **PC’**_**anti**_ in Figure S1 in the SI), representing an additional isomer product of the alkyne insertion reaction.

For the *syn/anti* conversion through the two-step path, a higher activation barrier for the first step (“side reaction”) leading to (NON)AlEt and gold acetylide co-product **PC**_**side**_ has been calculated (Δ*G*^≠^ = 31.1 kcal/mol), indicating that the migration of the Et group is not the preferred route to **PC**_**anti**_. In addition, formation of **PC**_**side**_ is endoergonic by 6.2 kcal/mol and dissociation into (NON)AlEt and [*^t^*Bu_3_PAuCCEt] separated species releases 2.5 kcal/mol (see Figure S1). Comparison between the corresponding **PC**_**side**_ thermodynamic stabilities for **3-Cu** and **1-Au** (−9.7 vs +5.9 kcal/mol) gives further indirect evidence supporting a less reactive gold site at **1-Au**, while **3-Cu** is capable of more strongly interacting with unsaturated CC bonds in this uncoventional nucleophilic reactivity. Finally, the **PC**_**side**_ conversion to **PC**_**anti**_ involving the Et group migration to the alkyne (via **TS**_**syn/anti**_) has an activation energy of Δ*G*^≠^ = 25.1 kcal/mol. As a result, one can expect that alkyne insertion into **1-Au** would furnish a possibly isolable *anti* insertion product, with the *syn* product and the *side* (NON)AlEt product which could be more difficult to isolate and characterize, unless more forcing experimental conditions (higher temperatures and longer reaction times) are used.

This detailed mechanistic and orbital picture provides evidence that the exceptional gold Lewis acid standard reactivity (i.e., the ability of gold to promote insertion of π substrate through an inner-sphere or outer-sphere mechanism, see Scheme 3a,b) is switched in the nucleophilic M–Al scenario. This is consistent with neither an inner- or an outer-sphere mechanism, but it is best described as a cooperative radical-like mechanism (see Scheme 3c), where the actual nucleophile is the metal–aluminyl bond, analogously to what happens in the reactivity with carbon dioxide. This new mode of chemical reactivity by coinage metal complexes toward small molecules opens new perspectives beyond the framework of conventional properties, reactivity, and chemical behavior of coinage metal complexes.

## Conclusions

In this work, the reaction mechanism of the alkyne insertion into the Cu–Al bond has been computationally investigated, based on the reported experimental characterization of the reactivity of a copper–aluminyl complex toward 3-hexyne.

The reaction mechanism we find here for the Cu–Al complex nicely agrees with the experimental observations. The calculations predict the activation barrier leading to the *syn* insertion product, where the alkyne approaches the complex closely to the copper site, to be significantly lower than that leading to the *anti* insertion product, in agreement with the experimentally observed kinetic control over *syn* product formation. Electronic structure analysis rationalizes the preferential *syn* formation, showing that the *anti* path is disfavored due to the substantial copper–aluminyl bond breaking occurring at the first transition state, which increases the distortion penalty and, consequently, the associated activation barrier.

The reaction mechanism calculated for the gold–aluminyl complex is qualitatively similar, predicting analogous kinetic control and preferential *syn* product formation due to energy penalty associated to early Au–Al bond breaking along the *anti* reaction path. However, alkyne insertion into the Au–Al bond is expected to furnish a less stable *syn* product, via an almost thermoneutral step, which could be more difficult to isolate and characterize. Interestingly, the mechanism reported here shows that the reaction is predicted to be less efficient for gold than for copper, in sharp contrast with the more efficient electrophilic reactivity of gold complexes toward unsaturated substrates. Extensive electronic structure analysis demonstrates that, in strict analogy with the reactivity reported for gold–aluminyl complexes toward CO_2_, the driving force of the complex–alkyne interaction is the charge transfer from the electron-sharing copper– and gold–aluminyl bonds toward the π* LUMO of the alkyne. The Cu–Al and Au–Al bonds act as actual nucleophilic sites for the reaction, leading to a cooperative radical-like metal–aluminyl reactivity.

This nucleophilic behavior of M–Al bonds leads to a switch in the well-established paradigm for “standard” electrophilic reactivity at the coinage metal site toward unsaturated substrates. Upon thorough analysis, the generally accepted factor determining superior electrophilicity of gold complexes in this context (i.e., the relativistic stabilized valence 6s orbital) is found to lead here to a less nucleophilic Au–Al bond (where the valence Au configuration is close to 6s^1^), leading to a less efficient charge transfer toward the alkyne and, in turn, to a less efficient reactivity.

These findings show that these new classes of copper and gold complexes bearing new-generation aluminyl ligands represent a new chapter in the coinage metal chemistry, where the nucleophilic behavior of the metal–aluminyl bonds induces a paradigm switch of established electronic features and, consequently, different trends in the reactivity with well-known substrates such as alkynes. Furthermore, the results reported here, showing a cooperative radical-like reactivity of the M–Al complexes toward 3-hexyne analogous to that toward carbon dioxide, suggest that these complexes may efficiently react with a range of other small molecules, opening new and unexplored paths for the reactivity of coinage metal complexes.

## Computational Details

All geometry optimizations and frequency calculations on optimized structures (minima with zero imaginary frequencies and transition states with one imaginary frequency) for the alkyne insertion into the [*^t^*Bu_3_PMAl(NON)] (M = Cu,Au) complexes have been carried out using the Amsterdam Density Functional (ADF) code^53,54^ in combination with the related Quantum-regions Interconnected by Local Description (QUILD) program.^55^ The PBE^56^ GGA exchange-correlation (XC) functional, the TZ2P basis set with a small frozen core approximation for all atoms, the ZORA Hamiltonian^57−59^ for treating scalar relativistic effects, and the Grimme’s D3-BJ dispersion correction were used.^60,61^ Solvent effects were modeled employing the conductor-like screening model (COSMO) with the default parameters for toluene as implemented in the ADF code.^62^ The same computational setup has also been used for the EDA, CD-NOCV, and ASM calculations and for computing the radical reactions between [Al(NON)], [EtCCEt], and [*^t^*Bu_3_PM] fragments. This protocol has been used successfully in refs (1, 5) to study the [*^t^*Bu_3_PAuAl(NON)] and [*^t^*Bu_3_PAuCO_2_Al(NON)] complexes reactivity. For further details and description of the methods used in this work, see the “Methodology” section in the Supporting Information.

## Supporting Information Available

Methodology, supporting tables (EDA results for **3-Cu** and **1-Au**, ASM results for **3-Cu** and **1-Au**, and analysis of the products formation from copper and aluminyl fragments and from gold and aluminyl fragments), figures (optimized structures with Cu/Au–alkyne direct interaction and NOCV isosurfaces of the main interactions at the TSs for **3-Cu** and **1-Au**) and scheme for the products formation via metal fragments, and *xyz* coordinates of all structures (PDF)

## References

[ref1] Hicks J.; Mansikkamäki A.; Vasko P.; Goicoechea J. M.; Aldridge S. A Nucleophilic Gold Complex. Nat. Chem. 2019, 11, 237–241. 10.1038/s41557-018-0198-1. 30664716

[ref2] McManus C.; Hicks J.; Cui X.; Zhao L.; Frenking G.; Goicoechea J. M.; Aldridge S. Coinage Metal Aluminyl Complexes: Probing Regiochemistry and Mechanism in the Insertion and Reduction of Carbon Dioxide. Chem. Sci. 2021, 12, 13458–13468. 10.1039/D1SC04676D. 34777765 PMC8528051

[ref3] Liu H. Y.; Schwamm R. J.; Hill M. S.; Mahon M. F.; McMullin C. L.; Rajabi N. A. Ambiphilic Al–Cu Bonding. Angew. Chem., Int. Ed. 2021, 60, 14390–14393. 10.1002/anie.202104658. PMC8252794 33899319

[ref4] Liu H. Y.; Neale S. E.; Hill M. S.; Mahon M. F.; McMullin C. L. On the Reactivity of Al-Group 11 (Cu, Ag, Au) Bonds. Dalton Trans. 2022, 51, 3913–3924. 10.1039/D2DT00404F. 35169824

[ref5] Sorbelli D.; Belpassi L.; Belanzoni P. Reactivity of a Gold-Aluminyl Complex with Carbon Dioxide: A Nucleophilic Gold?. J. Am. Chem. Soc. 2021, 143, 14433–14437. 10.1021/jacs.1c06728. 34472349 PMC8447181

[ref6] Sorbelli D.; Belpassi L.; Belanzoni P. What Singles out Aluminyl Anions? A Comparative Computational Study of the Carbon Dioxide Insertion Reaction in Gold-Aluminyl, -Gallyl, and -Indyl Complexes. Inorg. Chem. 2022, 61, 1704–1716. 10.1021/acs.inorgchem.1c03579. 34986633 PMC8790757

[ref7] Sorbelli D.; Belpassi L.; Belanzoni P. Unraveling Differences in Aluminyl and Carbene Coordination Chemistry: Bonding in Gold Complexes and Reactivity with Carbon Dioxide. Chem. Sci. 2022, 13, 4623–4634. 10.1039/D2SC00630H. 35656139 PMC9020189

[ref8] Sorbelli D.; Rossi E.; Havenith R. W. A.; Klein J. E. M. N.; Belpassi L.; Belanzoni P. Gold-Aluminyl and Gold-Diarylboryl Complexes: Bonding and Reactivity with Carbon Dioxide. Inorg. Chem. 2022, 61, 7327–7337. 10.1021/acs.inorgchem.2c00174. 35512414 PMC9115750

[ref9] McManus C.; Crumpton A. E.; Aldridge S. Alkyne Insertion into Cu–Al Bonds and Selective Functionalization to Form Copper Acyl Compounds. Chem. Commun. 2022, 58, 8274–8277. 10.1039/D2CC02578G. 35790119

[ref10] Joost M.; Gualco P.; Mallet-Ladeira S.; Amgoune A.; Bourissou D. Direct Syn Insertion of Alkynes and Allenes into Au-Si Bonds. Angew. Chem., Int. Ed. 2013, 52, 7160–7163. 10.1002/anie.201303450. 23754788

[ref11] Joost M.; Zeineddine A.; Estévez L.; Mallet-Ladeira S.; Miqueu K.; Amgoune A.; Bourissou D. Facile Oxidative Addition of Aryl Iodides to Gold(I) by Ligand Design: Bending Turns on Reactivity. J. Am. Chem. Soc. 2014, 136, 14654–14657. 10.1021/ja506978c. 25268830

[ref12] Suzuki A.; Wu L.; Lin Z.; Yamashita M. Isomerization of a Cis-(2-Borylalkenyl) Gold Complex via a Retro-1,2-Metalate Shift: Cleavage of a C–C/C–Si Bond Trans to a C–Au Bond. Angew. Chem., Int. Ed. 2021, 60, 21007–21013. 10.1002/anie.202108530. 34288308

[ref13] Suzuki A.; Guo X.; Lin Z.; Yamashita M. Nucleophilic Reactivity of the Gold Atom in a Diarylborylgold(I) Complex toward Polar Multiple Bonds. Chem. Sci. 2021, 12, 917–928. 10.1039/D0SC05478J. PMC8179162 34163858

[ref14] Gorin D. J.; Toste F. D. Relativistic Effects in Homogeneous Gold Catalysis. Nature 2007, 446, 395–403. 10.1038/nature05592. 17377576

[ref15] Ciancaleoni G.; Belpassi L.; Zuccaccia D.; Tarantelli F.; Belanzoni P. Counterion Effect in the Reaction Mechanism of NHC Gold(I)-Catalyzed Alkoxylation of Alkynes: Computational Insight into Experiment. ACS Catal. 2015, 5, 803–814. 10.1021/cs501681f.

[ref16] Gatto M.; Belanzoni P.; Belpassi L.; Biasiolo L.; Del Zotto A.; Tarantelli F.; Zuccaccia D. Solvent-, Silver-, and Acid-Free NHC-Au-X Catalyzed Hydration of Alkynes. The Pivotal Role of the Counterion. ACS Catal. 2016, 6, 7363–7376. 10.1021/acscatal.6b01626.

[ref17] Gaggioli C. A.; Ciancaleoni G.; Zuccaccia D.; Bistoni G.; Belpassi L.; Tarantelli F.; Belanzoni P. Strong Electron-Donating Ligands Accelerate the Protodeauration Step in Gold(I)-Catalyzed Reactions: A Quantitative Understanding of the Ligand Effect. Organometallics 2016, 35, 2275–2285. 10.1021/acs.organomet.6b00346.

[ref18] D’Amore L.; Ciancaleoni G.; Belpassi L.; Tarantelli F.; Zuccaccia D.; Belanzoni P. Unraveling the Anion/Ligand Interplay in the Reaction Mechanism of Gold(I)-Catalyzed Alkoxylation of Alkynes. Organometallics 2017, 36, 2364–2376. 10.1021/acs.organomet.7b00377.

[ref19] Sorbelli D.; Segato J.; Del Zotto A.; Belpassi L.; Zuccaccia D.; Belanzoni P. The Mechanism of the Gold(I)-Catalyzed Meyer–Schuster Rearrangement of 1-Phenyl-2-Propyn-1-ol via 4-Endo-Dig Cyclization. Dalton Trans. 2021, 50, 5154–5160. 10.1039/D1DT00080B. 33710232

[ref20] Kovács G.; Lledõs A.; Ujaque G. Hydroamination of Alkynes with Ammonia: Unforeseen Role of the Gold(I) Catalyst. Angew. Chem., Int. Ed. 2011, 50, 11147–11151. 10.1002/anie.201105309. 21956873

[ref21] Liu X. Y.; Guo Z.; Dong S. S.; Li X. H.; Che C. M. Highly Efficient and Diastereoselective Gold(I)-Catalyzed Synthesis of Tertiary Amines from Secondary Amines and Alkynes: Substrate Scope and Mechanistic Insights. Chem. -Eur. J. 2011, 17, 12932–12945. 10.1002/chem.201101982. 22012740

[ref22] Comas-Vives A.; Ujaque G. Unraveling the Pathway of Gold(I)-Catalyzed Olefin Hydrogenation: An Ionic Mechanism. J. Am. Chem. Soc. 2013, 135, 1295–1305. 10.1021/ja305630z. 23214503

[ref23] Tsui E. Y.; Müller P.; Sadighi J. P. Reactions of a Stable Monomeric Gold(I) Hydride Complex. Angew. Chem., Int. Ed. 2008, 47, 8937–8940. 10.1002/anie.200803842. 18855961

[ref24] Weber D.; Tarselli M. A.; Gagné M. R. Mechanistic Surprises in the Gold(I)-Catalyzed Intramolecular Hydroarylation of Allenes. Angew. Chem., Int. Ed. 2009, 48, 5733–5736. 10.1002/anie.200902049. PMC2978329 19562821

[ref25] Hashmi A. S. K. Isolable Vinylgold Intermediates — First Access to Phantoms of Homogeneous Gold Catalysis. Gold Bull. 2009, 42, 275–279. 10.1007/BF03214949.

[ref26] Zeng X.; Kinjo R.; Donnadieu B.; Bertrand G. Serendipitous Discovery of the Catalytic Hydroammoniumation and Methylamination of Alkynes. Angew. Chem., Int. Ed. 2010, 49, 942–945. 10.1002/anie.200905341. PMC2871156 20058283

[ref27] Gandon V. Modern Gold Catalyzed Synthesis. Edited by A. Stephen K. Hashmi and F. Dean Toste. Angew. Chem., Int. Ed. 2012, 51, 11200 10.1002/anie.201207733.

[ref28] Oonishi Y.; Gómez-Suμrez A.; Martin A. R.; Nolan S. P. Hydrophenoxylation of Alkynes by Cooperative Gold Catalysis. Angew. Chem., Int. Ed. 2013, 52, 9767–9771. 10.1002/anie.201304182. 23897673

[ref29] Johnson M. W.; Shevick S. L.; Toste F. D.; Bergman R. G. Preparation and Reactivity of Terminal Gold(I) Amides and Phosphides. Chem. Sci. 2013, 4, 1023–1027. 10.1039/C2SC21519E.

[ref30] Hesp K. D.; Stradiotto M. Stereo- and Regioselective Gold-Catalyzed Hydroamination of Internal Alkynes with Dialkylamines. J. Am. Chem. Soc. 2010, 132, 18026–18029. 10.1021/ja109192w. 21133383

[ref31] Shi Y.; Ramgren S. D.; Blum S. A. Palladium-Catalyzed Carboauration of Alkynes and Palladium Cross-Coupling. Organometallics 2009, 28, 1275–1277. 10.1021/om801206g.

[ref32] Ye H.; Lu Z.; You D.; Chen Z.; Hua Li Z.; Wang H. Frustrated Lewis Pair Induced Boroauration of Terminal Alkynes. Angew. Chem., Int. Ed. 2012, 51, 12047–12050. 10.1002/anie.201206927. 23081911

[ref33] Rasool J. U.; Ali A.; Ahmad Q. N. Recent Advances in Cu-Catalyzed Transformations of Internal Alkynes to Alkenes and Heterocycles. Org. Biomol. Chem. 2021, 19, 10259–10287. 10.1039/D1OB01709H. 34806741

[ref34] Casals-Cruañas È.; González-Belman O. F.; Besalú-Sala P.; Nelson D. J.; Poater A. The preference for dual-gold(I) catalysis in the hydro(alkoxylation vs. phenoxylation) of alkynes. Org. Biomol. Chem. 2017, 15, 6416–6425. 10.1039/C7OB01457K. 28731109

[ref35] Gómez-Suárez A.; Oonishi Y.; Martin A. R.; Vummaleti S.V.C.; Nelson D. J.; Cordes D. B.; Slawin A.M.Z.; Cavallo L.; Nolan S. P.; Poater A. On the mechanism of the digold(I)-hydroxide-catalysed hydrophenoxylation of alkynes. Chem. - Eur. J. 2016, 22, 1125–1132. 10.1002/chem.201503097. 26662656

[ref36] Larsen M. H.; Houk K. N.; Hashmi A.S.K. Dual gold catalysis: stepwise catalyst transfer via dinuclear clusters. J. Am. Chem. Soc. 2015, 137, 10668–10676. 10.1021/jacs.5b05773. 26258807

[ref37] Lazreg F.; Guidone S.; Gómez-Herrera A.; Nahra F.; Cazin C.S.J. Hydrophenoxylation of internal alkynes catalyzed with a heterobimetallic Cu-NHC/Au-NHC system. Dalton Trans. 2017, 46, 2439–2444. 10.1039/C6DT04513H. 28074200

[ref38] Dupuy S.; Gasperini D.; Nolan S. P. Highly efficient gold(I)-catalyzed regio- and stereoselective hydrocarboxylation of internal alkynes. ACS Catal. 2015, 5, 6918–6921. 10.1021/acscatal.5b02090. 29291135 PMC5745071

[ref39] González-Belman O. F.; Jiménez-Halla J. O. C.; Nahra F.; Cazin C. S. J.; Poater A. The role of the metal in the dual-metal catalyzed hydrophenoxylation of diphenylacetylene. Catal. Sci. Technol. 2018, 8, 3638–3648. 10.1039/C8CY00510A.

[ref40] Clark G. R.; Irvine G. J.; Roper W. R.; Wright L. J. Insertion of Ethyne into the Ru-B Bond of a Coordinatively Unsaturated Ruthenium Boryl Complex. X-Ray Crystal Structure of Ru(CH=CH[BOC_6_H_4_O])Cl(CO)(PPh_3_)_2_. Organometallics 1997, 16, 5499–5505. 10.1021/om970618q.

[ref41] Neeve E. C.; Geier S. J.; Mkhalid I. A. I.; Westcott S. A.; Marder T. B. Diboron(4) Compounds: From Structural Curiosity to Synthetic Workhorse. Chem. Rev. 2016, 116, 9091–9161. 10.1021/acs.chemrev.6b00193. 27434758

[ref42] von Hopffgarten M.; Frenking G. Energy Decomposition Analysis. Wiley Interdiscip. Rev.: Comput. Mol. Sci. 2018, 8, 43–62. 10.1002/wcms.1345.

[ref43] Mitoraj M.; Michalak A. Natural Orbitals for Chemical Valence as Descriptors of Chemical Bonding in Transition Metal Complexes. J. Mol. Model. 2007, 13, 347–355. 10.1007/s00894-006-0149-4. 17024408

[ref44] Belpassi L.; Infante I.; Tarantelli F.; Visscher L. The Chemical Bond between Au(I) and the Noble Gases. Comparative Study of NgAuF and NgAu^+^ (Ng = Ar, Kr, Xe) by Density Functional and Coupled Cluster Methods. J. Am. Chem. Soc. 2008, 130, 1048–1060. 10.1021/ja0772647. 18161976

[ref45] Bistoni G.; Rampino S.; Tarantelli F.; Belpassi L. Charge-Displacement Analysis via Natural Orbitals for Chemical Valence: Charge Transfer Effects in Coordination Chemistry. J. Chem. Phys. 2015, 142, 084112 10.1063/1.4908537. 25725717

[ref46] GitHub - BERTHA-4c-DKS/pycubescd https://github.com/BERTHA-4c-DKS/pycubescd. (accessed 2022 -09 -22).

[ref47] Fernández I.; Bickelhaupt F. M. The Activation Strain Model and Molecular Orbital Theory: Understanding and Designing Chemical Reactions. Chem. Soc. Rev. 2014, 43, 4953–4967. 10.1039/C4CS00055B. 24699791

[ref48] Bickelhaupt F. M.; Houk K. N. Analyzing Reaction Rates with the Distortion/Interaction-Activation Strain Model. Angew. Chem., Int. Ed. 2017, 56, 10070–10086. 10.1002/anie.201701486. PMC5601271 28447369

[ref49] Vermeeren P.; van der Lubbe S. C.; Fonseca Guerra C.; Bickelhaupt F. M.; Hamlin T. A. Understanding Chemical Reactivity Using the Activation Strain Model. Nat. Protoc. 2020, 15, 649–667. 10.1038/s41596-019-0265-0. 31925400

[ref50] Pyykkö P. Additive covalent radii for single-, double-, and triple-bonded molecules and tetrahedrally bonded crystals: a summary. J. Phys. Chem. A 2015, 119, 2326–2337. 10.1021/jp5065819. 25162610

[ref51] Wong Z. R.; Schramm T. K.; Loipersberger M.; Head-Gordon M.; Toste F. D. Revisiting the Bonding Model for Gold(I) Species: The Importance of Pauli Repulsion Revealed in a Gold(I)-Cyclobutadiene Complex. Angew. Chem., Int. Ed. 2022, 61, e202202019 10.1002/anie.202202019. PMC9173747 35261142

[ref52] Leach I. F.; Sorbelli D.; Belpassi L.; Belanzoni P.; Havenith R. W.; Klein J. E. How Reduced Are Nucleophilic Gold Complexes? Dalton Trans. 2022, 10.1039/D2DT01694J. PMC9764324 35877065

[ref53] te Velde G.; Bickelhaupt F. M.; Baerends E. J.; Fonseca Guerra C.; van Gisbergen S. J. A.; Snijders J. G.; Ziegler T. Chemistry with ADF. J. Comput. Chem. 2001, 22, 931–967. 10.1002/jcc.1056.

[ref54] ADF Manual ADF Program System Release 2014 1993.

[ref55] Swart M.; Bickelhaupt F. M. QUILD: QUantum-Regions Interconnected by Local Descriptions. J. Comput. Chem. 2008, 29, 724–734. 10.1002/jcc.20834. 17902157

[ref56] Perdew J. P.; Burke K.; Ernzerhof M. Generalized Gradient Approximation Made Simple. Phys. Rev. Lett. 1996, 77, 3865–3868. 10.1103/PhysRevLett.77.3865. 10062328

[ref57] Lenthe E. v.; Baerends E. J.; Snijders J. G. Relativistic Regular Two-Component Hamiltonians. J. Chem. Phys. 1993, 99, 4597–4610. 10.1063/1.466059.

[ref58] Van Lenthe E.; Baerends E. J.; Snijders J. G. Relativistic Total Energy Using Regular Approximations. J. Chem. Phys. 1994, 101, 9783–9792. 10.1063/1.467943.

[ref59] Van Lenthe E.; Ehlers A.; Baerends E. J.; et al. Geometry Optimizations in the Zero Order Regular Approximation for Relativistic Effects. J. Chem. Phys. 1999, 110, 8943–8953. 10.1063/1.478813.

[ref60] Grimme S.; Antony J.; Ehrlich S.; Krieg H. A Consistent and Accurate Ab Initio Parametrization of Density Functional Dispersion Correction (DFT-D) for the 94 Elements H-Pu. J. Chem. Phys. 2010, 132, 154104 10.1063/1.3382344. 20423165

[ref61] Grimme S.; Ehrlich S.; Goerigk L. Effect of the Damping Function in Dispersion Corrected Density Functional Theory. J. Comput. Chem. 2011, 32, 1456–1465. 10.1002/jcc.21759. 21370243

[ref62] Pye C. C.; Ziegler T. An Implementation of the Conductor-like Screening Model of Solvation within the Amsterdam Density Functional Package. Theor. Chem. Acc. 1999, 101, 396–408. 10.1007/s002140050457.

