# Supplementary material for: Mechanistic Study of Alkyne Insertion into Cu–Al and Au–Al Bonds: A Paradigm Shift for Coinage Metal Chemistry
Source: Inorg Chem. 2022 Dec 9;61(51):21095–106. doi: 10.1021/acs.inorgchem.2c03713 (PMC9795551; doi:10.1021/acs.inorgchem.2c03713)
Supplement: Supplementary file 1 — ic2c03713_si_001.pdf [file ic2c03713_si_001.pdf]

Electronic Supporting Information

Mechanistic study of alkyne insertion into Cu-Al and Au-Al bonds: a paradigm shift for coinage metal chemistry

Diego Sorbelli,[*ab] Leonardo Belpassi,[*b] and Paola Belanzoni[*ab]

[a]*Department of Chemistry, Biology and Biotechnologies, University of Perugia, Via Elce di Sotto, 8 – 06123, Perugia, Italy*

[b]*CNR Institute of Chemical Science and Technologies "Giulio Natta" (CNR-SCITEC), Via Elce di Sotto, 8 – 06123, Perugia, Italy*

Contents

Methodology **2**
Computational Details **6**
Figure S1. Optimized structures with Cu/Au-alkyne direct interaction **8**
Table S1. EDA results (3-Cu) **8**
Figures S2-S7. NOCV results at TSs (3-Cu) **9**
Table S2. ASM results (3-Cu) **15**
Scheme S1. Scheme for the formation of the products via metal fragments **15**
Table S3. Analysis of the products formation from copper and aluminyl fragments **16**
Table S4. EDA results (1-Au) **17**
Table S5. ASM results (1-Au) **17**
Figures S8-S13. NOCV results at TSs (1-Au) **18**
Table S6. Analysis of the products formation from gold and aluminyl fragments **24**
References **25**
xyz geometries **27**

Methodology

- **Natural Orbitals for Chemical Valence and Charge Displacement analysis**

Natural Orbitals for Chemical Valence (NOCV)[1,2] represents a suitable approach for describing the chemical bond. This approach is based on the rearrangement of the electron density occurring when a chemical bond is formed and such rearrangement can be expressed as electron density difference between the formed adduct (AB) and sum of the densities of the two non-interacting fragments (A and B) frozen in their adduct geometry.

This deformation density can be brought into diagonal contributions in terms of NOCVs. In the NOCV scheme, the charge rearrangement taking place upon bond formation is obtained from the occupied orbitals of the two fragments suitably orthogonalized to each other and renormalized (*promolecule*). The resulting electron density rearrangement ($\Delta\rho'$) can be expressed in terms of NOCV pairs which are defined as the eigenfunctions of the so-called "valence operator"[3–5] as follows:

$$\boldsymbol{\Delta\rho'} = \sum_{k} \boldsymbol{\nu_k}(|\boldsymbol{\phi}_{+\mathrm{k}}|^2 - |\boldsymbol{\phi}_{\text{-}\mathrm{k}}|^2) = \sum_{k} \boldsymbol{\Delta\,\rho'_k} \qquad \text{[S1]}$$

where ϕ_{+k} and ϕ_{-k} are the NOCV pairs orbitals and $\nu_{\pm k}$ are the corresponding eigenvalues. Upon formation of the adduct from the promolecule, a fraction ν_k of electrons is transferred from the ϕ_{-k} to the ϕ_{-k} orbital (donor and acceptor orbitals, respectively).

The NOCV scheme can be coupled with the framework of the Charge Displacement (CD)[6] analysis. The CD analysis allows to quantify the amount of electronic charge that is transferred between the two fragments upon the formation of the A-B bond. The Charge Displacement function (Δq) can be defined as the partial progressive integration on a suitable z-axis of the deformation density $\Delta\rho'$:[7]

$$\boldsymbol{\Delta q(z)} = \int_{-\infty}^{z} \boldsymbol{dz'} \int_{-\infty}^{+\infty}\int_{-\infty}^{+\infty} \boldsymbol{\Delta\,\rho'(x,y,z')dxdy} \qquad \text{[S2]}$$

The CD function, Δq(z), quantifies at each point of the chosen z-axis (which usually corresponds to the bond axis) the exact amount of electron charge that, upon formation of the bond, is transferred from the right to the left across a plane perpendicular to the bond axis through z.

When coupled with the NOCV scheme, the density rearrangement due to the bond formation between two fragments, ($\Delta\rho'$), is partitioned in different NOCV deformation densities ($\Delta\rho'_k$) and therefore one is able to quantify the charge transfer (CT) associated to the components. Note that only few of the

NOCV pairs contributes to the chemical bond. Therefore, when the CD-NOCV analysis is carried out, usually only the first $\Delta\rho_k'$ components are investigated in order to understand which significant chemical contribution to the bond they represent.

Usually we choose to evaluate the charge transfer between A and B by taking the CD value at the "isodensity boundary", i.e. the z-point where equally valued isodensity surfaces of the isolated fragments become tangent.[7,8]

When we apply this scheme at the TSs with [^{t}Bu$_3$PMAl(NON)] (M=Cu,Au) and [EtCCEt] as fragments, such approach becomes complicated, since the two fragments display multiple interactions with multiple atomic centres and thus it is clearly impossible to define a unique bond axis and it is very hard to rely on the isodensity boundary for the estimation of the charge transfer. In order to avoid any ambiguity in the definition of the z-axis, we recall an approach that may be useful for evaluating the charge transferred between the [^{t}Bu$_3$PMAl(NON)] and [EtCCEt] fragments .[9]

Within this approach, the electron density rearrangement ($\Delta\rho'$), which typically shows charge accumulation regions (positive values) and charge depletion regions (negative values), defines two different positive functions, $\Delta\rho^+$ and $\Delta\rho^-$, each equal to the magnitude of the appropriate portion, *i.e.*:

$$\boldsymbol{\Delta\rho^{+/-}(r) = max[\pm\Delta\rho(r)', 0]} \quad \text{[S3]}$$

so that

$$\boldsymbol{\Delta\rho(r)' = \Delta\rho^{+}(r) - \Delta\rho^{-}(r)} \quad \text{[S4]}$$

By defining two arbitrary regions that are associated with the interacting fragments, we can evaluate the charge transfer as follows:

$$\boldsymbol{CT = \int_A \Delta\,\rho(r)'dr = -\int_B \Delta\,\rho(r)'dr} \quad \text{[S5]}$$

By combining Eqs. [S4] and [S5], CT can also be expressed as:

$$\boldsymbol{CT = \int_A \Delta\,\rho^{+}(r)dr - \int_A \Delta\,\rho^{-}(r)dr = -\int_B \Delta\,\rho^{+}(r)dr + \int_B \Delta\,\rho^{-}(r)} \quad \text{[S6]}$$

Ultimately, this approach can also be expressed in the CD-NOCV framework. By combining Equations [S1] and [S5], we can use to this approach for calculating the charge transfer associated to each NOCV deformation density as follows:

$$CT_k = \int_A \Delta\, \rho_k(r)' dr = -\int_B \Delta\, \rho_k(r)' dr \qquad [S7]$$

Despite the spatial regions associated to the two interacting fragments being defined arbitrarily, this approach is particularly suitable for the analysis of the interaction between the [^{t}Bu$_3$PMAl(NON)] and [EtCCEt] fragments at all TSs, being the two fragments well-separated in space.

- **Energy Decomposition Analysis and ETS-NOCV approach**

The Energy Decomposition Analysis (EDA)[10–12] has been used in this work to get additional and complementary insights into the interaction between the alkyne and the [^{t}Bu$_3$PMAl(NON)] complex. With this approach, the interaction energy between the fragments can be decomposed in different contributions as follows:

$$\Delta E_{\text{int}} = \Delta E^{Pauli} + \Delta V_{elst} + \Delta E_{oi} + \Delta E_{disp} \qquad [S8]$$

where ΔE^{Pauli} corresponds the Pauli repulsion interaction between occupied orbitals on the two fragments, ΔV_{elst} represents the quasiclassical electrostatic interaction between the unperturbed charge distribution of the fragments at their final positions, ΔE_{disp} takes into account the dispersion contribution and ΔE_{oi} is the orbital interaction, which arises from the orbital relaxation and the orbital mixing between the fragments, and accounts for electron pair bonding, charge transfer, and polarization.

The orbital interaction term ΔE_{oi} can be further decomposed within the ETS-NOCV[13] scheme into NOCV pairwise orbital contributions ($\Delta E_{oi} = \sum_k \Delta\, E_{oi}^k$) which associates an energy contribution (E_{oi}^k) to each NOCV deformation density ($\Delta\rho_k$).

- **Activation Strain Model**

The Activation Strain Model (ASM)[14–16] allows to decompose the relative energy (ΔE) of each stationary point along the reaction path in two contributions: a penalty arising from the distortion of

the reactants from their relaxed geometry to their in-adduct ones (ΔE_{dist}) and a (usually) stabilizing interaction contributions arising from the interaction between reacting fragments (ΔE_{int}). Thus, ΔE can be expressed as follows:

$$\boldsymbol{\Delta E(\xi) = \Delta E_{\mathrm{dist}}(\xi) + \Delta E_{\mathrm{int}}(\xi)} \qquad [S9]$$

where ξ represents the reaction coordinate. In the case of the two reactants (and interacting fragments) being the complex and the alkyne, the distortion penalty can be expressed as:

$$\begin{gathered}\boldsymbol{\Delta E_{dist}(\xi) = \Delta E_{\mathrm{dist}}^{alkyne}(\xi) + \Delta E_{dist}^{complex}(\xi)} \\ \boldsymbol{\Delta E_{\mathrm{dist}}^{alkyne}(\xi) = E^{alkyne}(\xi) - E_{\mathrm{relaxed}}^{alkyne}} \\ \boldsymbol{\Delta E_{dist}^{complex}(\xi) = E^{complex}(\xi) - E_{\mathrm{relaxed}}^{complex}}\end{gathered} \qquad [S10]$$

where the "$E^{alkyne}(\xi)$" and "$E^{complex}(\xi)$" terms represent the energy of the two fragments evaluated at the geometry assumed by the fragments at selected points along the reaction coordinate, which in this work will be the stationary points. Similarly, the interaction contribution can be expressed as:

$$\boldsymbol{\Delta E_{\mathrm{int}}(\xi) = E(\xi) - E^{complex}(\xi) - E^{alkyne}(\xi)} \qquad [S11]$$

where "$E\ (\xi)$" represents the energy of the stationary point under study.

Computational details

Complexes **1-Au** and **3-Cu** have been slightly simplified at the NON site by replacing the two tert-butyl groups at the peripheral positions of the dimethylxanthene moiety with hydrogen atoms and the two Dipp substituents on the nitrogen atoms with phenyl groups. The effect of the modelling for this class of complexes has been extensively evaluated in Refs. [17,18] where the same computational set up as that used in the present work was applied. Good agreement with experimental data was found for geometries and in general both the reaction mechanism and the electronic structure calculations show negligible deviations due to the structural simplifications used.

All geometry optimizations and frequency calculations on optimized structures (minima with zero imaginary frequencies and transition states with one imaginary frequency) for the reaction of 3-hexyne with the [^{t}Bu$_3$PMAl(NON)] complexes have been carried out using the Amsterdam Density Functional (ADF) code[19,20] in combination with the related Quantum-regions Interconnected by Local Description (QUILD) program.[21] The PBE[22] GGA exchange-correlation (XC) functional, the TZ2P basis set with a small frozen core approximation for all atoms, the ZORA Hamiltonian[23–25] for treating scalar relativistic effects and the Grimme's D3-BJ dispersion correction were used.[26,27] Solvent effects were modelled employing the Conductor-like Screening Model (COSMO) with the default parameters for toluene as implemented in the QUILD code.[28] Effects of the exchange-correlation functional and solvation in this framework have been recently evaluated and are found, overall, to negligibly affect the results.[18] The same computational setup has also been used for the AMS, EDA, CD-NOCV and ETS-NOCV analyses. This computational protocol has been used in ref. [29] to study the [^{t}Bu$_3$PAuAl(NON)] and [^{t}Bu$_3$PAuCO$_2$Al(NON)] complexes and to investigate the mechanisms of the CO_2 insertion reaction in similar compounds featuring gold and Group 13 elements .[17,18,30]

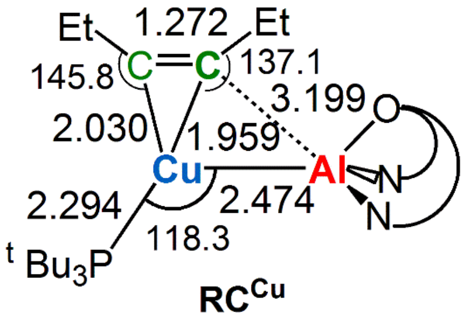

RC^{Cu}

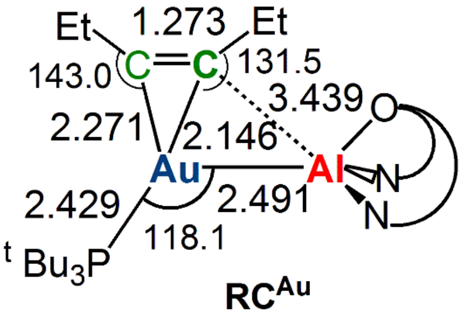

RC^{Au}

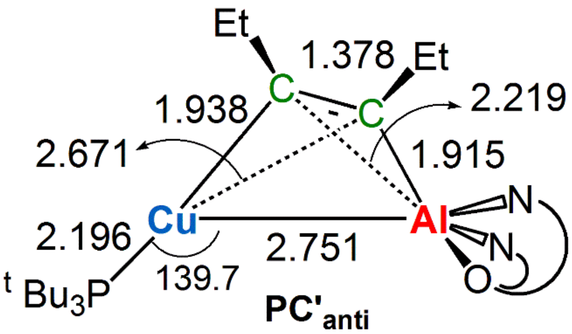

PC'$_{anti}$

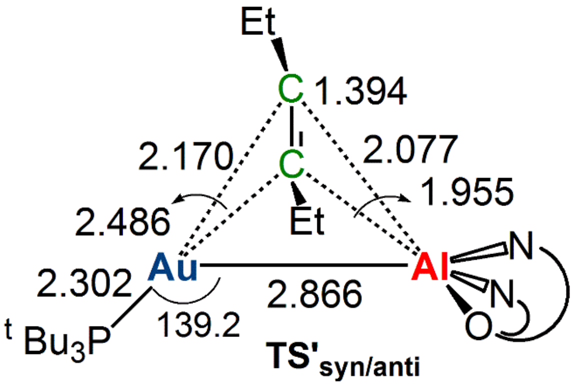

TS'$_{syn/anti}$

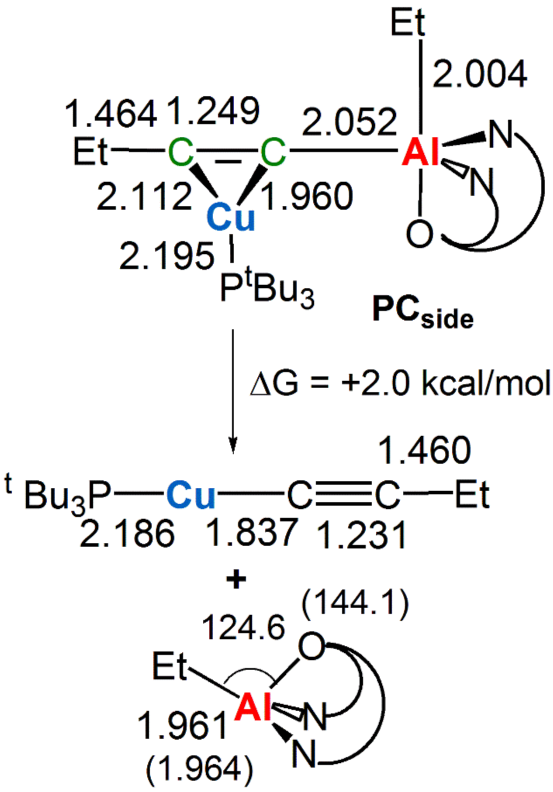

PC$_{side}$

ΔG = +2.0 kcal/mol

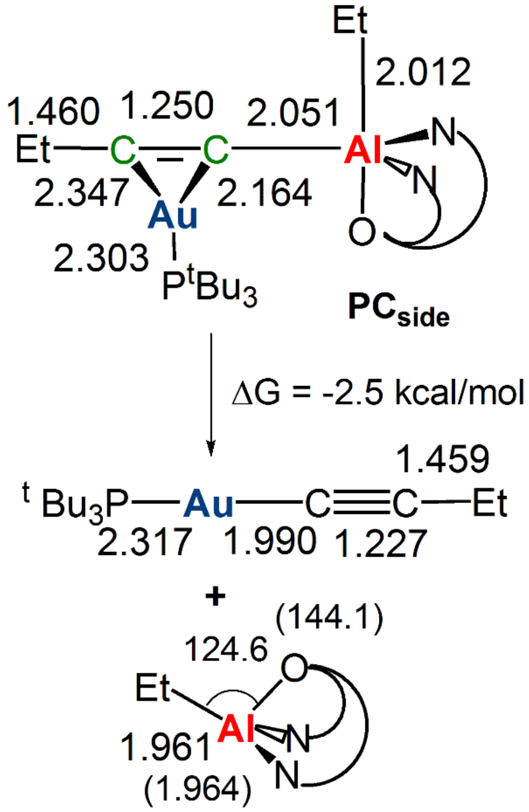

PC$_{side}$

ΔG = -2.5 kcal/mol

Figure S1. Sketched optimized structures involving Cu (right) and Au (left) direct interaction with 3-hexyne. Selected interatomic distances in Å and bond angles in degrees. Available experimental X-ray data are reported in parenthesis.

	TSI_{syn}	TSI_{syn}^{Cu}	TSI_{anti}
**ΔE**	-20.7	-72.2	-82.4
ΔE^{Pauli}	182.5	346.1	449.8
ΔE_{elst}	-108.5	-217.7	-278.2
ΔE_{steric}	73.5	128.4	171.5
ΔE_{oi}	-81.7	-187.6	-238.6
ΔE_{oi}^{1}	-55.3	-140.4	-180.7
$\lvert CT^{1}\rvert$	0.15	0.54	0.66
ΔE_{oi}^{2}	-14.0	-20.1	-23.7
$\lvert CT^{2}\rvert$	0.05	0.10	0.12
ΔE_{disp}	-12.5	-13.0	-15.3

Table S1. Results of the Energy Decomposition Analysis (EDA), ETS-NOCV and CD-NOCV analyses of the [EtC=CEt]-[$^{t}Bu_3PCuAl(NON)$] interaction at **TSI_{syn}**, **TSI_{syn}^{Cu}** and **TSI_{anti}**. All energies are expressed in kcal/mol, charge transfer (CT) values are expressed in electrons.

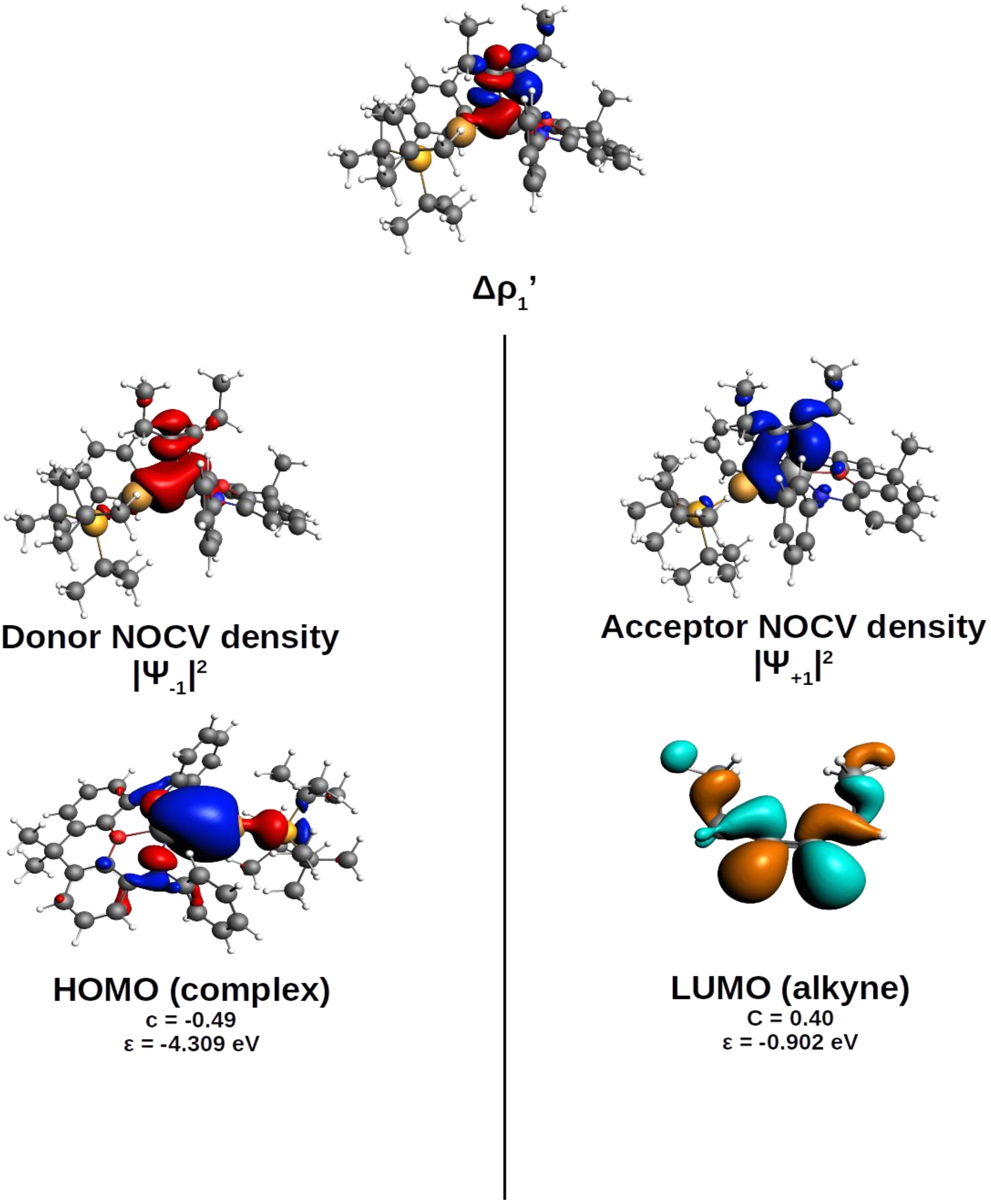

Figure S2. Breakdown of the donor ($|\Psi_1|^2$) and acceptor ($|\Psi_{-1}|^2$) NOCV densities that are associated with the deformation density $\Delta\rho'_1$ in the transition state **TSI**$_{\mathbf{syn}}$ into the most important MOs of the fragments frozen at their **TSI**$_{\mathbf{syn}}$ geometry. The molecular orbitals' mixing coefficients (c) are given together with their energy (ε).

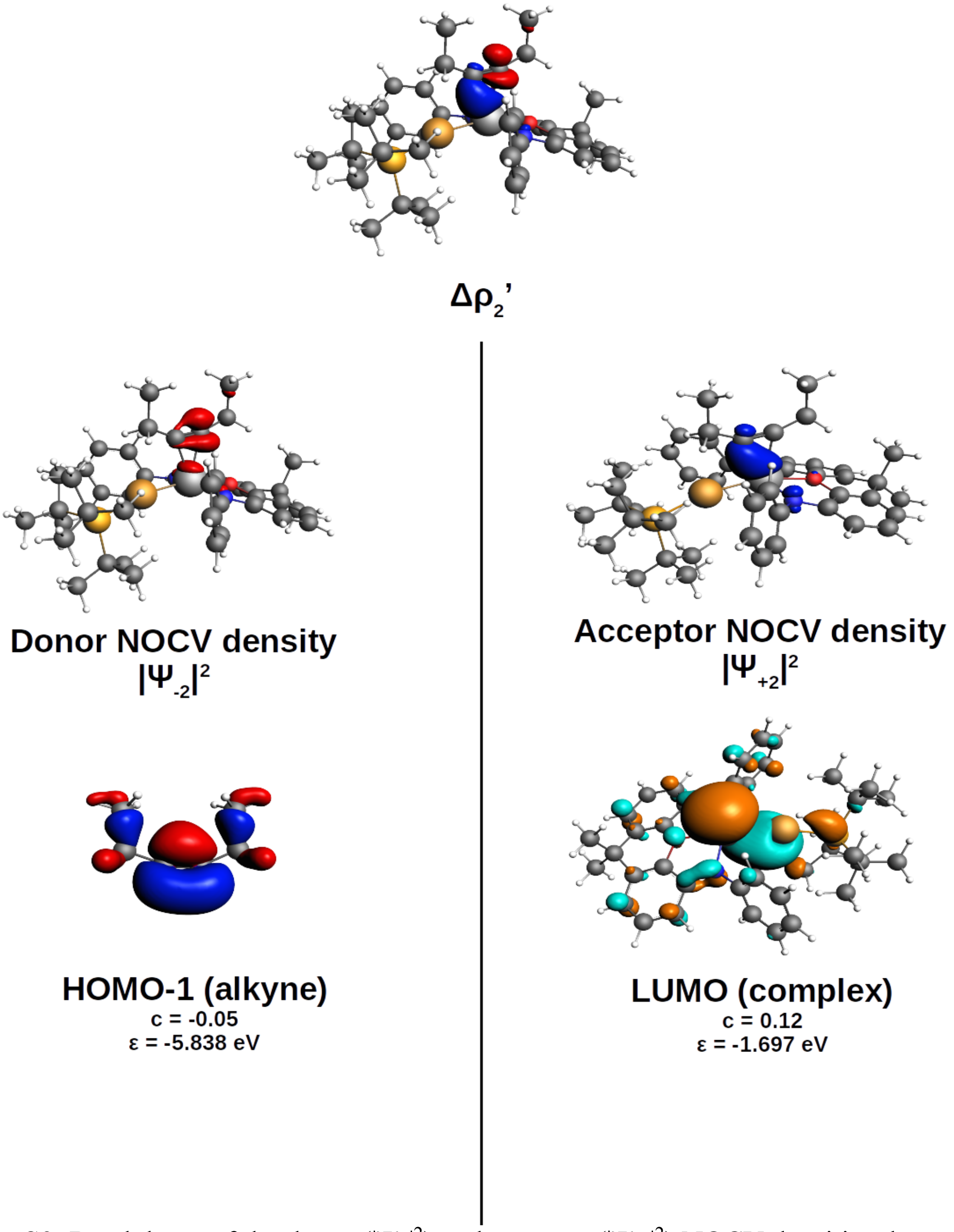

Figure S3. Breakdown of the donor ($|\Psi_2|^2$) and acceptor ($|\Psi_{-2}|^2$) NOCV densities that are associated with the deformation density $\Delta\rho'_2$ in the transition state **TSI**$_{\mathbf{syn}}$ into the most important MOs of the fragments frozen at their **TSI**$_{\mathbf{syn}}$ geometry. The molecular orbitals' mixing coefficients (c) are given together with their energy (ε).

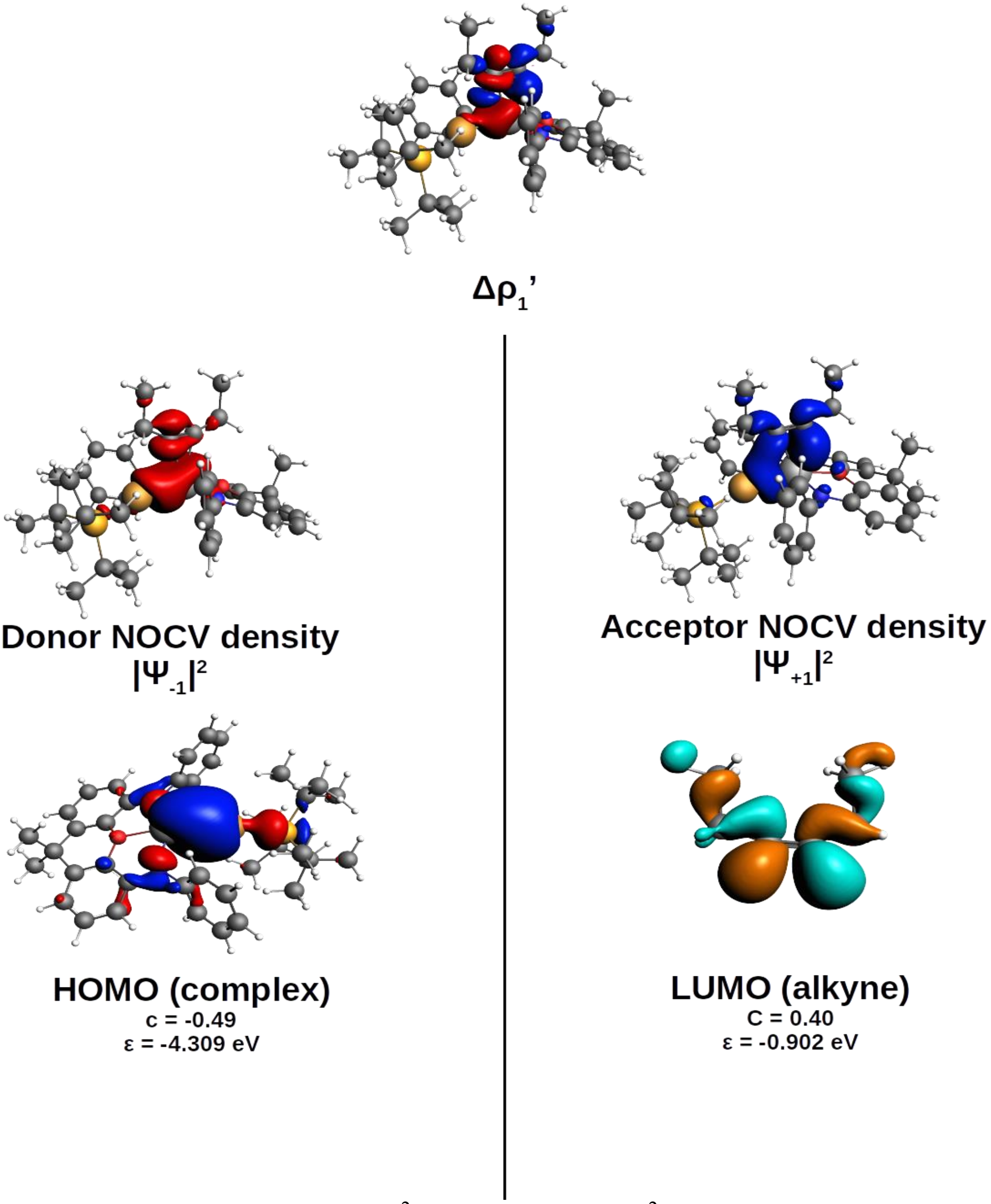

Figure S4. Breakdown of the donor ($|\Psi_1|^2$) and acceptor ($|\Psi_{-1}|^2$) NOCV densities that are associated with the deformation density $\Delta\rho'_1$ in the transition state $\mathbf{TSI_{syn}^{Cu}}$ into the most important MOs of the fragments frozen at their $\mathbf{TSI_{syn}^{Cu}}$ geometry. The molecular orbitals' mixing coefficients (c) are given together with their energy (ε).

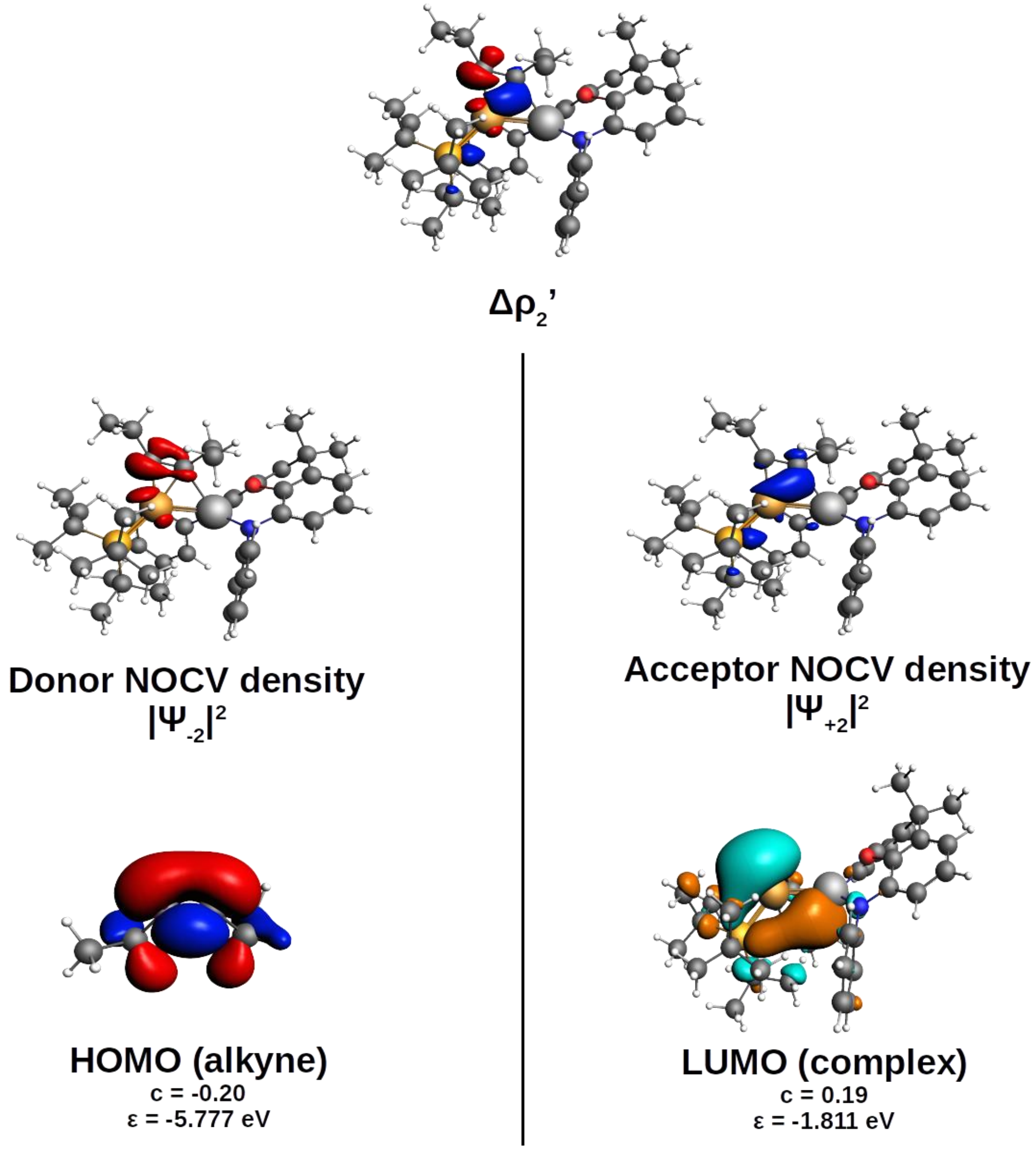

Figure S5. Breakdown of the donor ($|\Psi_2|^2$) and acceptor ($|\Psi_{-2}|^2$) NOCV densities that are associated with the deformation density $\Delta\rho'_2$ in the transition state $\mathbf{TSI_{syn}^{Cu}}$ into the most important MOs of the fragments frozen at their $\mathbf{TSI_{syn}^{Cu}}$ geometry. The molecular orbitals' mixing coefficients (c) are given together with their energy (ε).

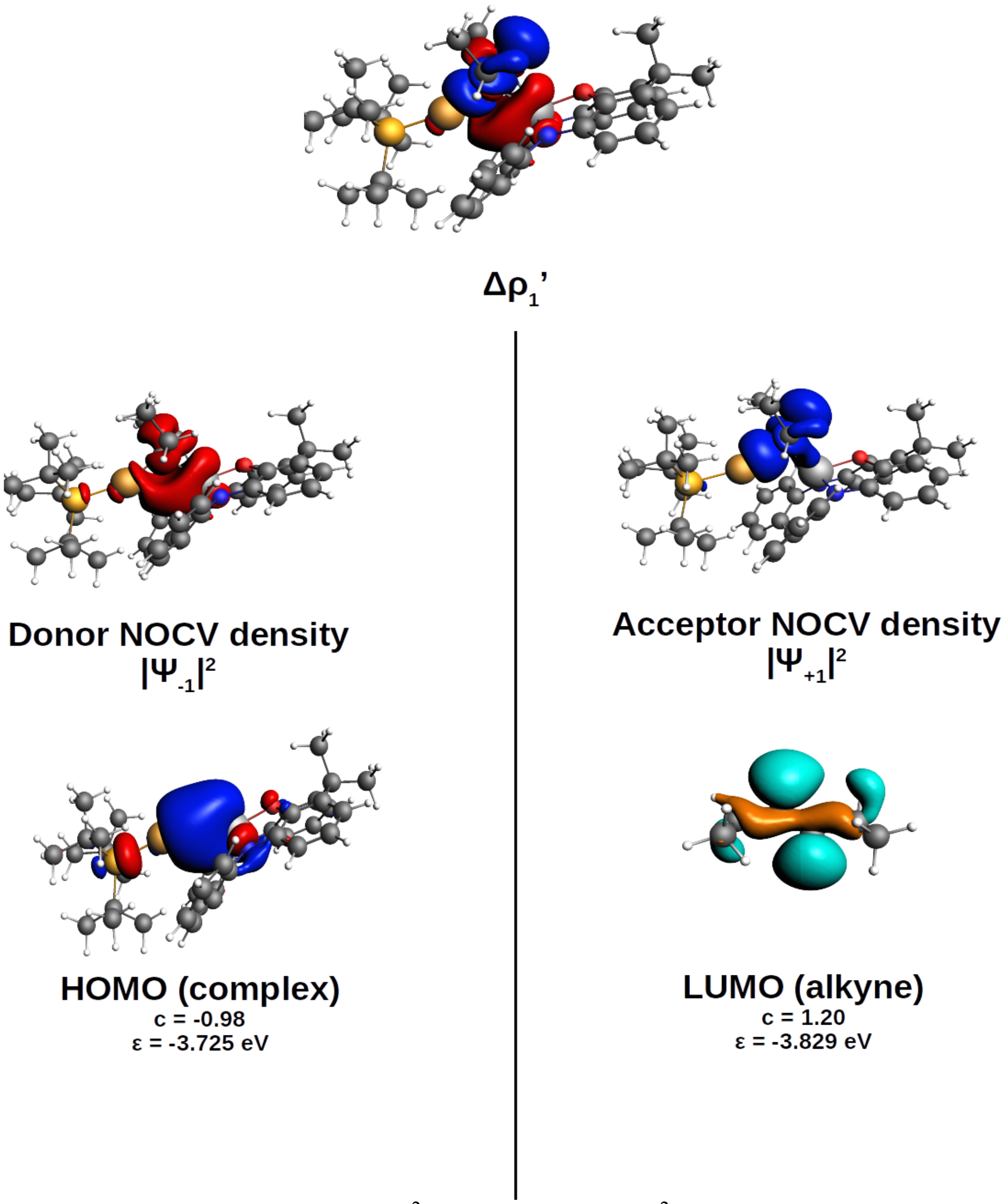

Figure S6. Breakdown of the donor ($|\Psi_1|^2$) and acceptor ($|\Psi_{-1}|^2$) NOCV densities that are associated with the deformation density $\Delta\rho'_1$ in the transition state **TS**$_{\mathbf{anti}}$ into the most important MOs of the fragments frozen at their **TS**$_{\mathbf{anti}}$ geometry. The molecular orbitals' mixing coefficients (c) are given together with their energy (ε).

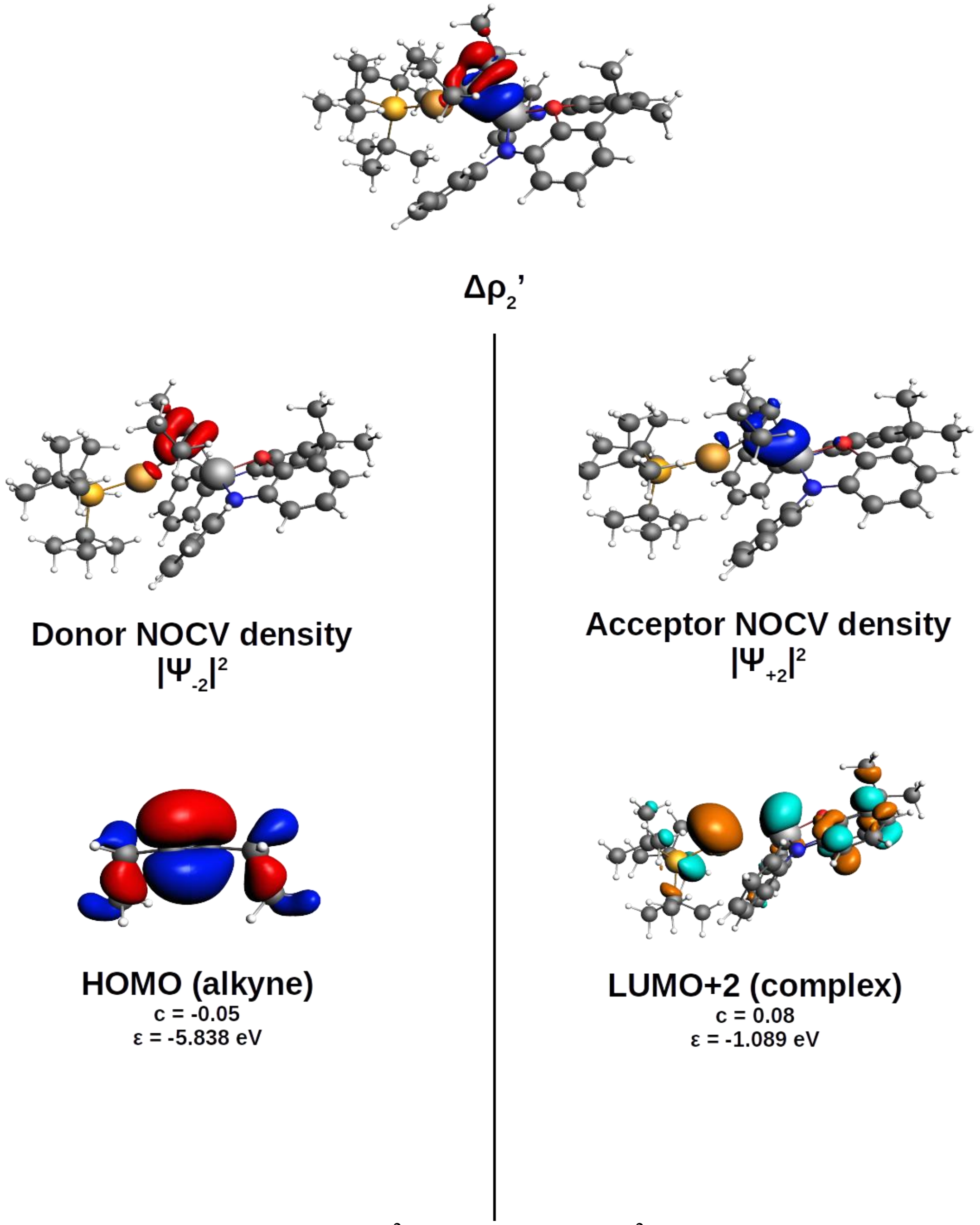

Figure S7. Breakdown of the donor ($|\Psi_2|^2$) and acceptor ($|\Psi_{-2}|^2$) NOCV densities that are associated with the deformation density $\Delta\rho'_2$ in the transition state **TS$_{anti}$** into the most important MOs of the fragments frozen at their **TS$_{anti}$** geometry. The molecular orbitals' mixing coefficients (c) are given together with their energy (ε).

	RC$_{syn}$	TSI$_{syn}$	TSI$_{syn}^{Cu}$	PC'$_{syn}$	PC$_{syn}$	TS$_{side}$	PC$_{side}$	TS$_{syn/anti}$	RC$_{anri}$	TS$_{anti}$	PC$_{anti}$
ΔE	-8.0	4.6	-9.6	-28.5	-23.9	5.4	-24.0	7.8	-13.0	6.4	-28.5
ΔE$_{INT}$	-9.2	-19.6	-72.2	-119.1	-148.5	-154.8	-209.1	-162.0	-13.8	-82.9	-147.3
ΔE$_{DIST}$ alkyne	0.2	10.1	47.7	61.0	74.2	99.9	134.9	107.2	0.4	62.7	52.2
ΔE$_{DIST}$ compl	0.9	14.1	14.9	29.6	50.5	60.3	50.2	62.7	0.4	26.6	66.6
ΔE$_{DIST}$	1.1	24.2	62.6	90.6	124.7	160.2	185.1	169.9	0.8	89.4	118.8

Table S2. Results of the Activation Strain Model (ASM) analysis of the [EtCCEt]-[^{t}Bu$_3$PCuAl(NON)] interaction along the related reaction path for all stationary points. All energies are expressed in kcal/mol.

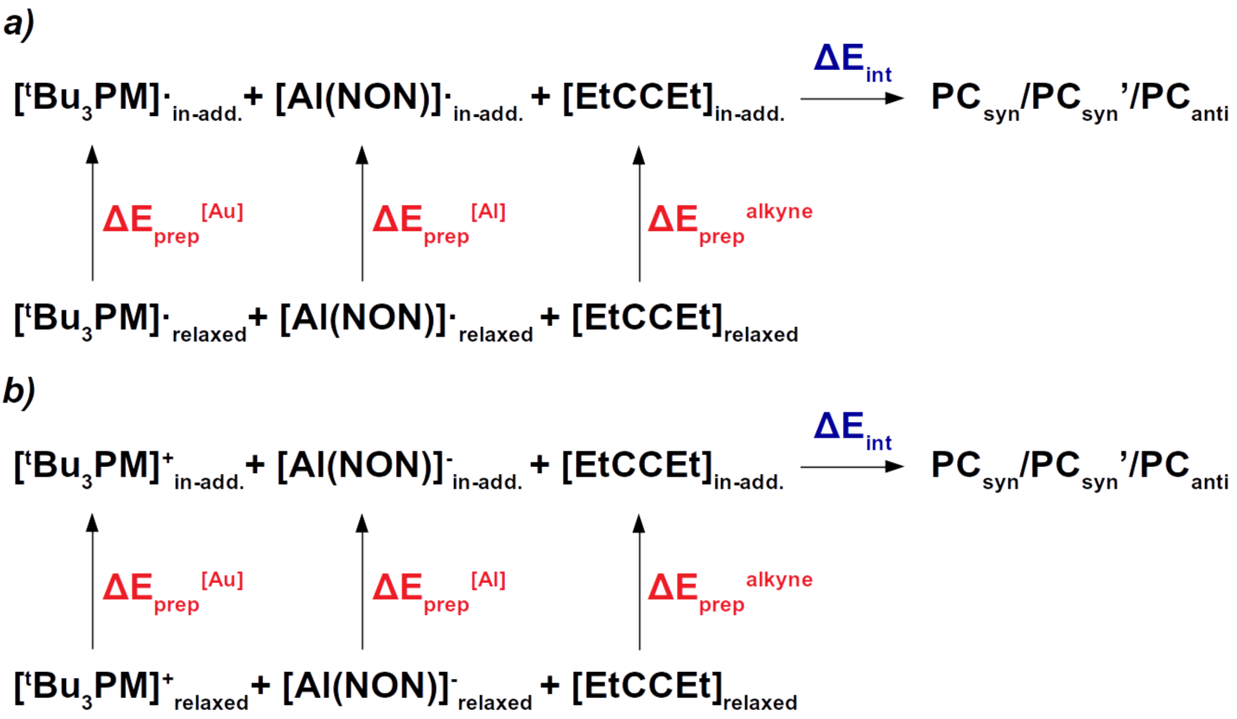

Scheme S1. Scheme for the formation of the *syn* (**PC$_{syn}$**, **PC$_{syn}$'**) and *anti* (**PC$_{anti}$**) products from radical (a) or closed shell (b) [^{t}Bu$_3$PM] (M=Cu,Au), [Al(NON)] and [EtCCEt] fragments.

		PC_{syn}	PC_{syn}'	PC_{anti}
ΔE_{prep}	[$^tBu_3PCu$]·	0.7	0.8	0.5
	[Al(NON)]·	1.8	7.2	0.3
	[EtCCEt]	74.2	61.0	52.2
	Sum	76.7	69.0	53.0
ΔE_{int}		-178.9	-175.9	-159.9
ΔE		-102.2	-106.9	-106.9
ΔE_{prep}	[$^tBu_3PCu$]$^+$	3.6	2.8	2.0
	[Al(NON)]$^-$	8.7	13.9	8.4
	[EtCCEt]	74.2	61.0	52.2
	Sum	86.5	77.8	62.6
ΔE_{int}		-218.1	-214.1	-198.9
ΔE		-131.6	-136.3	-136.3

Table S3. Results of the analysis of the formation of the **PC_{syn}**, **PC_{syn}'** and **PC_{anti}** products from radical ([$^tBu_3Cu$]· and [Al(NON)]·) or closed shell charged ([$^tBu_3Cu$]$^+$ and [Al(NON)]$^-$) fragments. All energies are reported in kcal/mol.

	**TSI_{syn}**	**TSI_{syn}^{Au}**	**TSI_{anti}**
**ΔE**	-24.7	-49.8	-77.2
**ΔE^{Pauli}**	190.0	357.0	446.3
**ΔE_{elst}**	-111.9	-218.5	-275.2
**ΔE_{steric}**	78.1	138.5	171.1
**ΔE_{oi}**	-90.9	-176.0	-233.3
**ΔE_{oi}^{1}**	-65.7	-127.2	-169.7
**$\lvert CT^{1}\rvert$**	0.15	0.43	0.59
**ΔE_{oi}^{2}**	-11.8	-22.9	-26.3
**$\lvert CT^{2}\rvert$**	0.04	0.09	0.14
**ΔE_{disp}**	-11.9	-12.3	-15.0

Table S4. Results of the Energy Decomposition Analysis (EDA), ETS-NOCV and CD-NOCV analyses of the [EtCCEt]-[$^{t}Bu_3PAuAl$(NON)] interaction at **TSI_{syn}**, **TSI_{syn}^{Au}** and **TSI_{anti}**. All energies are expressed in kcal/mol, charge transfer (CT) values are expressed in electrons.

	**RC_{syn}**	**TSI_{syn}**	**TSI_{syn}^{Au}**	**PC'_{syn}**	**PC_{syn}**	**TS' syn/anti**	**TS_{side}**	**PC_{side}**	**TS syn/anti**	**RC_{anti}**	**TSI_{anti}**	**PC_{anti}**
**ΔE**	-8.5	6.0	14.2	-20.8	1.8	-4.6	16.4	-5.4	18.9	-12.4	14.7	-20.3
**ΔE_{INT}**	-9.3	-23.4	-49.8	-121.5	-42.5	-99.8	-157.6	-202.0	-168.1	-14.0	-77.3	-147.4
**ΔE_{DIST} alkyne**	0.3	13.4	46.2	65.1	18.6	67.0	103.6	134.1	112.8	0.4	59.6	50.6
**ΔE_{DIST} compl**	0.5	16.0	17.8	35.7	25.6	28.1	70.5	62.5	74.2	1.2	32.4	76.5
**ΔE_{DIST}**	0.8	29.4	64.0	100.7	44.3	95.1	174.1	196.6	187.0	1.6	92.0	127.1

Table S5. Results of the Activation Strain Model (ASM) analysis of the [EtCCEt]-[$^{t}Bu_3PAuAl$(NON)] interaction along the related reaction path for all stationary points. All energies are expressed in kcal/mol.

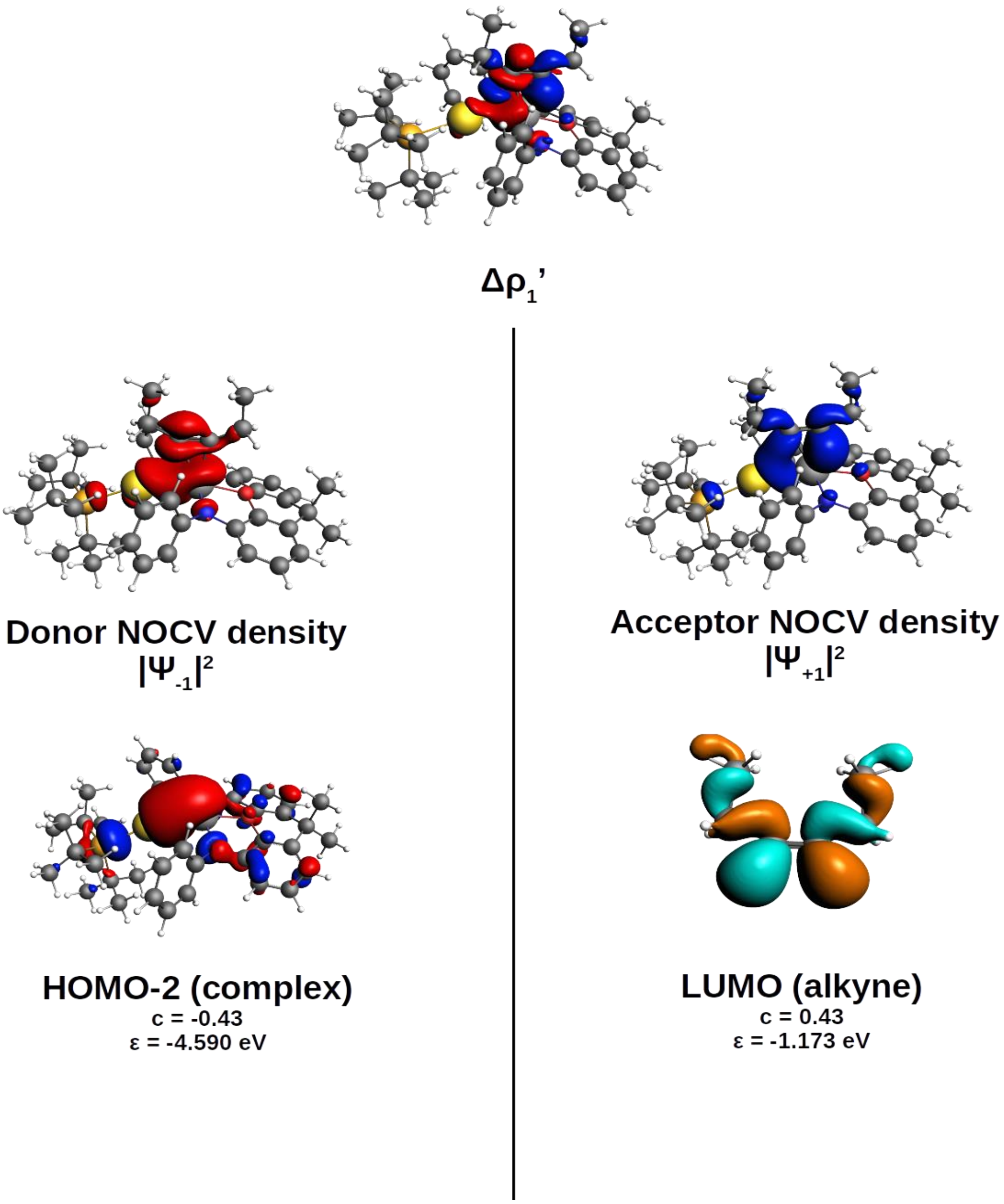

Figure S8. Breakdown of the donor ($|\Psi_1|^2$) and acceptor ($|\Psi_{-1}|^2$) NOCV densities that are associated with the deformation density $\Delta\rho'_1$ in the transition state **TSI$_{syn}$** into the most important MOs of the fragments frozen at their **TSI$_{syn}$** geometry. The molecular orbitals' mixing coefficients (c) are given together with their energy (ε).

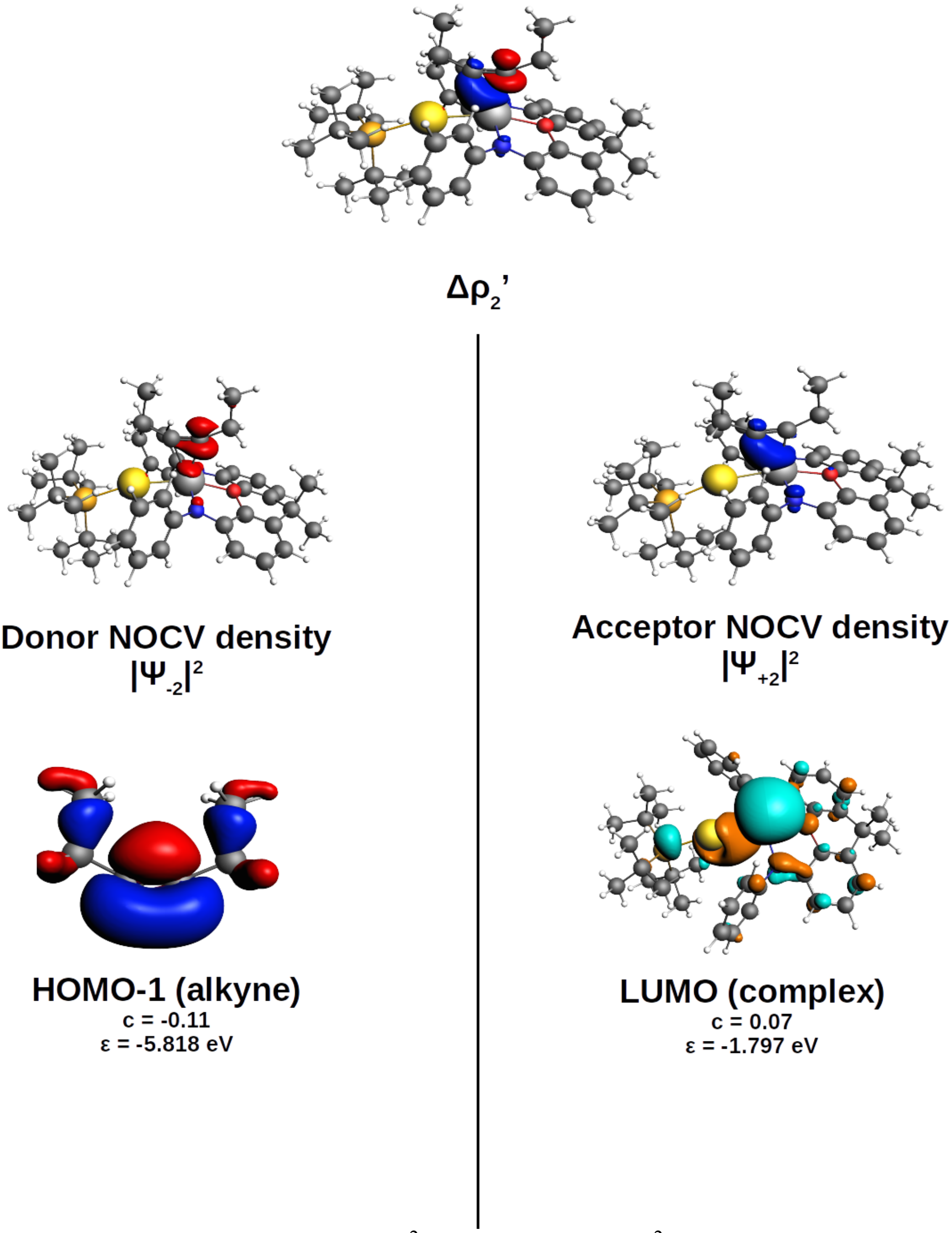

Figure S9. Breakdown of the donor ($|\Psi_2|^2$) and acceptor ($|\Psi_{-2}|^2$) NOCV densities that are associated with the deformation density $\Delta\rho'_2$ in the transition state **TSI$_{syn}$** into the most important MOs of the fragments frozen at their **TSI$_{syn}$** geometry. The molecular orbitals' mixing coefficients (c) are given together with their energy (ε).

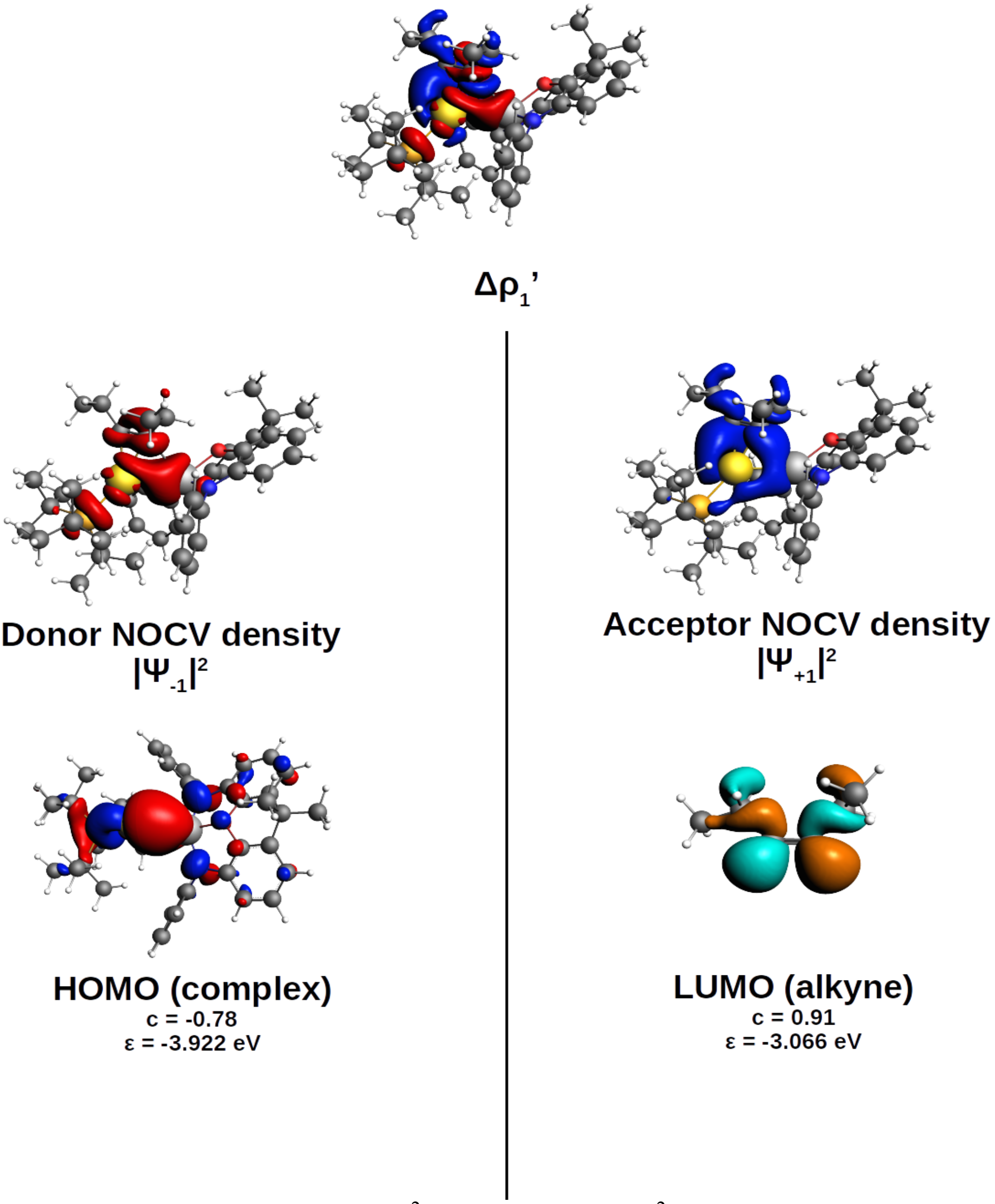

Figure S10. Breakdown of the donor ($|\Psi_1|^2$) and acceptor ($|\Psi_{-1}|^2$) NOCV densities that are associated with the deformation density $\Delta\rho'_1$ in the transition state $\mathbf{TSI_{syn}^{Au}}$ into the most important MOs of the fragments frozen at their $\mathbf{TSI_{syn}^{Au}}$ geometry. The molecular orbitals' mixing coefficients (c) are given together with their energy (ε).

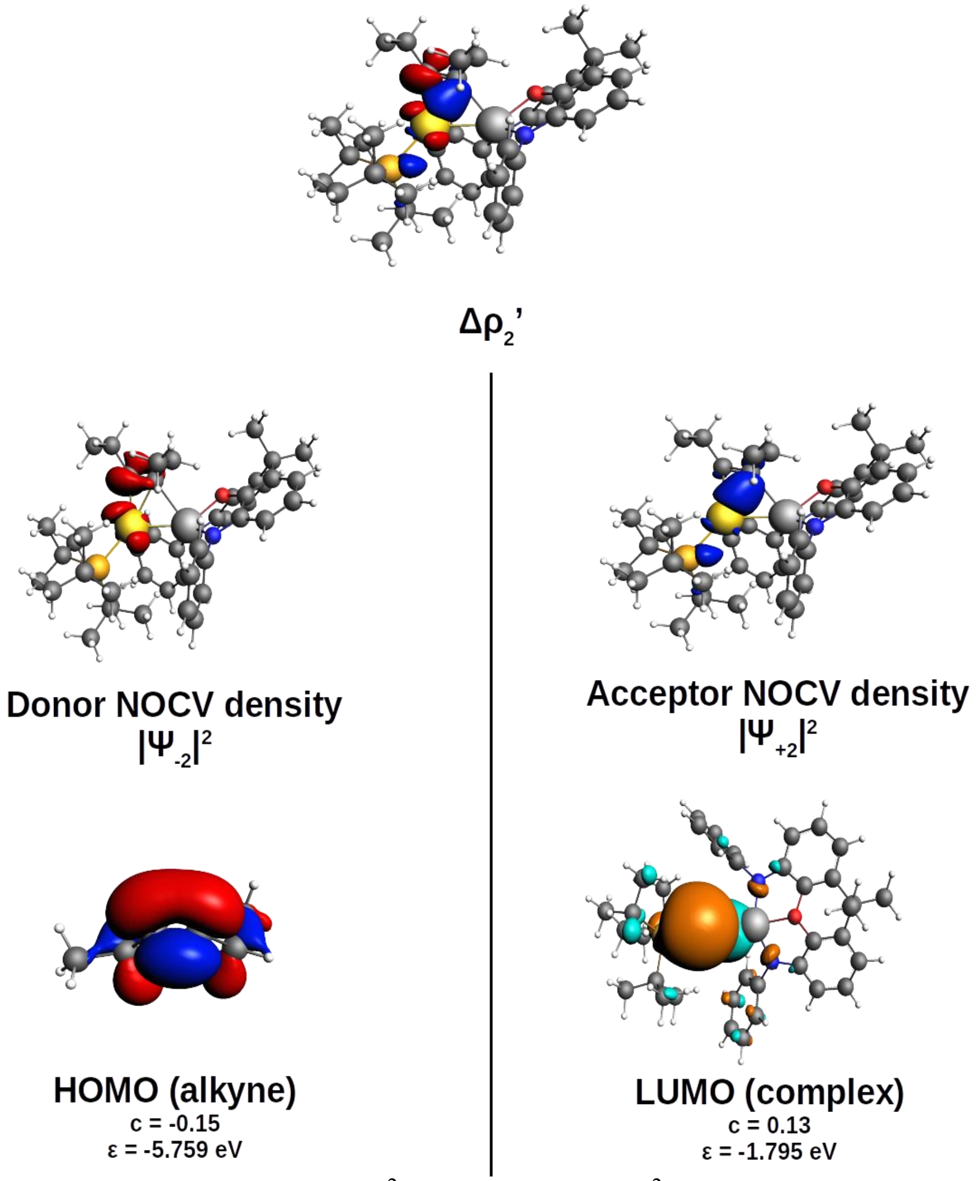

Figure S11. Breakdown of the donor ($|\Psi_2|^2$) and acceptor ($|\Psi_{-2}|^2$) NOCV densities that are associated with the deformation density $\Delta\rho'_2$ in the transition state $\mathbf{TSI}_{syn}^{Au}$ into the most important MOs of the fragments frozen at their $\mathbf{TSI}_{syn}^{Au}$ geometry. The molecular orbitals' mixing coefficients (c) are given together with their energy (ε).

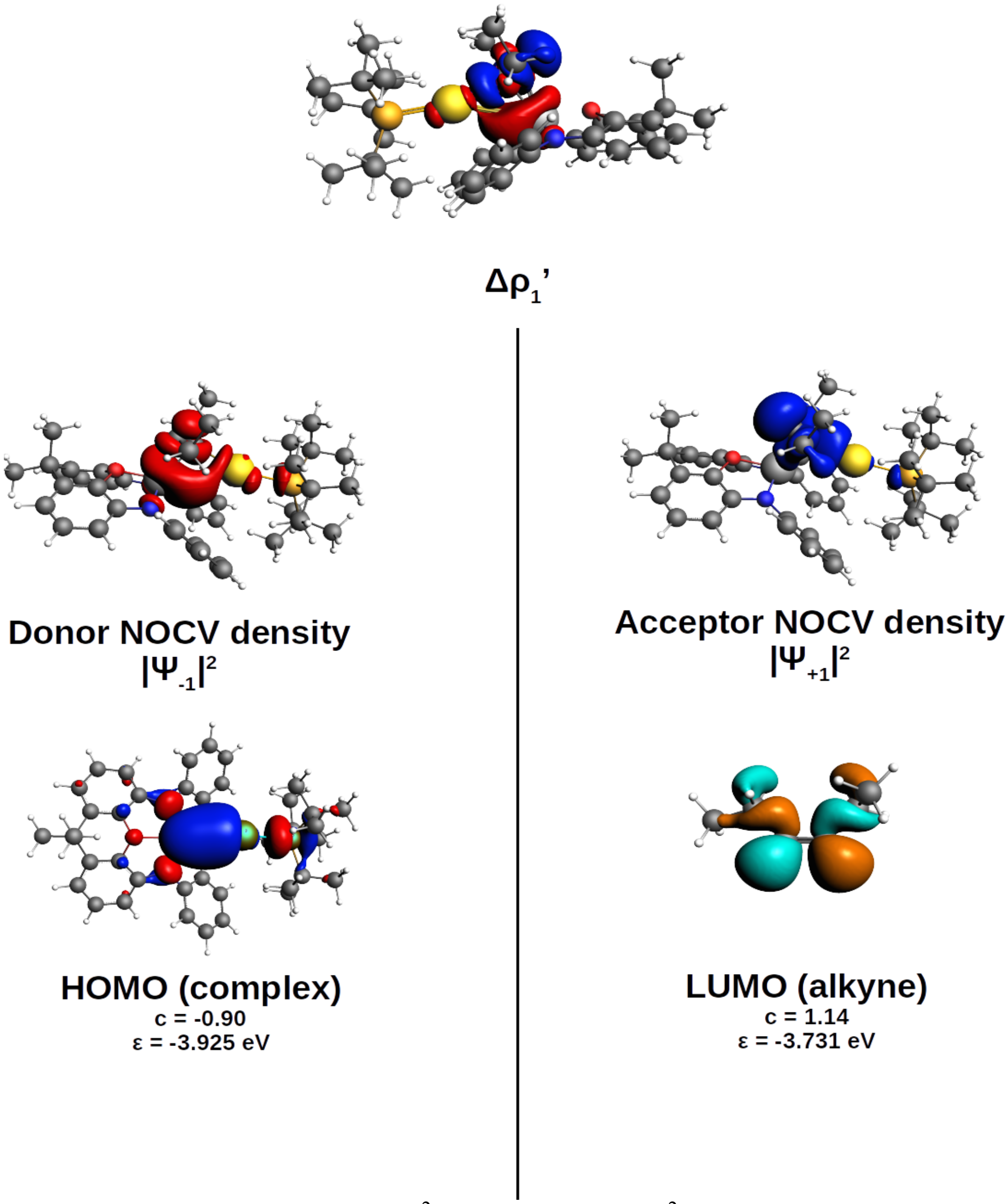

Figure S12. Breakdown of the donor ($|\Psi_1|^2$) and acceptor ($|\Psi_{-1}|^2$) NOCV densities that are associated with the deformation density $\Delta\rho'_1$ in the transition state **TS$_{\mathbf{anti}}$** into the most important MOs of the fragments frozen at their **TS$_{\mathbf{anti}}$** geometry. The molecular orbitals' mixing coefficients (c) are given together with their energy (ε).

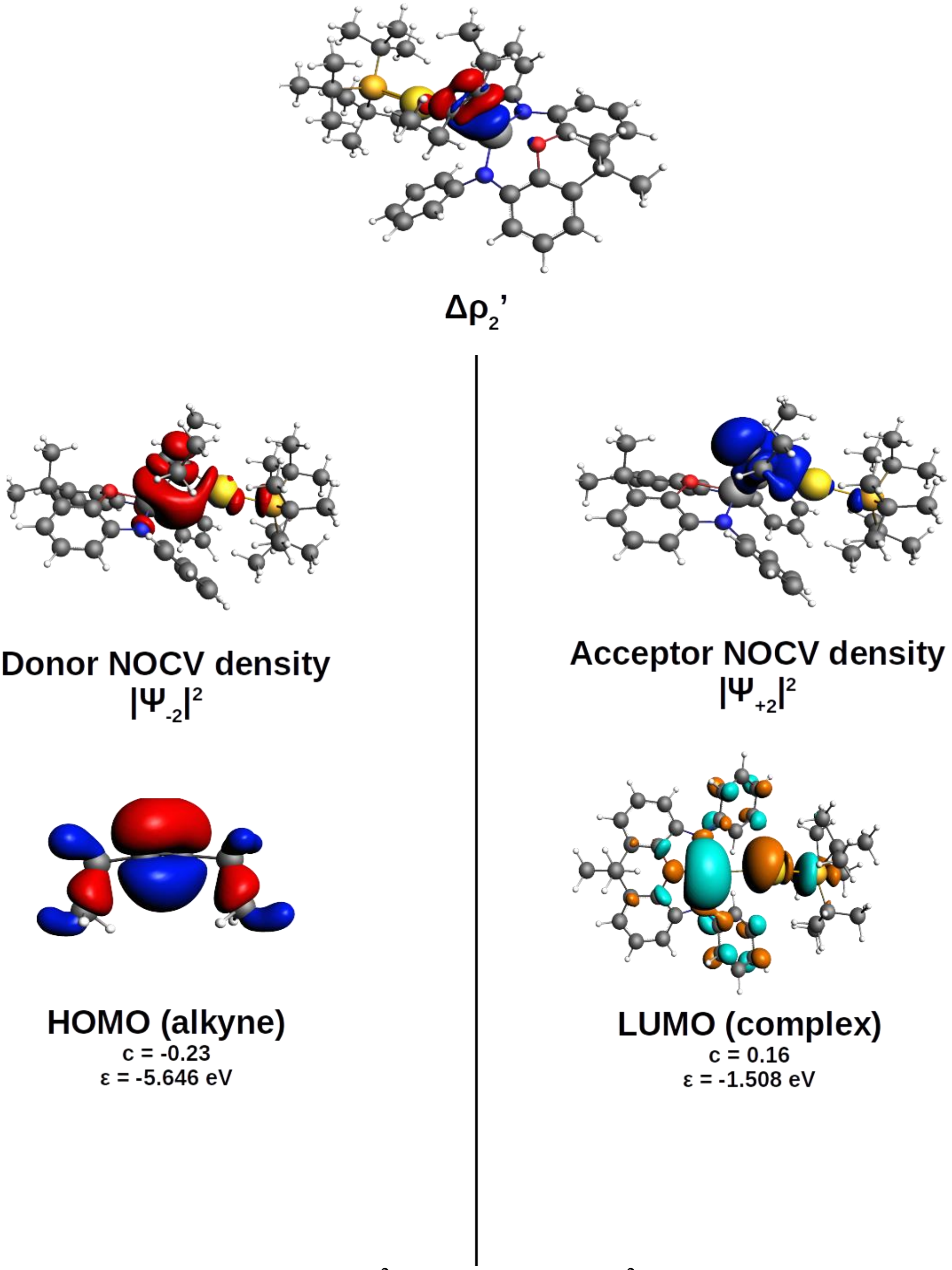

Figure S13. Breakdown of the donor ($|\Psi_2|^2$) and acceptor ($|\Psi_{-2}|^2$) NOCV densities that are associated with the deformation density $\Delta\rho'_2$ in the transition state **TS$_{anti}$** into the most important MOs of the fragments frozen at their **TS$_{anti}$** geometry. The molecular orbitals' mixing coefficients (c) are given together with their energy (ε).

		PC_{syn}	PC_{syn}'	PC_{anti}
**ΔE_{prep}**	**[$^{t}Bu_3PAu$]·**	0.8	0.7	0.5
	**[Al(NON)]·**	1.5	7.4	0.4
	**[EtCCEt]**	72.3	65.1	50.6
	**Sum**	74.7	73.2	51.5
**ΔE_{int}**		-175.7	-177.2	-155.1
**ΔE**		-101.0	-104.0	-103.6
**ΔE_{prep}**	**[$^{t}Bu_3PAu$]$^{+}$**	6.6	6.4	4.9
	**[Al(NON)]$^{-}$**	9.0	16.1	9.0
	**[EtCCEt]**	72.3	65.1	50.6
	**Sum**	88.0	87.6	64.5
**ΔE_{int}**		-217.8	-229.4	-206.0
**ΔE**		-129.8	-141.8	-141.4

Table S6. Results of the analysis of the formation of the **PC_{syn}**, **PC_{syn}'** and **PC_{anti}** products from radical ([$^{t}Bu_3Au$]· and [Al(NON)]·) or closed

References

[1] M. Mitoraj, A. Michalak, *J. Mol. Model.* **2007**, *13*, 347–355.

[2] A. Michalak, M. Mitoraj, T. Ziegler, *J. Phys. Chem. A* **2008**, *112*, 1933–1939.

[3] R. F. Nalewajski, J. ozek, *Int. J. Quantum Chem.* **1994**, *51*, 187–200.

[4] R. F. Nalewajski, J. Mrozek, A. Michalak, *Int. J. Quantum Chem.* **1997**, *61*, 589–601.

[5] T. Lu, F. Chen, *J. Phys. Chem. A* **2013**, *117*, 3100–3108.

[6] L. Belpassi, I. Infante, F. Tarantelli, L. Visscher, *J. Am. Chem. Soc.* **2008**, *130*, 1048–1060.

[7] G. Bistoni, S. Rampino, F. Tarantelli, L. Belpassi, *J. Chem. Phys.* **2015**, *142*, 084112.

[8] N. Salvi, L. Belpassi, F. Tarantelli, *Chem. - A Eur. J.* **2010**, *16*, 7231–7240.

[9] G. Bistoni, L. Belpassi, F. Tarantelli, *J. Chem. Theory Comput.* **2016**, *12*, 1236–1244.

[10] F. M. Bickelhaupt, E. J. Baerends, in *Rev. Comput. Chem.*, Wiley-VCH Verlag, **2007**, pp. 1–86.

[11] K. Morokuma, *J. Chem. Phys.* **1971**, *55*, 1236–1244.

[12] L. Zhao, M. von Hopffgarten, D. M. Andrada, G. Frenking, *Wiley Interdiscip. Rev. Comput. Mol. Sci.* **2018**, *8*, 1345.

[13] M. P. Mitoraj, A. Michalak, T. Ziegler, *J. Chem. Theory Comput.* **2009**, *5*, 962–975.

[14] I. Fernández, F. M. Bickelhaupt, *Chem. Soc. Rev.* **2014**, *43*, 4953–4967.

[15] F. M. Bickelhaupt, K. N. Houk, *Angew. Chemie - Int. Ed.* **2017**, *56*, 10070–10086.

[16] P. Vermeeren, S. C. C. van der Lubbe, C. Fonseca Guerra, F. M. Bickelhaupt, T. A. Hamlin, *Nat. Protoc.* **2020**, *15*, 649–667.

[17] D. Sorbelli, L. Belpassi, P. Belanzoni, *J. Am. Chem. Soc.* **2021**, *143*, 14433–14437.

[18] D. Sorbelli, L. Belpassi, P. Belanzoni, *Chem. Sci.* **2022**, DOI 10.1039/D2SC00630H.

[19] G. te Velde, F. M. Bickelhaupt, E. J. Baerends, C. Fonseca Guerra, S. J. A. van Gisbergen, J. G. Snijders, T. Ziegler, *J. Comput. Chem.* **200**1, *22*, 931–967.

[20] *ADF Manual ADF Program System Release 2014*, **1993**.

[21] M. Swart, F. M. Bickelhaupt, *J. Comput. Chem.* **2008**, *29*, 724–734.

[22] J. P. Perdew, K. Burke, M. Ernzerhof, *Phys. Rev. Lett.* **1996**, *77*, 3865–3868.

[23] E. Van Lenthe, E. J. Baerends, J. G. Snijders, *J. Chem. Phys.* **1993**, *99*, 4597–4610.

[24] E. Van Lenthe, E. J. Baerends, J. G. Snijders, *J. Chem. Phys.* **1994**, *101*, 9783–9792.

[25] E. Van Lenthe, *J. Chem. Phys.* **1999**, *110*, 8943–8953.

[26] S. Grimme, J. Antony, S. Ehrlich, H. Krieg, *J. Chem. Phys.* **2010**, *132*, 154104.

[27] S. Grimme, S. Ehrlich, L. Goerigk, *J. Comput. Chem.* **2011**, *32*, 1456–1465.

[28] C. C. Pye, T. Ziegler, *Theor. Chem. Acc.* **1999**, *101*, 396–408.

[29] J. Hicks, A. Mansikkamäki, P. Vasko, J. M. Goicoechea, S. Aldridge, *Nat. Chem.* **2019**, *11*, 237–241.

[30] D. Sorbelli, L. Belpassi, P. Belanzoni, *Inorg. Chem.* **2022**, *61*, 1704–1716.

xyz geometries

Cu-Al structures

110

RC$_{syn}$

C	-0.408224	3.301857	1.700043
C	-1.128481	3.273995	0.488590
C	-0.952786	4.350856	-0.404383
C	-0.109242	5.410562	-0.083462
C	0.596098	5.427824	1.124095
C	0.444253	4.359918	2.009577
N	-1.901885	2.143724	0.202967
AL	-1.110791	0.375426	0.434746
CU	1.174472	0.330887	-0.121401
P	3.355422	0.707576	-0.528656
C	4.251596	-0.810271	-1.281039
C	3.324717	-1.408747	-2.358588
N	-1.831072	-0.951967	1.665231
C	-3.026131	-1.556017	1.298951
C	-3.577824	-1.118959	0.085156
C	-4.818346	-1.442878	-0.434256
C	-5.569386	-2.373051	0.297379
C	-5.053401	-2.882976	1.493922
C	-3.816370	-2.487047	2.005844
C	-5.253052	-0.717994	-1.715406
C	-4.827663	0.745027	-1.533626
C	-3.590807	0.976542	-0.959895
O	-2.803262	-0.135316	-0.574177
C	-5.592843	1.881455	-1.833137
C	-5.093938	3.147957	-1.508915
C	-3.867391	3.329215	-0.866954
C	-3.067348	2.211448	-0.544831
C	-6.757145	-0.845009	-1.957838
C	-4.494195	-1.325750	-2.922503
C	-0.986416	-1.498581	2.638832
C	-0.135188	-0.630340	3.351932
C	0.808766	-1.120624	4.251740
C	0.922440	-2.492241	4.481737
C	0.077466	-3.364962	3.789257
C	-0.859817	-2.882286	2.879882
C	3.505350	2.197436	-1.728375
C	2.396836	3.200575	-1.356717
C	4.157745	1.157485	1.155426
C	3.597752	0.174879	2.203714
C	4.868997	2.899308	-1.740722
C	3.176576	1.718948	-3.152325
C	5.690042	1.143044	1.188623
C	3.654718	2.548008	1.576657
C	5.634111	-0.534009	-1.884940

C	4.388955	-1.881814	-0.186491
H	-3.553992	4.328076	-0.571289
H	-6.574896	1.782965	-2.291420
H	-6.554875	-2.681763	-0.046084
H	-3.482798	-2.864509	2.970104
H	-7.034995	-1.899206	-2.083383
H	-7.039574	-0.320484	-2.879395
H	-7.338381	-0.427083	-1.125858
H	-4.764223	-2.384177	-3.037704
H	-3.407759	-1.258720	-2.789200
H	-4.765367	-0.789785	-3.842108
H	-5.697361	4.027930	-1.736283
H	-5.648775	-3.597095	2.064479
H	3.070936	2.607822	-3.792719
H	3.963640	1.093365	-3.585643
H	2.225705	1.170294	-3.185345
H	2.378360	3.997287	-2.116731
H	1.408658	2.718649	-1.350250
H	2.536020	3.676203	-0.383819
H	4.846543	3.698416	-2.498590
H	5.104836	3.370156	-0.779890
H	5.687771	2.218510	-2.000600
H	3.699007	-2.407060	-2.626848
H	2.301092	-1.535478	-1.980399
H	3.281963	-0.807628	-3.269908
H	4.712596	-2.817489	-0.662636
H	5.132756	-1.617757	0.572430
H	3.430969	-2.087354	0.307888
H	6.058849	-1.485791	-2.241435
H	5.586028	0.142831	-2.745433
H	6.333583	-0.112673	-1.154127
H	3.898810	0.525250	3.203370
H	2.498773	0.143944	2.173893
H	3.965650	-0.847090	2.085190
H	6.023180	1.460106	2.189604
H	6.101875	0.143057	1.011840
H	6.131109	1.831686	0.458817
H	3.962876	2.718378	2.619692
H	4.077549	3.357710	0.973186
H	2.559604	2.613405	1.537227
H	-1.479205	-3.583930	2.323643
H	0.158127	-4.441701	3.947874
H	1.655307	-2.877195	5.190786
H	1.454714	-0.421047	4.783883
H	-0.234070	0.445834	3.200715
H	-0.553234	2.491092	2.416056
H	0.982876	4.354060	2.958214
H	1.254266	6.261336	1.369155
H	0.011785	6.228069	-0.796010
H	-1.458384	4.336358	-1.368820

C	3.451211	-5.991842	-2.009566
C	0.695302	-3.439668	-0.295868
H	3.132312	-6.224625	-3.038663
C	4.885491	-5.439802	-2.034060
H	3.440081	-6.951181	-1.466874
C	-0.627284	-3.404222	-1.074205
H	0.506815	-3.740865	0.746158
H	1.095225	-2.409079	-0.225791
H	-1.342960	-2.741431	-0.572483
H	-0.468801	-3.033820	-2.095523
H	-1.071148	-4.406105	-1.138708
H	5.565594	-6.167427	-2.496123
H	4.936412	-4.507624	-2.611952
H	5.241145	-5.231408	-1.016677
C	1.686837	-4.326532	-0.887357
C	2.482732	-5.085628	-1.401568

110

RC$_{trans}$

C	-2.714904	2.049401	0.875489
C	-3.397910	1.066752	0.141142
C	-4.713415	1.102380	-0.282436
C	-5.414167	2.286056	-0.004279
C	-4.771652	3.324204	0.679890
C	-3.451509	3.224262	1.126589
C	-5.258809	-0.133485	-1.010678
C	-4.659531	-1.366252	-0.320362
C	-3.347059	-1.285478	0.105752
O	-2.633701	-0.090345	-0.074227
C	-5.309761	-2.584760	-0.069041
C	-4.622978	-3.610435	0.590861
C	-3.307395	-3.465867	1.040114
C	-2.623198	-2.253450	0.820065
N	-1.378792	-1.840956	1.270073
AL	-0.761341	-0.057900	0.749429
C	-6.787915	-0.168676	-0.998611
C	-4.761369	-0.095720	-2.479413
N	-1.448717	1.686541	1.308086
C	-0.497862	2.629610	1.715602
C	0.469728	2.237518	2.663884
C	1.516780	3.086570	3.017820
C	1.625521	4.354177	2.443841
C	0.671988	4.755553	1.503143
C	-0.372199	3.909297	1.137979
C	-0.394711	-2.749614	1.678328
C	-0.189632	-3.999904	1.061717
C	0.880348	-4.809640	1.434342
C	1.779357	-4.399222	2.424017
C	1.591488	-3.159284	3.036530
C	0.519299	-2.346331	2.672938

CU	1.494725	-0.004183	0.139050
P	3.625278	0.031748	-0.523831
C	3.810484	-1.165104	-2.011198
C	2.989655	-2.428912	-1.678807
C	4.097086	1.810848	-1.066566
C	2.873198	2.394039	-1.801207
C	4.782707	-0.529802	0.898299
C	4.245695	0.113091	2.193877
C	4.277396	2.687139	0.183693
C	5.348279	1.924182	-1.945234
C	4.636263	-2.047834	1.093965
C	6.267020	-0.192000	0.714031
C	3.128290	-0.533670	-3.236481
C	5.247527	-1.553644	-2.377373
H	-3.005670	4.036086	1.698246
H	-6.453709	2.397771	-0.306434
H	-6.344318	-2.733265	-0.372444
H	-2.826623	-4.271353	1.592430
H	-7.153708	-1.051714	-1.537466
H	-7.194718	0.713088	-1.509288
H	-7.183391	-0.194532	0.025029
H	-5.096911	-0.997046	-3.010709
H	-3.666402	-0.042794	-2.530564
H	-5.170523	0.787952	-2.988333
H	-5.326470	4.238974	0.893274
H	-5.138718	-4.552474	0.782891
H	2.929129	-3.052584	-2.584306
H	3.426343	-3.036589	-0.882964
H	1.965966	-2.164262	-1.375479
H	3.085342	-1.295334	-4.030465
H	2.097799	-0.224482	-3.012771
H	3.679134	0.323463	-3.637631
H	5.221889	-2.215709	-3.257442
H	5.861900	-0.683271	-2.634825
H	5.748940	-2.102653	-1.572557
H	3.071026	3.455304	-2.018574
H	2.648311	1.892721	-2.745543
H	1.979226	2.339162	-1.163588
H	4.370275	3.732717	-0.148157
H	3.405304	2.632483	0.848141
H	5.180151	2.443110	0.753107
H	5.536803	2.989000	-2.155264
H	6.240432	1.520397	-1.452601
H	5.229734	1.418631	-2.910096
H	5.156490	-2.319657	2.025515
H	3.586054	-2.346750	1.204931
H	5.091638	-2.628012	0.284601
H	6.829651	-0.590985	1.573057
H	6.685130	-0.642381	-0.193703
H	6.451292	0.887271	0.677653

H	4.807377	-0.301438	3.045538
H	4.354151	1.200066	2.217514
H	3.181422	-0.126084	2.334593
H	0.375477	1.258433	3.135574
H	2.249550	2.753772	3.754541
H	2.441209	5.020789	2.723809
H	0.748828	5.738729	1.036098
H	-1.086073	4.227102	0.378513
H	-0.860251	-4.320187	0.264781
H	1.021236	-5.770058	0.935696
H	2.615201	-5.037463	2.711189
H	2.282174	-2.820212	3.809994
H	0.361947	-1.389346	3.172716
C	-0.920966	-1.760795	-2.648219
C	-0.734846	2.357823	-2.557524
C	-0.396274	-2.448882	-3.915706
H	-0.355993	-2.113696	-1.766995
H	-1.967368	-2.058900	-2.468480
C	-2.097302	3.029577	-2.786276
H	-0.358548	2.617737	-1.553181
H	0.004028	2.759149	-3.269896
H	-2.001612	4.119581	-2.692708
H	-2.483203	2.799749	-3.787846
H	-2.831075	2.683272	-2.048082
H	-0.477181	-3.539495	-3.817846
H	-0.971896	-2.135673	-4.796272
H	0.656749	-2.194440	-4.088921
C	-0.841967	-0.306660	-2.685849
C	-0.775487	0.905436	-2.662571

110

TSI$_{syn}$

C	0.125498	-3.280233	-1.354040
C	-0.338112	-2.903853	-0.083562
C	0.152708	-3.591880	1.038508
C	1.059291	-4.640384	0.890792
C	1.503429	-5.016730	-0.379483
C	1.038175	-4.325889	-1.499363
N	-1.297841	-1.871919	0.044357
AL	-0.865523	0.008552	-0.405209
CU	1.547249	0.054405	-0.184221
P	3.671951	0.043155	0.584194
C	4.519332	1.750197	0.373117
C	3.865271	2.743349	1.345597
C	-2.544468	-2.234416	0.509764
C	-3.482802	-1.187197	0.575570
C	-4.788636	-1.261168	1.015556
C	-5.223322	-2.537356	1.424117
C	-4.349495	-3.624492	1.348907
C	-3.030560	-3.499706	0.897795

O	-2.925919	-0.002747	0.122172
C	-3.508778	1.185538	0.527706
C	-4.819438	1.246947	0.958198
C	-5.683868	-0.019223	0.936063
C	-5.287209	2.527606	1.309338
C	-4.438806	3.631125	1.193219
C	-3.113241	3.517640	0.760691
C	-2.592052	2.249247	0.432184
N	-1.330517	1.897300	-0.007374
C	-0.364121	2.922756	-0.097156
C	0.265093	3.190781	-1.324206
C	1.194645	4.226101	-1.438595
C	1.515595	5.014006	-0.333428
C	0.911173	4.742020	0.897445
C	-0.010117	3.704160	1.017575
C	-6.705566	-0.005615	2.081557
C	-6.436622	-0.060839	-0.418248
C	3.540571	-0.372772	2.454689
C	3.184759	-1.861031	2.601624
C	4.739829	-1.290491	-0.292387
C	5.112154	-0.788750	-1.697746
C	4.787194	-0.068721	3.294393
C	2.328175	0.404446	3.009725
C	6.019576	-1.707828	0.443653
C	3.841909	-2.519864	-0.510723
C	6.038759	1.769059	0.581333
C	4.176072	2.258790	-1.040654
H	-2.485758	4.403650	0.685850
H	-6.310339	2.666352	1.653862
H	-6.241008	-2.685710	1.780561
H	-2.381202	-4.372279	0.853971
H	-7.341782	-0.898223	2.043610
H	-7.367331	0.865233	2.000668
H	-6.204005	0.025382	3.057274
H	-7.056443	-0.965979	-0.479705
H	-5.726842	-0.069786	-1.256167
H	-7.082325	0.822206	-0.520916
H	-4.821140	4.621080	1.447385
H	-4.706130	-4.610712	1.650719
H	4.371202	-3.231533	-1.163090
H	3.585071	-3.044504	0.411203
H	2.898893	-2.246428	-1.003159
H	5.557766	-1.629955	-2.250239
H	4.226832	-0.456792	-2.256324
H	5.846995	0.022451	-1.685031
H	6.553808	-2.447198	-0.174131
H	6.699522	-0.866420	0.616839
H	5.807057	-2.183640	1.407434
H	2.917009	-2.043505	3.653700
H	2.319108	-2.135232	1.984552

H	4.019933	-2.525562	2.356348
H	2.136672	0.058762	4.037450
H	2.481832	1.485798	3.041900
H	1.427766	0.207256	2.406461
H	4.598501	-0.388458	4.331483
H	5.672555	-0.607377	2.937693
H	5.023033	1.000710	3.322360
H	6.391813	2.808347	0.487326
H	6.329000	1.408940	1.574893
H	6.572905	1.176149	-0.169494
H	4.525599	3.299026	-1.129653
H	4.646440	1.678675	-1.838273
H	3.089412	2.261501	-1.198804
H	4.212206	3.754813	1.085397
H	2.772302	2.742096	1.252411
H	4.139078	2.559297	2.389551
H	-0.474899	3.488680	1.980084
H	1.168682	5.337267	1.774941
H	2.236357	5.826929	-0.425589
H	1.663830	4.423329	-2.403814
H	-0.016592	2.596181	-2.192381
H	-0.262921	-2.752599	-2.224646
H	1.381310	-4.607716	-2.496153
H	2.212593	-5.837032	-0.493423
H	1.426045	-5.164457	1.774832
H	-0.196546	-3.297494	2.028999
H	-3.313703	-0.900268	-2.590540
H	-3.298684	0.849606	-2.714151
H	1.773333	0.768969	-2.809883
H	1.716312	-0.989479	-2.766685
H	-3.914115	-0.186732	-4.912764
H	-2.367628	0.684522	-5.070385
H	-2.377664	-1.088264	-4.940342
H	2.075005	-0.176342	-5.113737
H	0.508663	-1.017653	-4.998140
H	0.558798	0.757283	-5.043347
C	-0.151104	-0.047374	-2.490400
C	1.180102	-0.100588	-3.136535
C	1.072781	-0.136352	-4.666544
C	-1.401872	-0.035555	-2.550932
C	-2.778123	-0.058612	-3.059757
C	-2.863759	-0.168602	-4.589968

110

TSI_{anti}

C	-0.127981	-2.131568	-2.083003
C	0.792306	-2.620423	-1.140902
C	0.683708	-3.966139	-0.742706
C	-0.302975	-4.787668	-1.281802
C	-1.206887	-4.292872	-2.226728

C	-1.112853	-2.958198	-2.621448
N	1.743153	-1.738997	-0.595427
AL	1.311689	-0.047482	0.180990
N	1.698380	1.616301	-0.656844
C	0.753275	2.475824	-1.253996
C	-0.090404	1.979268	-2.260096
C	-1.045192	2.797378	-2.863447
C	-1.183518	4.128478	-2.470608
C	-0.358831	4.628842	-1.458859
C	0.596827	3.815997	-0.855615
C	3.070973	-2.132338	-0.436762
C	3.907186	-1.200229	0.193492
C	5.283545	-1.236791	0.302423
C	5.902136	-2.399416	-0.180271
C	5.116179	-3.402000	-0.759627
C	3.730865	-3.286299	-0.907521
O	3.230592	-0.030604	0.603965
C	3.874875	1.146612	0.163640
C	5.250574	1.220861	0.269612
C	5.973473	0.009335	0.878876
C	5.841360	2.383061	-0.246589
C	5.032433	3.349688	-0.854370
C	3.650926	3.194889	-0.999425
C	3.018715	2.038644	-0.497962
C	7.467770	0.024301	0.557403
C	5.782439	0.028752	2.417138
CU	-1.794961	-0.131188	0.590434
P	-3.994280	0.021967	0.172258
C	-4.532216	-0.440158	-1.611085
C	-5.940295	0.019586	-2.011239
C	-4.830715	-1.188116	1.407458
C	-6.322169	-1.455338	1.166686
C	-4.558613	1.818411	0.551747
C	-6.056240	1.998455	0.827181
C	-4.053586	-2.520112	1.361663
C	-4.646008	-0.632362	2.830013
C	-3.748694	2.318389	1.762659
C	-4.137926	2.717654	-0.621915
C	-3.489818	0.166537	-2.567951
C	-4.443530	-1.963497	-1.790684
H	3.074572	3.945520	-1.535995
H	6.918869	2.526986	-0.197043
H	6.982690	-2.516816	-0.126421
H	3.173971	-4.066161	-1.422415
H	7.961875	-0.847349	1.004713
H	7.939438	0.920214	0.980223
H	7.649039	0.011640	-0.525140
H	6.261659	-0.853385	2.862740
H	4.720192	0.023920	2.690912
H	6.245056	0.932072	2.837077

H	5.498480	4.249442	-1.258260
H	5.604025	-4.301463	-1.137563
H	-4.387534	-3.143758	2.204894
H	-4.219420	-3.085989	0.442372
H	-2.971843	-2.357029	1.471188
H	-4.946489	-1.414309	3.543587
H	-3.596894	-0.378917	3.034965
H	-5.267848	0.247495	3.025018
H	-6.690763	-2.120668	1.963129
H	-6.922598	-0.539526	1.196074
H	-6.508703	-1.958898	0.211793
H	-3.972434	3.386632	1.908085
H	-3.985311	1.799959	2.694494
H	-2.669819	2.224437	1.580670
H	-4.292576	3.764348	-0.318820
H	-3.073387	2.601137	-0.863217
H	-4.728816	2.549144	-1.528033
H	-6.256683	3.071787	0.971702
H	-6.684891	1.651541	-0.000082
H	-6.373643	1.484040	1.741420
H	-4.558234	-2.183580	-2.863201
H	-3.469930	-2.357583	-1.475556
H	-5.237051	-2.500331	-1.261423
H	-6.146397	-0.339576	-3.031907
H	-6.715066	-0.390438	-1.353360
H	-6.040136	1.110053	-2.024541
H	-3.694647	-0.198609	-3.586330
H	-3.512604	1.257934	-2.591448
H	-2.474290	-0.144891	-2.288568
H	0.040868	0.950788	-2.596321
H	-1.673691	2.391992	-3.656547
H	-1.926603	4.769828	-2.944698
H	-0.465396	5.663328	-1.129480
H	1.220066	4.210040	-0.054220
H	1.361682	-4.354734	0.016125
H	-0.372782	-5.825118	-0.951551
H	-1.974171	-4.941493	-2.649661
H	-1.801516	-2.556408	-3.364802
H	-0.034898	-1.099181	-2.420519
H	1.088532	2.640533	1.862978
H	-0.503899	2.166109	1.203652
H	-0.313749	-2.572436	1.296718
H	1.137977	-2.071180	2.189827
H	-0.775499	3.532656	3.255386
H	-1.340189	1.872168	3.578901
H	0.239519	2.395576	4.191191
H	-0.537914	-3.118654	3.728017
H	-0.233977	-1.399843	4.133689
H	-1.725829	-1.864061	3.294983
C	0.209169	2.003896	2.047180

C	-0.146576	-0.402669	1.587975
C	0.063662	-1.861111	2.044495
C	-0.651307	-2.081592	3.380475
C	0.599975	0.563855	2.237764
C	-0.457310	2.484030	3.347548

110

TSI_{syn}^{Cu}

C	0.553792	-4.087626	-0.490301
C	0.741482	-2.872761	-1.170166
C	0.152210	-2.711302	-2.431002
C	-0.618779	-3.729718	-2.993107
C	-0.800569	-4.932658	-2.310948
C	-0.208437	-5.106452	-1.057009
N	1.509967	-1.820593	-0.613637
C	2.858866	-2.062264	-0.389887
C	3.586007	-0.982939	0.127965
C	4.955292	-0.887839	0.282831
C	5.683807	-2.048376	-0.024008
C	5.006790	-3.185358	-0.479906
C	3.623158	-3.210458	-0.678231
C	5.526368	0.459189	0.748750
C	4.701124	1.563413	0.071840
C	3.347699	1.344502	-0.081544
O	2.778658	0.141308	0.362625
C	2.415070	2.162285	-0.729111
C	2.918035	3.404757	-1.165287
C	4.275944	3.695999	-0.994518
C	5.174172	2.796556	-0.408951
C	7.013833	0.583784	0.416140
C	5.335764	0.572135	2.283170
AL	0.806193	-0.100269	-0.081686
CU	-1.354831	0.125509	0.925610
P	-3.343598	-0.104637	0.058495
C	-3.602701	0.164940	-1.828501
C	-3.634458	1.670157	-2.123175
N	1.136524	1.637923	-0.857178
C	0.160903	2.561752	-1.332952
C	-0.030802	2.758622	-2.706666
C	-0.902233	3.748081	-3.162757
C	-1.594812	4.548499	-2.251590
C	-1.429328	4.337390	-0.881115
C	-0.559112	3.347510	-0.422852
C	-3.780994	-1.934615	0.482430
C	-3.088894	-2.864111	-0.525709
C	-4.570418	1.058373	0.992982
C	-4.644545	0.614672	2.463149
C	-3.158056	-2.269429	1.852020
C	-5.280667	-2.258029	0.513416
C	-3.946602	2.465958	1.010611

C	-5.993269	1.126527	0.422200
C	-2.372447	-0.381696	-2.566699
C	-4.869965	-0.484773	-2.402591
H	2.258441	4.119468	-1.655013
H	6.228260	3.055383	-0.330030
H	6.767173	-2.065903	0.077169
H	3.143794	-4.098943	-1.085111
H	7.587069	-0.202034	0.923867
H	7.404274	1.547518	0.766100
H	7.192157	0.505169	-0.663992
H	5.888342	-0.230694	2.789899
H	4.277010	0.491754	2.559725
H	5.712228	1.541444	2.637147
H	4.649963	4.657750	-1.348771
H	5.581785	-4.082084	-0.715655
H	-5.183776	1.391516	3.026296
H	-5.186750	-0.326604	2.600852
H	-3.638511	0.521725	2.891185
H	-4.545525	3.101027	1.682031
H	-2.920745	2.421820	1.402219
H	-3.923639	2.943725	0.028709
H	-6.595840	1.779005	1.073637
H	-6.024123	1.558788	-0.583556
H	-6.483907	0.147516	0.393029
H	-3.350844	-3.332807	2.064274
H	-2.068962	-2.123237	1.834680
H	-3.568862	-1.679937	2.675462
H	-3.195460	-3.898354	-0.165328
H	-3.530032	-2.821291	-1.526230
H	-2.016627	-2.649036	-0.600704
H	-5.395544	-3.336058	0.707943
H	-5.809495	-1.726867	1.312044
H	-5.779832	-2.039676	-0.436527
H	-2.491257	-0.168990	-3.640702
H	-1.459438	0.117770	-2.216909
H	-2.235030	-1.457220	-2.445070
H	-4.950718	-0.200306	-3.463411
H	-4.836161	-1.578719	-2.362586
H	-5.783260	-0.148663	-1.900780
H	-3.571779	1.804782	-3.213430
H	-4.554878	2.156359	-1.784824
H	-2.775335	2.184688	-1.681245
H	1.012975	-4.223991	0.488767
H	-0.347810	-6.041327	-0.512383
H	-1.399458	-5.730184	-2.751177
H	-1.072559	-3.582067	-3.974016
H	0.315338	-1.776985	-2.968945
H	0.530727	2.141618	-3.409174
H	-1.037293	3.897854	-4.234758
H	-2.270087	5.326562	-2.608784

H	-1.979549	4.947250	-0.163586
H	-0.413960	3.175643	0.644907
H	-0.253242	0.855157	4.790843
H	-1.922407	0.350431	4.502050
H	2.187196	-0.819859	2.301062
H	1.433746	-0.358586	3.818149
H	-1.828295	2.687612	5.396580
H	-0.854264	3.191434	3.994584
H	-2.529895	2.663896	3.756934
H	1.529822	-2.880840	3.456059
H	-0.142133	-2.287922	3.659815
H	0.466748	-2.682998	2.040460
C	0.163621	0.043608	2.172000
C	1.228651	-0.810816	2.832710
C	0.744243	-2.252918	3.012370
C	-0.842103	0.699544	2.722768
C	-1.134083	1.036602	4.143945
C	-1.617117	2.479175	4.338212

110

PC$_{syn}$

C	-5.436865	1.385828	1.351169
C	-4.560098	1.136789	0.280329
C	-4.638569	1.963118	-0.854350
C	-5.537540	3.027080	-0.905120
C	-6.398064	3.272172	0.166434
C	-6.344520	2.440390	1.288586
N	-3.556893	0.149260	0.321627
AL	-1.702675	0.515797	-0.000295
C	-3.841711	-1.151559	0.702587
C	-2.765228	-2.051642	0.641967
C	-2.836356	-3.431322	0.684981
C	-4.086869	-3.977401	1.008683
C	-5.176874	-3.120903	1.197234
C	-5.083283	-1.737874	1.030991
O	-1.536540	-1.451779	0.236521
C	-1.043824	-2.083381	-0.939252
C	-1.055032	-3.464724	-0.984848
C	-1.586050	-4.208440	0.252236
C	-0.643624	-4.041776	-2.195049
C	-0.301432	-3.212258	-3.268233
C	-0.348687	-1.818910	-3.187418
C	-0.726706	-1.195624	-1.980417
N	-0.893746	0.153017	-1.686665
C	-0.372560	1.187486	-2.479226
C	0.797171	1.071535	-3.253418
C	1.298361	2.165022	-3.956933
C	0.659896	3.405225	-3.905975
C	-0.494729	3.536244	-3.130827
C	-1.005561	2.444846	-2.436353

C	-1.909613	-5.668809	-0.064134
C	-0.530605	-4.162929	1.383381
CU	1.828233	1.119019	0.761401
P	3.776819	0.049807	0.522651
C	3.528253	-1.752292	-0.082515
C	2.480395	-1.703554	-1.206612
C	4.908185	0.976535	-0.722327
C	4.715484	2.486605	-0.480133
C	4.656447	0.000030	2.237839
C	3.575173	-0.275731	3.302579
C	6.397891	0.617062	-0.658733
C	4.397227	0.715503	-2.148061
C	5.781314	-1.035710	2.367386
C	5.237510	1.385808	2.567009
C	4.779306	-2.485534	-0.580312
C	2.883707	-2.556240	1.058162
H	-0.125438	-1.218937	-4.066250
H	-0.623136	-5.123179	-2.314388
H	-4.220289	-5.055019	1.078245
H	-5.977264	-1.121681	1.097008
H	-2.290346	-6.176020	0.831269
H	-1.002055	-6.198889	-0.380122
H	-2.660084	-5.754961	-0.860209
H	-0.926758	-4.658566	2.279666
H	-0.265161	-3.132935	1.646589
H	0.381270	-4.686153	1.066451
H	-0.007790	-3.668053	-4.214727
H	-6.149704	-3.550228	1.440940
H	5.273439	3.035447	-1.254435
H	3.654293	2.761537	-0.565648
H	5.079516	2.822063	0.494307
H	4.917929	1.408065	-2.827084
H	4.605028	-0.302060	-2.494971
H	3.321914	0.915008	-2.232692
H	6.925716	1.174923	-1.448204
H	6.860020	0.891227	0.295483
H	6.576563	-0.449997	-0.832404
H	2.558161	-3.524415	0.650211
H	3.572198	-2.760927	1.883765
H	1.994975	-2.046451	1.454306
H	2.097341	-2.718331	-1.389499
H	1.630437	-1.071884	-0.919343
H	2.880749	-1.325261	-2.149962
H	4.492081	-3.510322	-0.864254
H	5.208385	-2.011653	-1.470371
H	5.561637	-2.559950	0.182297
H	6.233138	-0.933499	3.366675
H	5.418999	-2.065595	2.280231
H	6.576345	-0.885253	1.628223
H	5.580385	1.367120	3.612869

H	6.100080	1.646411	1.945748
H	4.485447	2.177021	2.480297
H	4.027286	-0.150270	4.298553
H	2.739928	0.433242	3.212184
H	3.164436	-1.287221	3.248258
H	-1.908889	2.570129	-1.835888
H	-1.008156	4.496604	-3.069100
H	1.056869	4.256879	-4.458379
H	2.211850	2.045798	-4.541799
H	1.335236	0.127157	-3.277754
H	-5.373058	0.774902	2.250546
H	-7.006763	2.625522	2.135505
H	-7.105338	4.100672	0.128278
H	-5.573264	3.658997	-1.793408
H	-3.991864	1.748346	-1.707049
H	0.418121	4.320117	1.540228
H	0.101590	3.475130	3.045331
H	2.362372	4.503460	3.055823
H	2.486417	2.736409	3.169155
H	2.825713	3.549954	1.627349
H	-1.710480	3.858266	1.890184
H	-2.958889	2.914286	1.104827
C	-2.526781	2.265480	3.107681
H	-3.323465	2.876598	3.556878
H	-2.912015	1.242600	2.987289
H	-1.683707	2.218230	3.811287
C	0.330659	2.188461	1.301273
C	0.707689	3.427603	2.125024
C	2.175785	3.563393	2.514918
C	-1.002985	1.950641	1.103737
C	-2.074545	2.828878	1.753407

110

PC_{syn}'

C	1.650801	-2.464292	-2.422611
C	0.660262	-1.554638	-2.818299
C	0.762632	-0.968392	-4.088207
C	1.830664	-1.272524	-4.930292
C	2.816237	-2.175545	-4.524005
C	2.716885	-2.773608	-3.266675
N	-0.416020	-1.190142	-1.969927
C	-1.283883	-2.172895	-1.537228
C	-2.419575	-1.696413	-0.858569
C	-3.427775	-2.453208	-0.294470
C	-3.304236	-3.846560	-0.442948
C	-2.223602	-4.375585	-1.152981
C	-1.222631	-3.570545	-1.703868
C	-4.617618	-1.738456	0.353878
C	-4.133054	-0.406033	0.930817
C	-3.097640	0.256972	0.299582

O	-2.426819	-0.300307	-0.790726
C	-4.690410	0.248964	2.042531
C	-4.189968	1.492388	2.442637
C	-3.124763	2.113164	1.787628
C	-2.524125	1.478271	0.683216
N	-1.400833	1.857627	-0.025493
AL	-0.608790	0.650950	-1.346137
CU	1.564220	0.290608	0.008167
P	1.999676	-0.634926	1.994640
C	2.671759	-2.421369	1.813065
C	3.363274	-2.994551	3.056699
C	-5.656859	-1.435229	-0.756242
C	-5.272001	-2.608460	1.431981
C	-0.983004	3.207659	0.097364
C	-1.859191	4.257780	-0.225782
C	-1.440584	5.584410	-0.145832
C	-0.135820	5.892579	0.251126
C	0.740029	4.855222	0.575644
C	0.317175	3.528922	0.504976
C	0.534776	1.727556	-2.438419
C	1.550069	1.401844	-1.599146
C	3.334004	0.455227	2.846652
C	3.449916	0.262621	4.364328
C	0.405880	-0.653374	3.063221
C	0.476176	-1.490710	4.346048
C	4.702769	0.186644	2.199476
C	3.012839	1.928896	2.534762
C	0.046488	0.800088	3.415645
C	-0.742937	-1.170576	2.178489
C	1.498673	-3.339705	1.429479
C	3.654577	-2.437093	0.625850
H	-0.398439	-4.020389	-2.253632
H	-4.054998	-4.514101	-0.024870
H	-5.513090	-0.203502	2.592662
H	-2.739107	3.068974	2.138641
H	-6.518203	-0.901433	-0.331508
H	-6.005988	-2.371832	-1.212324
H	-5.216660	-0.810303	-1.543976
H	-6.140892	-2.097549	1.864339
H	-4.565581	-2.842574	2.238857
H	-5.634836	-3.549161	1.000742
H	-2.155442	-5.456611	-1.284377
H	-4.639016	1.991540	3.302743
H	3.878276	-3.485368	0.375426
H	3.219903	-1.965101	-0.264860
H	4.602857	-1.941694	0.846602
H	1.912363	-4.303386	1.096359
H	0.834314	-3.543742	2.275553
H	0.897806	-2.930537	0.607042
H	3.654162	-4.035198	2.844451

H	4.277326	-2.449057	3.316238
H	2.706901	-3.006745	3.933701
H	-0.955908	0.800583	3.868270
H	0.736891	1.249072	4.137206
H	-0.005570	1.432047	2.519420
H	-1.699267	-0.932566	2.665567
H	-0.733326	-0.663724	1.204886
H	-0.713767	-2.247398	2.001300
H	-0.479473	-1.380904	4.881615
H	0.609098	-2.558294	4.136617
H	1.274532	-1.167392	5.022279
H	4.282291	0.882266	4.732636
H	2.546380	0.584260	4.893786
H	3.661793	-0.776272	4.640542
H	5.404695	0.950644	2.566392
H	5.117439	-0.791670	2.461942
H	4.660735	0.275031	1.105452
H	3.798363	2.556366	2.983273
H	3.022389	2.108246	1.450436
H	2.050632	2.264543	2.928188
H	-0.007504	-0.264432	-4.400961
H	1.892635	-0.801194	-5.912240
H	3.652842	-2.411963	-5.182107
H	3.480888	-3.477908	-2.933449
H	1.582551	-2.925744	-1.439952
H	0.998390	2.715252	0.744949
H	1.761216	5.078064	0.889614
H	0.193173	6.930807	0.305221
H	-2.134727	6.384036	-0.408812
H	-2.872258	4.019196	-0.551841
C	0.577908	2.649655	-3.619953
C	2.982657	1.905768	-1.745014
C	3.958755	0.786636	-2.130853
H	3.022619	2.701310	-2.507669
H	3.322441	2.371998	-0.802629
H	0.845875	2.065282	-4.519502
C	-0.758326	3.356363	-3.862362
H	1.374746	3.405739	-3.509765
H	4.982428	1.169715	-2.252479
H	3.978906	-0.001391	-1.362490
H	3.647442	0.306544	-3.067932
H	-0.717270	4.015672	-4.741416
H	-1.559638	2.619270	-4.022242
H	-1.037503	3.961790	-2.988850

110

PC$_{anti}$

C	4.237249	1.525485	0.682312
C	4.333905	0.171179	1.034928
C	5.427438	-0.482477	1.568874

C	6.519148	0.331352	1.907159
C	6.453474	1.706863	1.655860
C	5.351296	2.310201	1.044322
C	5.348459	-2.012771	1.675959
C	4.674464	-2.501347	0.384794
C	3.610592	-1.760351	-0.094270
O	3.219710	-0.599366	0.619776
C	5.044620	-3.608478	-0.392382
C	4.354222	-3.877621	-1.580636
C	3.321975	-3.063262	-2.052065
C	2.930828	-1.930569	-1.309632
N	2.034362	-0.923475	-1.644451
AL	1.853278	0.467365	-0.346491
CU	-2.635882	-0.119467	0.852371
P	-4.826525	-0.138390	0.661648
C	-5.630356	-1.209240	2.037735
C	-4.719957	-2.433785	2.260822
C	6.731136	-2.637397	1.862618
C	4.453639	-2.392118	2.883625
N	3.110024	1.873692	-0.050558
C	2.811144	3.215606	-0.350018
C	2.349157	3.532323	-1.638607
C	1.939387	4.826630	-1.954752
C	2.005918	5.839156	-0.997335
C	2.481660	5.538016	0.283169
C	2.876371	4.243209	0.609016
C	1.012608	-1.119687	-2.590129
C	0.318363	-2.336092	-2.729593
C	-0.762619	-2.436698	-3.601405
C	-1.184014	-1.334161	-4.350594
C	-0.501326	-0.124256	-4.219560
C	0.589369	-0.022285	-3.359294
C	-5.536723	1.642937	0.741637
C	-4.808227	2.378988	1.884385
C	-5.217283	-0.906813	-1.055753
C	-4.201030	-0.338158	-2.067175
C	-5.146519	2.388002	-0.546019
C	-7.054665	1.742125	0.938553
C	-4.934907	-2.417391	-0.994633
C	-6.645320	-0.681496	-1.566948
C	-5.598170	-0.421452	3.358078
C	-7.067296	-1.670812	1.765157
H	5.370966	3.372517	0.809035
H	7.418644	-0.100353	2.341487
H	5.871302	-4.247101	-0.087346
H	2.848100	-3.277076	-3.008411
H	6.648176	-3.728894	1.938419
H	7.188777	-2.278820	2.793132
H	7.400674	-2.394559	1.027508
H	4.362465	-3.484645	2.950710

H	3.447127	-1.967099	2.788207
H	4.902701	-2.016843	3.812973
H	7.307904	2.331977	1.918993
H	4.650799	-4.740708	-2.178245
H	-5.084400	-2.980807	3.144130
H	-4.710355	-3.129508	1.418562
H	-3.684160	-2.120337	2.456108
H	-5.886559	-1.106357	4.169875
H	-4.589535	-0.047382	3.579861
H	-6.300562	0.418207	3.372181
H	-7.429481	-2.230340	2.642016
H	-7.752256	-0.831084	1.601554
H	-7.132954	-2.342325	0.901878
H	-5.092851	3.441896	1.847147
H	-5.060248	2.001145	2.878062
H	-3.717578	2.314606	1.759241
H	-5.396723	3.451286	-0.411026
H	-4.067864	2.322105	-0.739589
H	-5.689889	2.032480	-1.427426
H	-7.343053	2.805005	0.924406
H	-7.612720	1.236564	0.142374
H	-7.379232	1.330679	1.900940
H	-4.983363	-2.812670	-2.020655
H	-3.927571	-2.621976	-0.606793
H	-5.667708	-2.967515	-0.395459
H	-6.762600	-1.208319	-2.527001
H	-7.404115	-1.071996	-0.879383
H	-6.859587	0.377461	-1.750308
H	-4.331918	-0.869649	-3.022351
H	-4.328787	0.729466	-2.261051
H	-3.167181	-0.504699	-1.731587
H	2.330881	2.748313	-2.398281
H	1.576779	5.045107	-2.959944
H	1.692472	6.853654	-1.243874
H	2.529608	6.318619	1.043818
H	3.207361	4.011015	1.620895
H	0.609802	-3.192552	-2.122873
H	-1.293993	-3.386268	-3.683361
H	-2.035315	-1.417955	-5.026130
H	-0.814416	0.747942	-4.794981
H	1.133831	0.919964	-3.275391
C	-0.171069	-1.642647	0.844823
C	-0.594097	2.184809	0.138093
C	-1.107866	2.514390	-1.267591
H	-1.438615	2.250314	0.849100
H	0.132357	2.956562	0.441194
H	0.875670	-1.726792	0.495037
C	-0.222718	-2.111635	2.303792
H	-0.745363	-2.352414	0.224498
H	0.151317	-3.140210	2.410949

H	0.384824	-1.453675	2.941796
H	-1.254791	-2.073931	2.684231
H	-1.574772	3.510006	-1.301572
H	-0.291943	2.503928	-2.002828
H	-1.843695	1.762521	-1.586913
C	0.016600	0.795572	0.243843
C	-0.739650	-0.251041	0.668931

110

TS_{side}

C	4.755803	1.170464	-2.609991
C	4.478111	1.016929	-1.242691
C	5.202924	1.779712	-0.317138
C	6.152806	2.706405	-0.749423
C	6.413192	2.863067	-2.111317
C	5.716463	2.083188	-3.039014
N	3.463193	0.131185	-0.795414
AL	1.844674	0.643501	0.083174
C	3.664102	-1.227082	-1.007894
C	2.671894	-2.081292	-0.505582
C	2.732426	-3.457351	-0.396299
C	3.863519	-4.068817	-0.960643
C	4.849348	-3.273499	-1.554519
C	4.777629	-1.877596	-1.576575
O	1.598732	-1.385045	0.077832
C	1.231577	-1.898381	1.336634
C	1.190192	-3.265913	1.527077
C	1.587363	-4.163278	0.344558
C	0.799701	-3.693272	2.806718
C	0.507743	-2.740896	3.790776
C	0.642257	-1.367535	3.571381
C	1.063914	-0.900499	2.309364
N	1.405507	0.387619	1.933901
C	0.970853	1.465191	2.741831
C	-0.395848	1.663146	3.000045
C	-0.828979	2.763914	3.733084
C	0.095223	3.693179	4.221775
C	1.455336	3.502635	3.972614
C	1.891742	2.394122	3.244833
C	1.998384	-5.560058	0.815350
C	0.383937	-4.288349	-0.619665
CU	-2.110035	0.962602	-1.052658
P	-3.924247	-0.231457	-0.791828
C	-3.492061	-1.974316	-0.121656
C	-2.311045	-1.812745	0.856152
C	-4.979600	0.709650	0.510881
C	-4.910604	2.215886	0.187489
C	-4.912176	-0.384727	-2.428009
C	-3.901082	-0.684620	-3.552670
C	-6.445117	0.266893	0.604470

C	-4.312442	0.554419	1.887484
C	-6.026271	-1.439281	-2.426856
C	-5.518672	0.987394	-2.766446
C	-4.644518	-2.720762	0.561466
C	-2.966845	-2.829809	-1.285395
H	0.463811	-0.659139	4.379296
H	0.735434	-4.753805	3.042138
H	3.985644	-5.149525	-0.926196
H	5.596031	-1.293244	-1.993486
H	2.268640	-6.188451	-0.042488
H	1.162119	-6.052441	1.327372
H	2.853274	-5.518400	1.502209
H	0.662064	-4.894874	-1.492296
H	0.055535	-3.303759	-0.972395
H	-0.457945	-4.772939	-0.106330
H	0.196010	-3.082833	4.779008
H	5.724230	-3.756606	-1.992143
H	-5.396770	2.765774	1.007986
H	-3.866709	2.556366	0.122940
H	-5.416449	2.488542	-0.741433
H	-4.819596	1.233037	2.589834
H	-4.391363	-0.458499	2.294159
H	-3.253444	0.843631	1.851362
H	-6.930473	0.829409	1.417218
H	-7.002690	0.478640	-0.314779
H	-6.547822	-0.799749	0.832308
H	-2.569636	-3.764915	-0.865853
H	-3.747071	-3.098524	-2.004386
H	-2.146909	-2.330134	-1.818279
H	-1.936700	-2.809973	1.130787
H	-1.481311	-1.262829	0.390291
H	-2.577330	-1.297468	1.781942
H	-4.288303	-3.720125	0.856281
H	-4.987855	-2.217716	1.472034
H	-5.505413	-2.858215	-0.102990
H	-6.546250	-1.402391	-3.396809
H	-5.639854	-2.457115	-2.304031
H	-6.771868	-1.258301	-1.644377
H	-5.919749	0.938022	-3.789975
H	-6.344341	1.264698	-2.103306
H	-4.759464	1.781180	-2.744116
H	-4.428904	-0.621803	-4.516573
H	-3.090239	0.058392	-3.559187
H	-3.453197	-1.678589	-3.482427
H	2.954608	2.237786	3.055844
H	2.185548	4.218746	4.352209
H	-0.243623	4.559143	4.791175
H	-1.895802	2.907627	3.913188
H	-1.110341	0.947809	2.592734
H	4.191304	0.579974	-3.332500

H	5.912036	2.198586	-4.105999
H	7.156120	3.585912	-2.449650
H	6.698295	3.301276	-0.015757
H	5.015958	1.634368	0.748001
H	-1.676874	3.804989	0.154382
H	-0.168632	4.319525	-0.591095
H	-2.135801	5.292080	-1.797032
H	-1.258906	4.176536	-2.873903
H	-2.769654	3.653511	-2.107562
H	1.376571	3.440168	0.596332
H	2.888535	2.573324	0.505662
C	2.367898	3.522257	-1.373960
H	3.208298	4.221533	-1.236915
H	2.669669	2.806472	-2.149302
H	1.521137	4.088730	-1.781503
C	-0.704403	2.244873	-0.892003
C	-1.062337	3.699918	-0.756575
C	-1.852677	4.241430	-1.951562
C	0.395967	1.564991	-0.693736
C	1.976018	2.825796	-0.080554

110

PC$_{side}$

C	-4.721094	1.693339	2.001303
C	-3.533805	-0.760042	0.630895
C	-4.208350	1.541017	0.701052
C	-4.649391	2.420948	-0.298915
C	-5.550376	3.443269	-0.001934
C	-6.048157	3.591906	1.294112
C	-5.632130	2.705985	2.292696
N	-3.228029	0.568888	0.399902
AL	-1.428727	1.131801	-0.126631
C	-2.514593	-1.684771	0.346403
C	-2.624136	-3.061722	0.298067
C	-3.867009	-3.600344	0.670167
C	-4.903226	-2.731783	1.032615
C	-4.763341	-1.339827	1.013222
O	-1.333369	-1.075730	-0.061701
C	-0.754406	-1.657414	-1.179343
C	-0.745800	-3.033111	-1.310540
C	-1.383301	-3.839978	-0.167409
C	-0.164197	-3.538808	-2.485101
C	0.339865	-2.645690	-3.438072
C	0.260154	-1.257141	-3.284976
C	-0.328769	-0.711961	-2.124986
N	-0.545447	0.621139	-1.807417
C	-0.514804	1.534949	-2.897983
C	0.387763	2.605099	-2.925110
C	0.374774	3.527018	-3.972430
C	-0.534169	3.389354	-5.022183

C	-1.433948	2.318808	-5.011068
C	-1.425327	1.403799	-3.961021
C	-1.735277	-5.261116	-0.607493
C	-0.381308	-3.907364	1.014077
CU	1.613645	0.375512	1.249784
P	3.700506	0.030438	0.663365
C	3.960166	-1.805339	0.190177
C	2.730276	-2.257563	-0.614920
C	4.073377	1.162565	-0.835724
C	3.419614	2.532602	-0.565362
C	4.843114	0.494939	2.132671
C	4.203219	-0.043905	3.426540
C	5.562877	1.352677	-1.148408
C	3.363673	0.582580	-2.070114
C	6.282830	-0.021765	2.006666
C	4.871131	2.024149	2.286337
C	5.240720	-2.089076	-0.605409
C	3.957591	-2.656124	1.471333
H	0.619809	-0.596395	-4.072880
H	-0.111542	-4.611483	-2.663431
H	-4.033097	-4.676131	0.666404
H	-5.614666	-0.703416	1.249980
H	-2.180230	-5.821060	0.224887
H	-0.831255	-5.800462	-0.918624
H	-2.445266	-5.257945	-1.444579
H	-0.827530	-4.461814	1.851281
H	-0.121165	-2.901317	1.367725
H	0.540595	-4.417472	0.701990
H	0.797413	-3.044052	-4.345176
H	-5.868353	-3.153102	1.319326
H	3.477398	3.128038	-1.488946
H	2.357660	2.423980	-0.300155
H	3.910545	3.102198	0.226590
H	3.426334	1.326308	-2.878131
H	3.830934	-0.337905	-2.433992
H	2.298393	0.392976	-1.883831
H	5.650243	1.975789	-2.051594
H	6.098037	1.869723	-0.344106
H	6.073098	0.404484	-1.350249
H	3.933971	-3.713769	1.170220
H	4.848354	-2.510299	2.090622
H	3.062246	-2.467320	2.079501
H	2.806795	-3.342031	-0.782753
H	1.800866	-2.075393	-0.059825
H	2.631007	-1.780573	-1.591343
H	5.309360	-3.174250	-0.776060
H	5.232215	-1.606554	-1.588949
H	6.147275	-1.777917	-0.074530
H	6.862617	0.350574	2.865132
H	6.339693	-1.115460	2.025935

H	6.775844	0.333651	1.095100
H	5.380893	2.258798	3.232725
H	5.423388	2.523930	1.484516
H	3.858789	2.446617	2.340973
H	4.806987	0.302508	4.279046
H	3.186456	0.352801	3.552087
H	4.154949	-1.134174	3.473514
H	-2.135053	0.575432	-3.944972
H	-2.156330	2.203653	-5.820574
H	-0.545783	4.110474	-5.840068
H	1.081818	4.358317	-3.967269
H	1.097522	2.711455	-2.108327
H	-4.374380	1.020455	2.786476
H	-6.008564	2.815333	3.311145
H	-6.753445	4.390560	1.525492
H	-5.869701	4.125461	-0.791187
H	-4.261373	2.299652	-1.310675
H	1.113441	0.955118	4.537001
H	-0.622645	0.698193	4.652739
H	0.633914	-1.297296	5.519714
H	-0.319207	-1.744610	4.079746
H	1.433651	-1.495935	3.940948
C	-0.269648	0.833505	1.539932
C	0.107648	0.617725	2.710419
C	0.286582	0.347251	4.137624
C	0.522317	-1.139665	4.439540
H	-0.382014	3.394138	-0.470696
H	-2.038208	3.529328	-1.009486
C	-1.757528	3.863761	1.121733
H	-1.574815	4.950308	1.063510
H	-2.811147	3.729210	1.402285
H	-1.153430	3.477705	1.958079
C	-1.418108	3.134937	-0.187215

110

TS$_{syn/anti}$

C	-0.398067	1.044866	3.255287
C	-0.679575	1.682931	2.038363
C	0.145248	2.745934	1.630856
C	1.213315	3.159886	2.422924
C	1.488741	2.515251	3.632576
C	0.681797	1.451892	4.039917
N	-1.745794	1.234606	1.224651
C	-2.730948	2.145540	0.872610
C	-3.631003	1.729861	-0.120239
C	-4.804763	2.350158	-0.501647
C	-5.064983	3.591688	0.101013
C	-4.158583	4.102927	1.037805
C	-3.013488	3.406556	1.436963
C	-5.690999	1.614369	-1.518700

C	-5.636464	0.120795	-1.162646
C	-4.419969	-0.386823	-0.749413
O	-3.313256	0.470736	-0.656290
C	-6.704637	-0.790374	-1.185287
C	-6.488044	-2.110469	-0.773674
C	-5.252022	-2.559479	-0.297615
C	-4.159583	-1.670124	-0.245553
N	-2.903341	-1.856865	0.317986
AL	-1.683438	-0.388634	0.200690
C	-7.125559	2.145141	-1.504788
C	-5.089872	1.810936	-2.933681
C	-2.567583	-3.141014	0.806913
C	-2.541945	-4.262362	-0.039542
C	-2.140845	-5.505541	0.443937
C	-1.750722	-5.653873	1.778480
C	-1.782741	-4.547354	2.628183
C	-2.201246	-3.304822	2.150742
CU	2.512815	-0.162684	-1.034012
P	4.610605	0.010812	-0.411438
C	5.144167	1.826477	-0.742330
C	5.248529	2.053136	-2.259482
C	4.900626	-0.402677	1.439098
C	4.330961	0.737705	2.297748
C	5.650428	-1.175691	-1.506308
C	5.446311	-2.615323	-1.005034
C	4.051825	-1.640338	1.785764
C	6.364493	-0.649836	1.825939
C	5.058367	-1.137964	-2.929868
C	7.152635	-0.871097	-1.561795
C	4.001802	2.739730	-0.253297
C	6.465938	2.248900	-0.088567
H	-2.361801	3.816111	2.207518
H	-5.965569	4.150681	-0.146010
H	-7.696065	-0.476214	-1.506027
H	-5.143778	-3.579413	0.067792
H	-7.736267	1.615166	-2.246602
H	-7.141290	3.209795	-1.769687
H	-7.590461	2.023377	-0.517977
H	-5.690088	1.265677	-3.674799
H	-4.058410	1.440167	-2.982100
H	-5.087986	2.878075	-3.194460
H	-4.365206	5.070807	1.497088
H	-7.321967	-2.813463	-0.796870
H	5.554012	-1.916184	-3.530535
H	3.980075	-1.355420	-2.914420
H	5.206080	-0.181706	-3.437249
H	5.892365	-3.298199	-1.743802
H	5.934902	-2.806163	-0.044217
H	4.380528	-2.867328	-0.920218
H	7.643639	-1.640009	-2.178547

H	7.365415	0.099393	-2.023575
H	7.621234	-0.891940	-0.571768
H	4.332183	0.403798	3.346329
H	4.926213	1.655250	2.246739
H	3.292280	0.972075	2.033303
H	4.141006	-1.823239	2.867693
H	2.992983	-1.463775	1.561927
H	4.365378	-2.549372	1.267753
H	6.414228	-0.813121	2.913719
H	6.777401	-1.543703	1.344929
H	7.012935	0.200552	1.588748
H	6.687055	3.285421	-0.387190
H	6.416955	2.229804	1.005427
H	7.308811	1.625406	-0.408014
H	5.372504	3.132814	-2.432632
H	6.108662	1.547074	-2.709660
H	4.336641	1.735479	-2.781929
H	4.234477	3.773645	-0.551450
H	3.043936	2.459463	-0.714835
H	3.866132	2.725389	0.830250
H	-1.043123	0.228204	3.582165
H	0.886042	0.940792	4.981696
H	2.328675	2.836515	4.249115
H	1.843646	3.984860	2.087311
H	-0.059851	3.233803	0.677465
H	-2.818574	-4.139641	-1.087127
H	-2.117139	-6.362453	-0.230710
H	-1.427621	-6.625809	2.152227
H	-1.490941	-4.651986	3.674063
H	-2.252974	-2.443877	2.818839
H	1.184375	-0.106207	-3.901122
H	-0.448387	0.465326	-3.528417
H	0.986985	2.358805	-4.297858
H	0.519495	2.600980	-2.594162
H	2.151446	2.032894	-2.989137
H	0.945302	-0.467476	1.089254
H	-0.382090	-1.383521	1.806931
H	1.329659	-3.064133	1.200986
H	-0.016109	-3.245363	0.062886
H	1.457390	-2.396062	-0.440604
C	0.753741	-0.068181	-1.791281
C	0.602474	0.513067	-3.200380
C	1.092983	1.960855	-3.278574
C	-0.338267	-0.313108	-1.126816
C	0.190231	-1.230447	0.864579
C	0.765849	-2.554025	0.403559

Au-Al structures

110

RC$_{syn}$

C	-0.118335	3.302433	1.933261
C	-0.959319	3.427626	0.810136
C	-0.725385	4.500903	-0.071555
C	0.295129	5.414816	0.178174
C	1.119429	5.282396	1.299082
C	0.909555	4.212422	2.170055
N	-1.914226	2.428056	0.577480
AL	-1.365235	0.577053	0.642960
AU	0.915453	0.239781	-0.087518
P	3.294226	0.161083	-0.554945
C	3.750006	-1.254230	-1.764254
C	2.678883	-1.282493	-2.873146
N	-2.094785	-0.751577	1.834669
C	-3.369470	-1.212691	1.540304
C	-3.985703	-0.611047	0.432858
C	-5.298086	-0.755600	0.024668
C	-6.066901	-1.679471	0.747282
C	-5.488594	-2.363597	1.822661
C	-4.171940	-2.142289	2.233183
C	-5.772090	0.126060	-1.138858
C	-5.154165	1.512171	-0.906408
C	-3.848345	1.557004	-0.454704
O	-3.167388	0.337307	-0.225939
C	-5.793458	2.751557	-1.057281
C	-5.107344	3.924108	-0.720982
C	-3.808975	3.914074	-0.206287
C	-3.132566	2.688412	-0.032086
C	-7.297551	0.192129	-1.215089
C	-5.223952	-0.459429	-2.465146
C	-1.231880	-1.440571	2.700778
C	-0.341935	-0.694219	3.495060
C	0.609741	-1.323588	4.294860
C	0.692040	-2.715898	4.330799
C	-0.189726	-3.468488	3.548939
C	-1.137829	-2.845002	2.741753
C	3.834017	1.843198	-1.303638
C	3.058554	2.960986	-0.580254
C	4.184574	-0.112060	1.122449
C	3.408134	-1.192974	1.901702
C	5.339096	2.133098	-1.240588
C	3.370509	1.897019	-2.769350
C	5.664745	-0.501839	1.025732
C	4.053422	1.175850	1.954168
C	5.146102	-1.162137	-2.393216
C	3.627856	-2.591470	-1.015936
H	-3.338600	4.845767	0.101439

H	-6.820568	2.805155	-1.412885
H	-7.109075	-1.854985	0.487857
H	-3.779593	-2.655615	3.108934
H	-7.713721	-0.808843	-1.384835
H	-7.609925	0.822914	-2.056845
H	-7.731577	0.599596	-0.293025
H	-5.634850	-1.465287	-2.626103
H	-4.129803	-0.532220	-2.450298
H	-5.518502	0.182141	-3.306655
H	-5.613826	4.883364	-0.836199
H	-6.094948	-3.078507	2.380531
H	3.517207	2.925676	-3.132057
H	3.942322	1.232675	-3.425233
H	2.302332	1.659721	-2.862197
H	3.256334	3.910465	-1.100789
H	1.975132	2.780257	-0.604636
H	3.349400	3.091335	0.463789
H	5.533767	3.089694	-1.750778
H	5.698361	2.237145	-0.210418
H	5.938696	1.362939	-1.738723
H	2.818631	-2.197473	-3.466958
H	1.667736	-1.316994	-2.445979
H	2.736960	-0.427844	-3.551298
H	3.687105	-3.403032	-1.754231
H	4.430359	-2.742713	-0.286738
H	2.659950	-2.687522	-0.509090
H	5.307018	-2.052719	-3.021265
H	5.256861	-0.284902	-3.040346
H	5.942903	-1.139443	-1.640996
H	3.809054	-1.232594	2.926350
H	2.339365	-0.946816	1.968058
H	3.501598	-2.192262	1.470465
H	6.073471	-0.581781	2.045515
H	5.808330	-1.474804	0.542437
H	6.261718	0.242137	0.486598
H	4.390484	0.953206	2.977948
H	4.673788	1.994082	1.574146
H	3.009725	1.514471	2.009799
H	-1.796012	-3.444711	2.113719
H	-0.129471	-4.557971	3.556581
H	1.432883	-3.210420	4.959100
H	1.288189	-0.718500	4.897732
H	-0.418634	0.394202	3.489773
H	-0.295758	2.485917	2.635221
H	1.543860	4.087819	3.048911
H	1.917563	6.000760	1.486151
H	0.458114	6.234444	-0.523415
H	-1.330768	4.597944	-0.971822
C	1.622143	-5.624361	-3.825398
C	-0.255972	-3.332519	-0.948595

H	1.177435	-5.395878	-4.807732
C	3.144387	-5.424870	-3.896819
H	1.403568	-6.688022	-3.636477
C	-1.709604	-3.020399	-1.331454
H	-0.230562	-3.854009	0.021852
H	0.280731	-2.378384	-0.781232
H	-2.197834	-2.449064	-0.533026
H	-1.748412	-2.427318	-2.254537
H	-2.281015	-3.943518	-1.493286
H	3.576586	-6.070980	-4.672183
H	3.391985	-4.383613	-4.140888
H	3.616573	-5.672159	-2.937188
C	0.434685	-4.134002	-1.949928
C	0.968226	-4.812294	-2.804017

110

RC_{anti}

C	-3.080164	2.091367	0.820296
C	-6.992262	-0.231348	-1.259755
C	-3.703241	1.081280	0.072263
C	-4.987199	1.086783	-0.438418
C	-5.716397	2.269415	-0.242517
C	-5.129821	3.335486	0.449341
C	-3.841447	3.265198	0.985280
C	-5.467084	-0.177215	-1.164214
C	-4.904847	-1.381195	-0.396468
C	-3.624551	-1.272297	0.111729
O	-2.912853	-0.072871	-0.059740
C	-5.556273	-2.600021	-0.150746
C	-4.901181	-3.597855	0.580857
C	-3.619215	-3.423623	1.109629
C	-2.937797	-2.208468	0.899180
N	-1.727958	-1.767781	1.415337
AL	-1.131525	0.006149	0.898390
C	-4.863080	-0.177774	-2.592459
N	-1.840453	1.755852	1.345099
C	-0.922406	2.727124	1.771769
C	-0.024851	2.393711	2.804713
C	0.998637	3.263154	3.176332
C	1.145567	4.496186	2.539097
C	0.256031	4.841509	1.517322
C	-0.762946	3.972851	1.133936
C	-0.748551	-2.660223	1.877888
C	-0.485842	-3.898772	1.261611
C	0.586223	-4.685134	1.676452
C	1.427165	-4.261033	2.710218
C	1.176656	-3.034452	3.327191
C	0.100889	-2.246468	2.922015
AU	1.229534	0.060070	0.402054
P	3.564948	0.109112	-0.172767

C	3.851255	-1.076269	-1.653536
C	3.037875	-2.360676	-1.389596
C	4.049908	1.897041	-0.672846
C	2.884136	2.482841	-1.495388
C	4.633009	-0.453370	1.317720
C	4.024669	0.174892	2.588591
C	4.128697	2.760981	0.597087
C	5.362153	2.027401	-1.455629
C	4.478636	-1.973890	1.490533
C	6.124499	-0.108264	1.224074
C	3.230418	-0.440422	-2.909033
C	5.314406	-1.436429	-1.937861
H	-3.439913	4.100045	1.556739
H	-6.734985	2.359248	-0.615191
H	-6.567603	-2.770216	-0.515060
H	-3.163302	-4.207363	1.712273
H	-7.309084	-1.134534	-1.796229
H	-7.370716	0.630498	-1.823536
H	-7.459123	-0.232380	-0.266286
H	-5.153165	-1.096874	-3.120012
H	-3.767519	-0.118493	-2.564702
H	-5.238770	0.687391	-3.155996
H	-5.706363	4.249866	0.596353
H	-5.417394	-4.540954	0.765984
H	3.053126	-2.971882	-2.305338
H	3.436476	-2.969691	-0.575066
H	1.992592	-2.119597	-1.150282
H	3.237178	-1.195900	-3.709816
H	2.186565	-0.143562	-2.736428
H	3.795232	0.424508	-3.272620
H	5.351863	-2.081508	-2.829985
H	5.931698	-0.553896	-2.140298
H	5.772614	-1.995254	-1.113947
H	3.095159	3.547472	-1.680948
H	2.742524	1.994464	-2.462380
H	1.941872	2.411584	-0.934749
H	4.231742	3.811114	0.283565
H	3.210903	2.685731	1.195201
H	4.991730	2.520579	1.226473
H	5.557668	3.095700	-1.639937
H	6.219507	1.623049	-0.905214
H	5.315589	1.532643	-2.432352
H	4.941977	-2.252374	2.449580
H	3.423572	-2.274261	1.532941
H	4.984818	-2.545049	0.705382
H	6.637373	-0.518075	2.108695
H	6.598222	-0.541956	0.335751
H	6.304053	0.972553	1.216135
H	4.544012	-0.247413	3.462839
H	4.129477	1.261888	2.628964

H	2.955634	-0.067891	2.668045
H	-0.145611	1.438731	3.318128
H	1.682631	2.973659	3.975463
H	1.943389	5.179077	2.831363
H	0.364931	5.796404	1.000587
H	-1.425123	4.243050	0.311874
H	-1.110377	-4.226307	0.430684
H	0.775911	-5.635892	1.175602
H	2.267038	-4.878995	3.028325
H	1.822209	-2.685551	4.134364
H	-0.100221	-1.295444	3.417457
C	-0.968690	-1.874304	-2.450749
C	-0.784596	2.244237	-2.483514
C	-0.325074	-2.597625	-3.642040
H	-0.469534	-2.179422	-1.513770
H	-2.020339	-2.189730	-2.347145
C	-2.120570	2.930992	-2.805286
H	-0.456549	2.524552	-1.467515
H	-0.001406	2.613760	-3.165151
H	-2.013144	4.021462	-2.731529
H	-2.452522	2.683403	-3.821833
H	-2.901639	2.613449	-2.103328
H	-0.388847	-3.685400	-3.506771
H	-0.831417	-2.334117	-4.579612
H	0.732797	-2.322226	-3.736652
C	-0.842132	0.790343	-2.550753
C	-0.910298	-0.421659	-2.539328

110

TSI_{syn}

C	0.011612	-3.154853	-1.569041
C	0.572814	-2.889108	-0.309832
C	0.133189	-3.644774	0.790631
C	-0.811779	-4.655647	0.628214
C	-1.353982	-4.922136	-0.632043
C	-0.944232	-4.159669	-1.726138
N	1.557994	-1.883526	-0.171838
C	2.789725	-2.265071	0.328002
C	3.734500	-1.232124	0.456449
C	5.007881	-1.322633	0.983654
C	5.413347	-2.607198	1.389298
C	4.541815	-3.687826	1.227402
C	3.252275	-3.543211	0.705059
C	5.883634	-0.063794	1.007935
C	4.982837	1.169241	1.150284
C	3.715556	1.127117	0.604623
O	3.226567	-0.023577	-0.000458
C	5.360799	2.396707	1.725975
C	4.471057	3.474391	1.697910
C	3.190973	3.380456	1.140945

C	2.758181	2.156368	0.590439
N	1.542864	1.824006	0.025685
AL	1.187859	-0.000919	-0.606930
C	0.478856	0.130978	-2.736158
C	1.734707	0.115642	-2.654041
C	6.923887	-0.128817	2.131129
C	6.613670	0.036186	-0.356416
C	0.579108	2.857513	-0.077361
C	0.009530	3.445958	1.062709
C	-0.900109	4.495549	0.941207
C	-1.268511	4.968936	-0.320507
C	-0.725645	4.374354	-1.460351
C	0.191734	3.329990	-1.340604
AU	-1.317544	-0.004186	-0.467655
P	-3.617172	0.000226	0.306738
C	-3.598813	0.361140	2.190599
C	-4.900116	0.034566	2.934738
C	-4.625532	1.359660	-0.599336
C	-5.951961	1.750805	0.065451
C	-4.436934	-1.703872	-0.008882
C	-5.965930	-1.733015	0.111429
C	-3.724199	2.599604	-0.730497
C	-4.903542	0.889713	-2.036948
C	-4.013285	-2.176229	-1.414130
C	-3.832157	-2.718443	0.973398
C	-2.428186	-0.430052	2.810054
C	-3.257502	1.845340	2.398861
C	-0.780541	0.193297	-3.509960
H	2.603166	-4.411199	0.606990
H	6.402533	-2.767573	1.813787
H	6.343258	2.517095	2.178210
H	2.526137	4.242281	1.148011
H	7.562018	0.763296	2.117277
H	7.581519	-0.996659	1.998082
H	6.443552	-0.201034	3.115319
H	7.232534	0.943543	-0.388163
H	5.891611	0.080393	-1.182414
H	7.257630	-0.841439	-0.506434
H	4.874975	-4.684338	1.521896
H	4.782352	4.427564	2.128475
H	-4.224312	3.325720	-1.389971
H	-3.524156	3.098011	0.219762
H	-2.753870	2.343064	-1.176063
H	-5.311514	1.743233	-2.599317
H	-3.981387	0.569151	-2.540194
H	-5.637819	0.079588	-2.090308
H	-6.453565	2.499328	-0.568353
H	-6.633925	0.900860	0.178737
H	-5.802905	2.207824	1.049983
H	-3.059152	2.000276	3.470455

H	-2.354901	2.134778	1.845537
H	-4.077253	2.514768	2.117807
H	-2.313741	-0.109649	3.857092
H	-2.583818	-1.511454	2.805224
H	-1.489732	-0.218478	2.277224
H	-4.783236	0.329998	3.989422
H	-5.761189	0.579625	2.531650
H	-5.132066	-1.036003	2.921554
H	-6.307598	-2.770443	-0.031716
H	-6.315642	-1.401969	1.095779
H	-6.458073	-1.121166	-0.652948
H	-4.352711	-3.215450	-1.543480
H	-4.445733	-1.580476	-2.221567
H	-2.919911	-2.166188	-1.514567
H	-4.150692	-3.724926	0.662667
H	-2.735827	-2.702062	0.945906
H	-4.171839	-2.568205	2.003427
H	0.545442	-3.428938	1.776653
H	-1.137474	-5.232162	1.495513
H	-2.096348	-5.711000	-0.756203
H	-1.365371	-4.352480	-2.714134
H	0.355049	-2.573257	-2.424017
H	0.635789	2.871841	-2.224124
H	-1.011597	4.731243	-2.451075
H	-1.980727	5.789257	-0.413435
H	-1.329882	4.943201	1.838775
H	0.295360	3.071379	2.046373
C	3.107135	0.146630	-3.201762
C	3.173958	0.276336	-4.728072
H	3.651201	0.978334	-2.725570
H	3.630922	-0.765717	-2.873470
C	-0.511148	0.245394	-5.020253
H	-1.402631	-0.679096	-3.257499
H	-1.353124	1.076941	-3.188165
H	4.220287	0.292284	-5.063865
H	2.667933	-0.566914	-5.215537
H	2.690498	1.203357	-5.062644
H	-1.463633	0.293057	-5.564997
H	0.083951	1.128302	-5.285687
H	0.034325	-0.646451	-5.354031

110

TSI$_{anti}$

C	-3.452035	-2.052105	-0.520731
C	-4.274758	-1.127947	0.139618
C	-5.650084	-1.163761	0.268614
C	-6.279533	-2.316861	-0.221801
C	-5.506125	-3.311908	-0.829557
C	-4.123528	-3.196413	-0.997976
C	-6.331345	0.074265	0.873212

C	-5.620262	1.294721	0.268468
C	-4.247747	1.220507	0.136695
O	-3.594012	0.039374	0.552761
C	-6.215425	2.467984	-0.217941
C	-5.412568	3.442569	-0.821771
C	-4.033781	3.286817	-0.993661
C	-3.398588	2.119732	-0.522084
N	-2.085406	1.688178	-0.704228
AL	-1.691021	0.000520	0.092264
C	-0.951941	-0.649586	2.149545
C	-0.258259	0.360595	1.521480
AU	1.506153	0.127589	0.374696
P	3.794068	-0.048877	-0.254843
C	4.741406	1.143375	0.915743
C	3.985183	2.487280	0.961627
C	-7.831092	0.094012	0.578264
C	-6.113532	0.070984	2.407960
N	-2.122012	-1.668802	-0.703401
C	-1.205964	-2.556660	-1.304234
C	-0.342846	-2.081197	-2.304199
C	0.587027	-2.925750	-2.909006
C	0.680829	-4.263347	-2.524537
C	-0.162217	-4.742552	-1.518256
C	-1.093198	-3.902902	-0.912901
C	-1.135975	2.545287	-1.296781
C	-0.955492	3.875998	-0.877347
C	0.017895	4.679484	-1.466354
C	0.835764	4.177377	-2.482567
C	0.667767	2.857103	-2.900940
C	-0.305053	2.050053	-2.313830
C	4.366890	-1.852877	0.068026
C	3.658508	-2.366963	1.336612
C	4.169365	0.423467	-2.075174
C	3.052272	-0.179070	-2.947516
C	3.848805	-2.736707	-1.078132
C	5.882462	-2.041266	0.214493
C	4.056837	1.949041	-2.223775
C	5.539234	-0.026203	-2.600145
C	4.677659	0.572391	2.343294
C	6.208086	1.395928	0.542291
H	-3.577121	-3.968915	-1.534460
H	-7.359533	-2.431522	-0.153296
H	-7.291322	2.615503	-0.147468
H	-3.463293	4.045245	-1.525721
H	-8.293939	0.983812	1.023231
H	-8.317981	-0.783790	1.021354
H	-8.031492	0.097848	-0.500937
H	-6.559716	0.972527	2.848877
H	-5.046908	0.049497	2.662913
H	-6.593528	-0.812853	2.849305

H	-6.002043	-4.203823	-1.214757
H	-5.882280	4.351359	-1.200390
H	4.421951	3.097273	1.766851
H	4.058645	3.058391	0.033460
H	2.920030	2.336215	1.186190
H	5.060011	1.339600	3.033087
H	3.645863	0.339497	2.639664
H	5.295835	-0.321987	2.471537
H	6.655848	2.045320	1.310827
H	6.798006	0.473823	0.503476
H	6.311244	1.912486	-0.418344
H	3.909431	-3.432058	1.457368
H	3.960225	-1.848422	2.249496
H	2.567910	-2.283764	1.239467
H	4.015469	-3.787531	-0.796873
H	2.770602	-2.606553	-1.235475
H	4.372236	-2.564672	-2.024238
H	6.089349	-3.117511	0.323275
H	6.440533	-1.682748	-0.657486
H	6.278103	-1.543874	1.107249
H	4.077255	2.185615	-3.298475
H	3.111076	2.329108	-1.819282
H	4.890395	2.482956	-1.756412
H	5.653075	0.341039	-3.632292
H	6.367518	0.382429	-2.010352
H	5.641682	-1.116322	-2.630284
H	3.154957	0.219537	-3.968506
H	3.098184	-1.268598	-3.006747
H	2.062643	0.100723	-2.563212
H	-0.432199	-1.044298	-2.628157
H	1.232115	-2.535355	-3.696224
H	1.404279	-4.925556	-3.000236
H	-0.089600	-5.781606	-1.194125
H	-1.728059	-4.281611	-0.113548
H	-1.572513	4.268309	-0.069859
H	0.144490	5.706486	-1.120887
H	1.595029	4.809935	-2.942855
H	1.291668	2.451232	-3.697247
H	-0.456554	1.029380	-2.665171
C	-0.471585	1.804074	1.999773
C	-0.492737	-2.065876	1.942935
C	0.320682	-2.469082	3.185702
H	-1.343964	-2.756579	1.843619
H	0.159284	-2.199155	1.049610
C	0.402749	2.062651	3.231425
H	-0.219624	2.527791	1.213720
H	-1.528397	1.953115	2.279236
H	0.668020	-3.508449	3.098457
H	1.198015	-1.816476	3.294908
H	-0.285086	-2.371873	4.096669

H	0.282244	3.093443	3.594251
H	0.129813	1.365693	4.035431
H	1.465191	1.901961	2.995762

110

TSI_{syn}^{Au}

C	0.424774	-2.318114	3.231816
C	0.585806	-2.301522	1.840341
C	-0.140878	-3.208342	1.056766
C	-0.988544	-4.138199	1.659777
C	-1.124628	-4.169089	3.049270
C	-0.424518	-3.248796	3.832325
N	1.524589	-1.425999	1.219855
AL	1.109140	0.135950	0.183987
AU	-1.203033	-0.214431	-0.716491
P	-3.290783	0.029996	0.202989
C	-3.890729	1.737230	-0.455636
C	-3.396819	1.914290	-1.905641
N	1.731653	1.945509	0.405360
C	3.062430	2.207875	0.112417
C	3.829634	1.093480	-0.252207
C	5.199423	1.034893	-0.421556
C	5.879047	2.258662	-0.314711
C	5.157457	3.422959	-0.026464
C	3.777298	3.419819	0.196473
C	5.819498	-0.345698	-0.680437
C	5.066945	-1.349184	0.205853
C	3.706103	-1.170684	0.349957
O	3.074279	-0.091329	-0.290437
C	5.611716	-2.447645	0.891844
C	4.770170	-3.267991	1.651966
C	3.401258	-3.017729	1.798557
C	2.827467	-1.901241	1.157898
C	7.321847	-0.354081	-0.396332
C	5.579406	-0.725805	-2.164486
C	0.899720	3.039836	0.747709
C	0.301437	3.092892	2.013388
C	-0.522889	4.162147	2.367170
C	-0.757702	5.199109	1.464118
C	-0.167249	5.152546	0.198173
C	0.650899	4.083157	-0.159105
C	-4.450522	-1.355718	-0.486678
C	-3.721961	-2.705143	-0.345986
C	-3.410555	0.015752	2.121035
C	-2.193756	0.754960	2.697183
C	-5.834582	-1.445578	0.169671
C	-4.626087	-1.132671	-1.998372
C	-4.698075	0.637044	2.681872
C	-3.294817	-1.432539	2.614228
C	-5.410994	1.943459	-0.414092

C	-3.205353	2.847334	0.356150
H	2.786904	-3.662705	2.425106
H	6.677104	-2.662863	0.836924
H	6.959128	2.305439	-0.440016
H	3.263068	4.339837	0.469185
H	7.838650	0.357549	-1.052542
H	7.742549	-1.347333	-0.597110
H	7.536514	-0.088050	0.646680
H	6.078611	-0.001706	-2.822765
H	4.508992	-0.732772	-2.404816
H	5.985657	-1.726562	-2.364494
H	5.199124	-4.128735	2.167328
H	5.694554	4.369216	0.053549
H	-5.123944	-2.020773	-2.416190
H	-5.249902	-0.264514	-2.234998
H	-3.652316	-1.028656	-2.496586
H	-4.312978	-3.468292	-0.875619
H	-2.726833	-2.657544	-0.809969
H	-3.604763	-3.030692	0.690069
H	-6.415028	-2.223518	-0.350618
H	-5.780206	-1.736247	1.224331
H	-6.399630	-0.509722	0.097215
H	-3.677751	2.925861	-2.237790
H	-2.301742	1.834053	-1.957991
H	-3.829822	1.196819	-2.606525
H	-3.414809	3.808602	-0.136521
H	-3.576119	2.920204	1.383658
H	-2.117440	2.712782	0.378753
H	-5.626250	2.970850	-0.746887
H	-5.944484	1.265451	-1.089345
H	-5.827688	1.828904	0.591950
H	-2.244380	0.688567	3.795090
H	-1.261963	0.282475	2.362442
H	-2.150991	1.809501	2.419198
H	-4.693221	0.502758	3.774699
H	-4.764418	1.713249	2.488711
H	-5.603737	0.157948	2.295118
H	-3.161233	-1.410172	3.705966
H	-4.186800	-2.031318	2.404563
H	-2.418453	-1.933108	2.189249
H	1.108964	4.044821	-1.147605
H	-0.350883	5.952880	-0.520003
H	-1.398287	6.036366	1.741717
H	-0.977648	4.186122	3.358411
H	0.502135	2.289025	2.722271
H	0.988807	-1.606347	3.835967
H	-0.537361	-3.257119	4.917195
H	-1.783775	-4.899242	3.519808
H	-1.544908	-4.842084	1.039513
H	-0.025815	-3.172242	-0.027996

C	-0.604118	-1.897181	-3.831527
C	1.380369	0.552126	-2.821768
H	0.330156	-1.922588	-4.426400
C	-1.237265	-3.291730	-3.798398
H	-1.286093	-1.210395	-4.366713
C	0.587966	1.764109	-3.320931
H	2.246214	0.909269	-2.249026
H	1.791364	-0.013165	-3.675480
H	-1.430420	-3.669290	-4.812830
H	-0.575958	-4.004615	-3.286574
H	-2.186432	-3.265123	-3.245633
H	1.237743	2.464457	-3.864749
H	-0.227483	1.450964	-3.986455
H	0.141611	2.297725	-2.469654
C	0.488578	-0.403336	-2.053015
C	-0.364764	-1.311013	-2.485941

110

PC$_{syn}$

C	-5.718932	-1.685257	-1.103829
C	-4.857635	-1.301208	-0.060912
C	-4.911184	-2.016249	1.148630
C	-5.771679	-3.102172	1.300893
C	-6.616306	-3.481885	0.256381
C	-6.587523	-2.761742	-0.941404
N	-3.893473	-0.284015	-0.197437
AL	-2.030051	-0.538751	0.165945
C	-4.222802	0.957733	-0.717389
C	-3.176748	1.893564	-0.762464
C	-3.290324	3.256140	-0.963592
C	-4.559253	3.722682	-1.335754
C	-5.622123	2.816493	-1.419234
C	-5.483382	1.464012	-1.101083
O	-1.928256	1.383122	-0.301558
C	-1.442894	2.161297	0.786221
C	-1.498069	3.537837	0.672275
C	-2.059313	4.112915	-0.638568
C	-1.096807	4.262599	1.803540
C	-0.716732	3.572703	2.960602
C	-0.719158	2.178869	3.040841
C	-1.092107	1.410120	1.919206
N	-1.234007	0.033094	1.793427
C	-0.637390	-0.884858	2.670809
C	0.570978	-0.641880	3.349225
C	1.152530	-1.627642	4.142883
C	0.556456	-2.883219	4.278925
C	-0.636184	-3.140462	3.599368
C	-1.227345	-2.154743	2.814922
C	-2.421321	5.590896	-0.498143
C	-1.008942	3.957482	-1.765707

AU	1.560911	-0.845575	-0.603637
P	3.544241	0.428761	-0.407314
C	3.249409	2.255697	0.085601
C	2.194455	2.260956	1.203674
C	4.698769	-0.395946	0.887812
C	4.558383	-1.925683	0.749647
C	4.413266	0.383619	-2.125360
C	3.339739	0.590630	-3.213107
C	6.177919	0.000622	0.796079
C	4.179426	-0.058475	2.294365
C	5.535774	1.412759	-2.313492
C	4.988734	-1.022351	-2.365726
C	4.486074	3.041048	0.540138
C	2.600348	2.976009	-1.107443
H	-0.463708	1.688567	3.977438
H	-1.109034	5.350780	1.796484
H	-4.727143	4.780741	-1.526338
H	-6.356938	0.815832	-1.093935
H	-2.818937	5.975784	-1.445552
H	-1.527951	6.179145	-0.252862
H	-3.171683	5.752060	0.286478
H	-1.424818	4.325868	-2.712911
H	-0.714761	2.910229	-1.901505
H	-0.111169	4.541056	-1.523466
H	-0.427889	4.143108	3.844421
H	-6.609321	3.184700	-1.702096
H	5.140582	-2.396267	1.556881
H	3.508588	-2.231042	0.858585
H	4.931193	-2.314128	-0.201141
H	4.721185	-0.688817	3.015861
H	4.353392	0.985445	2.574936
H	3.111392	-0.288047	2.389496
H	6.719361	-0.483944	1.623725
H	6.646635	-0.332906	-0.136060
H	6.329291	1.081686	0.890246
H	2.247792	3.958179	-0.758909
H	3.295879	3.149758	-1.934552
H	1.728902	2.423615	-1.482413
H	1.830846	3.288849	1.347368
H	1.335820	1.637009	0.929839
H	2.581207	1.908986	2.162910
H	4.180145	4.078498	0.747673
H	4.915356	2.639613	1.464924
H	5.272938	3.073532	-0.221012
H	5.992138	1.249797	-3.302429
H	5.166493	2.443979	-2.293573
H	6.328256	1.314726	-1.563492
H	5.326449	-1.074296	-3.411968
H	5.852817	-1.245743	-1.732224
H	4.229275	-1.799965	-2.222910

H	3.809398	0.424096	-4.194707
H	2.518296	-0.129502	-3.094457
H	2.911808	1.596211	-3.214532
H	-2.162113	-2.373279	2.294257
H	-1.116295	-4.116139	3.684240
H	1.016154	-3.650788	4.901462
H	2.095503	-1.414427	4.648967
H	1.071235	0.315618	3.224127
H	-5.674406	-1.160972	-2.057332
H	-7.237801	-3.052769	-1.767562
H	-7.293252	-4.328036	0.373945
H	-5.789834	-3.645424	2.246531
H	-4.277645	-1.695265	1.977695
H	0.384047	-4.312825	-1.054143
H	-0.125581	-3.677810	-2.607390
H	2.203425	-4.454566	-2.734661
H	2.167077	-2.692197	-2.966526
H	2.686275	-3.368596	-1.410223
H	-1.840602	-4.101612	-1.276653
H	-3.142755	-3.152196	-0.587331
C	-2.810307	-2.762209	-2.676140
H	-3.570113	-3.485680	-3.005681
H	-3.267034	-1.762715	-2.686774
H	-1.991814	-2.756927	-3.409609
C	0.533225	-3.467029	-1.749734
C	1.978482	-3.497090	-2.241617
C	-1.275376	-2.054278	-0.783684
C	-2.284487	-3.094266	-1.272704
C	0.057014	-2.197274	-1.036717

110

PC$_{syn}$'

C	1.622822	1.668420	-2.929035
C	0.395783	2.265454	-2.616613
C	0.127487	3.553127	-3.101637
C	1.069280	4.230953	-3.875635
C	2.295874	3.632646	-4.174200
C	2.568093	2.348934	-3.695921
N	-0.563443	1.601076	-1.799028
C	-1.681515	1.093679	-2.428096
C	-2.679835	0.626415	-1.554817
C	-3.861888	0.001732	-1.891195
C	-4.084391	-0.146167	-3.274495
C	-3.143103	0.324925	-4.194236
C	-1.950102	0.940285	-3.799431
C	-4.860584	-0.438364	-0.814112
C	-4.222390	-0.371882	0.578627
C	-3.021903	0.276202	0.786539
O	-2.292845	0.848966	-0.242682
C	-4.821241	-0.901375	1.737561

C	-4.201857	-0.753619	2.981719
C	-2.978425	-0.094398	3.136225
C	-2.340747	0.444777	2.005134
N	-1.114217	1.076629	1.898245
AL	-0.386520	1.523591	0.141755
AU	1.656092	-0.298632	0.138463
P	1.189390	-2.628720	-0.101624
C	2.852055	-3.459604	-0.583643
C	2.890059	-4.984429	-0.419318
C	-6.070648	0.525689	-0.845852
C	-5.346712	-1.872357	-1.099272
C	-0.491133	1.511155	3.098543
C	-1.149670	2.407501	3.956965
C	-0.530431	2.866559	5.117957
C	0.763896	2.448680	5.440698
C	1.425962	1.561686	4.590192
C	0.803240	1.092757	3.434012
C	1.182975	2.617425	0.475129
C	2.182471	1.705574	0.377515
C	0.563435	-3.369617	1.552973
C	-0.010008	-4.789392	1.451608
C	-0.099737	-2.955609	-1.481745
C	-0.125060	-4.390260	-2.025928
C	1.727497	-3.365159	2.558495
C	-0.504263	-2.427438	2.137656
C	-1.491980	-2.608230	-0.934745
C	0.163632	-1.966489	-2.632309
C	3.171876	-3.099181	-2.044291
C	3.977280	-2.827932	0.258545
H	-1.228321	1.286350	-4.537930
H	-4.996974	-0.620470	-3.632823
H	-5.775899	-1.421222	1.670049
H	-2.516205	-0.005055	4.117721
H	-6.806873	0.238524	-0.082885
H	-6.554599	0.496595	-1.831441
H	-5.749418	1.556374	-0.647410
H	-6.092737	-2.182325	-0.357140
H	-4.511089	-2.582463	-1.068224
H	-5.818321	-1.932046	-2.087692
H	-3.342260	0.205341	-5.260314
H	-4.687056	-1.170350	3.865557
H	4.937695	-3.234108	-0.094439
H	4.005546	-1.738550	0.126307
H	3.897916	-3.040250	1.326886
H	4.202863	-3.420940	-2.254686
H	2.517403	-3.600941	-2.763584
H	3.119822	-2.014922	-2.212273
H	3.858390	-5.349176	-0.795883
H	2.814467	-5.289914	0.630233
H	2.101601	-5.491544	-0.985536

H	-2.189910	-2.562219	-1.782706
H	-1.871354	-3.351881	-0.226496
H	-1.504268	-1.625979	-0.449554
H	-0.655750	-2.059810	-3.360495
H	0.165664	-0.931699	-2.268939
H	1.103015	-2.151231	-3.159005
H	-0.947544	-4.466622	-2.753888
H	0.798272	-4.656192	-2.551953
H	-0.305047	-5.135369	-1.243065
H	-0.280101	-5.125513	2.464708
H	-0.922085	-4.825678	0.845494
H	0.705784	-5.510307	1.042621
H	1.318038	-3.611133	3.549444
H	2.496604	-4.109266	2.326883
H	2.196143	-2.374232	2.629628
H	-0.729240	-2.759636	3.162059
H	-0.145043	-1.392806	2.189938
H	-1.444269	-2.429488	1.582142
H	-0.826543	4.020468	-2.853876
H	0.847956	5.235802	-4.238162
H	3.036206	4.165404	-4.772011
H	3.524476	1.873677	-3.919649
H	1.836632	0.675076	-2.535891
H	1.325174	0.413751	2.760511
H	2.437807	1.228913	4.827348
H	1.251768	2.814121	6.344875
H	-1.057532	3.566001	5.768557
H	-2.153612	2.746556	3.698147
C	1.406242	4.092250	0.677880
C	3.644290	2.108279	0.450192
C	4.667979	0.993880	0.259193
H	3.815889	2.893637	-0.308377
H	3.820021	2.601262	1.424181
H	1.997667	4.495038	-0.166219
C	0.102869	4.879355	0.812088
H	2.030106	4.254601	1.576581
H	5.695411	1.384416	0.281195
H	4.579962	0.236605	1.051911
H	4.520254	0.486639	-0.706184
H	0.279447	5.954513	0.959846
H	-0.512155	4.759802	-0.093178
H	-0.481780	4.508547	1.667204

110

PC$_{trans}$

C	4.732160	-1.787590	-0.331141
C	4.926802	-0.493426	-0.836417
C	6.088828	0.033431	-1.365539
C	7.148747	-0.870642	-1.533278
C	6.985776	-2.201383	-1.130735

C	5.815771	-2.667904	-0.526337
C	6.113283	1.543716	-1.642070
C	5.398501	2.208421	-0.455664
C	4.261387	1.589333	0.029120
O	3.838187	0.380398	-0.582721
C	5.797528	3.368890	0.222960
C	5.062638	3.805025	1.332432
C	3.954066	3.109456	1.820119
C	3.526201	1.931990	1.174012
N	2.541472	1.027061	1.552358
AL	2.365284	-0.495210	0.413175
AU	-2.276515	0.146591	-0.704966
P	-4.607754	0.136029	-0.459890
C	-5.454235	1.167793	-1.838461
C	-4.601629	2.430547	-2.077292
C	7.541495	2.064589	-1.804479
C	5.316080	1.838496	-2.938061
N	3.548441	-1.986612	0.366114
C	3.193492	-3.259994	0.853795
C	2.726551	-3.374312	2.173030
C	2.275215	-4.597099	2.668667
C	2.302900	-5.735250	1.862405
C	2.782277	-5.634415	0.552297
C	3.219863	-4.412679	0.047952
C	1.478868	1.388342	2.398532
C	0.895663	2.669466	2.401021
C	-0.233987	2.931168	3.172056
C	-0.816916	1.929478	3.953023
C	-0.243398	0.657249	3.960497
C	0.895290	0.394741	3.203797
C	-5.251411	-1.670853	-0.523494
C	-4.513733	-2.397312	-1.666426
C	-5.022442	0.900477	1.252705
C	-3.997284	0.378597	2.279927
C	-4.824666	-2.389964	0.767723
C	-6.767287	-1.822777	-0.705262
C	-4.795039	2.420157	1.180042
C	-6.444602	0.627749	1.758976
C	-5.395912	0.370262	-3.152346
C	-6.908490	1.569707	-1.560973
H	5.757160	-3.693660	-0.167099
H	8.097313	-0.540769	-1.952264
H	6.680460	3.919735	-0.095437
H	3.446259	3.450565	2.720252
H	7.532568	3.143509	-2.003449
H	8.028566	1.578604	-2.659277
H	8.144579	1.878900	-0.906487
H	5.297725	2.920393	-3.126006
H	4.281291	1.482586	-2.864154
H	5.793984	1.338937	-3.791545

H	7.815192	-2.897432	-1.262605
H	5.382607	4.707001	1.855757
H	-5.000602	2.952019	-2.960897
H	-4.618734	3.131942	-1.239853
H	-3.555020	2.161836	-2.276890
H	-5.710638	1.037946	-3.968535
H	-4.374681	0.031392	-3.371816
H	-6.068240	-0.493823	-3.157853
H	-7.298288	2.103160	-2.442096
H	-7.557000	0.704340	-1.383780
H	-6.997475	2.248585	-0.705618
H	-4.772329	-3.466139	-1.615940
H	-4.785518	-2.035034	-2.660740
H	-3.424988	-2.300310	-1.551617
H	-5.032876	-3.463196	0.641086
H	-3.748426	-2.278413	0.953698
H	-5.377971	-2.050804	1.649432
H	-7.017975	-2.894636	-0.673531
H	-7.337339	-1.325901	0.087613
H	-7.112864	-1.437699	-1.671218
H	-4.855005	2.819724	2.203696
H	-3.797233	2.657096	0.787154
H	-5.550336	2.938581	0.580506
H	-6.584868	1.162518	2.711472
H	-7.215243	0.980268	1.064561
H	-6.618630	-0.435985	1.956470
H	-4.172688	0.904369	3.231286
H	-4.078944	-0.693623	2.472769
H	-2.968896	0.593314	1.959534
H	2.739898	-2.490516	2.814120
H	1.910865	-4.660265	3.694795
H	1.956495	-6.693702	2.249260
H	2.800308	-6.515579	-0.090555
H	3.557591	-4.334455	-0.985434
H	1.310081	3.449988	1.764764
H	-0.675573	3.928646	3.146719
H	-1.707935	2.138473	4.545201
H	-0.682367	-0.139437	4.562327
H	1.347061	-0.598526	3.226177
C	0.378060	1.579277	-0.941781
C	-0.105134	-2.190542	0.016159
C	-0.688519	-2.400345	1.417720
H	-0.916187	-2.310288	-0.723104
H	0.627970	-2.987505	-0.186706
H	1.465746	1.609834	-0.742685
C	0.150963	2.010683	-2.395458
H	-0.074463	2.330513	-0.272961
H	0.569408	3.009777	-2.583897
H	0.622739	1.300616	-3.089683
H	-0.924426	2.032466	-2.625601

H	-1.177528	-3.382086	1.502047
H	0.092401	-2.351375	2.188393
H	-1.424657	-1.614323	1.639042
C	0.530278	-0.821633	-0.184361
C	-0.210430	0.222326	-0.634923

110

TS$_{syn/anti}$'

C	-4.473783	2.609774	-1.420572
C	-3.691197	2.140609	-0.346402
C	-3.230194	3.079661	0.598374
C	-3.526519	4.433211	0.468614
C	-4.303617	4.888441	-0.599994
C	-4.774886	3.965505	-1.537700
N	-3.256032	0.816981	-0.222510
C	-4.025423	-0.273178	-0.591626
C	-3.350776	-1.507097	-0.627087
C	-3.900558	-2.769621	-0.742630
C	-5.284603	-2.815715	-0.969299
C	-6.006323	-1.616460	-1.026031
C	-5.413829	-0.364873	-0.833893
C	-2.946330	-3.964881	-0.557842
C	-2.020312	-3.608040	0.617361
C	-1.593465	-2.296074	0.687521
O	-1.976035	-1.412443	-0.331625
C	-1.608954	-4.440892	1.668159
C	-0.832372	-3.901299	2.703102
C	-0.502084	-2.544881	2.769956
C	-0.929990	-1.668877	1.750115
N	-0.785015	-0.288071	1.649457
AL	-1.361922	0.484861	-0.026439
AU	1.452158	0.766370	-0.488862
P	3.374030	-0.495332	-0.363716
C	4.710408	0.401696	-1.408606
C	6.143575	-0.099849	-1.185111
C	-3.710566	-5.262589	-0.295908
C	-2.089754	-4.127639	-1.839905
C	-0.632550	0.487265	2.821754
C	-1.305758	0.168297	4.017827
C	-1.189363	0.983992	5.140603
C	-0.412458	2.145951	5.103821
C	0.245604	2.478046	3.918841
C	0.145060	1.658470	2.796461
C	3.985512	-0.695664	1.439903
C	5.045605	-1.787702	1.638901
C	3.004511	-2.225321	-1.117776
C	4.255808	-3.015216	-1.525542
C	4.547835	0.652901	1.918181
C	2.767279	-0.985230	2.334701
C	2.194903	-3.048822	-0.102571

C	2.083038	-2.046233	-2.340718
C	4.351406	0.260734	-2.897483
C	4.639087	1.910633	-1.099884
H	-6.031907	0.530833	-0.818550
H	-5.799936	-3.767805	-1.082207
H	-1.898081	-5.490046	1.694316
H	0.049966	-2.157904	3.624701
H	-3.010539	-6.098800	-0.172528
H	-4.360699	-5.501469	-1.147074
H	-4.330577	-5.190827	0.607088
H	-1.377069	-4.953677	-1.709818
H	-1.525699	-3.213703	-2.062472
H	-2.738951	-4.352616	-2.696921
H	-7.083022	-1.658598	-1.197439
H	-0.498814	-4.557320	3.508557
H	5.326756	2.430963	-1.783948
H	3.625118	2.298920	-1.271222
H	4.931273	2.161044	-0.077549
H	5.021311	0.919613	-3.469880
H	4.488093	-0.756755	-3.277295
H	3.319287	0.580309	-3.093839
H	6.810451	0.435594	-1.878332
H	6.501859	0.102269	-0.169668
H	6.251209	-1.171817	-1.384189
H	1.834699	-3.953528	-0.614825
H	2.790189	-3.374630	0.756304
H	1.313381	-2.505176	0.260132
H	1.816524	-3.048174	-2.710610
H	1.156242	-1.524647	-2.066046
H	2.549389	-1.500158	-3.164464
H	3.937885	-4.009773	-1.874388
H	4.799863	-2.541233	-2.350095
H	4.951353	-3.163855	-0.692367
H	5.367664	-1.767909	2.691378
H	4.650521	-2.790185	1.440412
H	5.934790	-1.635970	1.017468
H	4.724506	0.578471	3.001455
H	5.500921	0.912669	1.446097
H	3.829714	1.467448	1.753311
H	3.105497	-0.970662	3.381981
H	1.996143	-0.214558	2.218046
H	2.305522	-1.956567	2.147374
H	-2.628719	2.727277	1.439393
H	-3.150277	5.137942	1.211648
H	-4.539907	5.947814	-0.700771
H	-5.372625	4.307059	-2.384508
H	-4.814677	1.910260	-2.183107
H	0.657338	1.921254	1.872631
H	0.853969	3.382644	3.864827
H	-0.328672	2.784147	5.983742

H	-1.726101	0.715497	6.052009
H	-1.940041	-0.716797	4.055594
C	-0.069496	3.543623	-1.046468
C	-1.018163	1.368780	-3.172863
C	-1.313526	0.093104	-3.962635
H	-1.910900	2.023568	-3.170081
H	-0.227087	1.961764	-3.667327
H	-1.053179	3.971160	-1.307070
C	0.515730	4.297385	0.146030
H	0.571407	3.692526	-1.935835
H	-1.602549	0.312093	-5.000063
H	-0.431043	-0.562276	-3.983546
H	-2.129130	-0.469668	-3.486691
H	0.652797	5.365075	-0.074875
H	-0.143570	4.206634	1.020279
H	1.494744	3.877999	0.424629
C	-0.258228	2.065187	-0.799389
C	-0.666239	1.119954	-1.739913

110

TS_{side}

C	5.260701	1.551046	-2.492196
C	4.938009	1.246399	-1.160502
C	5.620890	1.911558	-0.132893
C	6.575857	2.885916	-0.426626
C	6.883800	3.189763	-1.753105
C	6.227855	2.510604	-2.783850
N	3.924340	0.306028	-0.842241
AL	2.300407	0.695132	0.088438
C	4.142536	-1.017652	-1.206465
C	3.162168	-1.933474	-0.799091
C	3.235837	-3.312049	-0.846980
C	4.369585	-3.844437	-1.481707
C	5.345477	-2.977125	-1.983641
C	5.260637	-1.588230	-1.846950
O	2.085626	-1.319537	-0.138488
C	1.726558	-1.971587	1.055728
C	1.700864	-3.352720	1.091374
C	2.097829	-4.105298	-0.188773
C	1.324577	-3.925627	2.316946
C	1.026316	-3.092634	3.402479
C	1.139732	-1.701696	3.336986
C	1.548828	-1.090267	2.133742
N	1.871059	0.237837	1.905965
C	1.364690	1.219168	2.789831
C	-0.015229	1.321093	3.039197
C	-0.518288	2.341239	3.840644
C	0.344799	3.283408	4.410303
C	1.716522	3.187474	4.170519
C	2.224300	2.160417	3.373095

C	2.517223	-5.544584	0.115494
C	0.890670	-4.124476	-1.157015
AU	-1.903159	0.782786	-0.624397
P	-3.747422	-0.589330	-0.307208
C	-3.208463	-2.308798	0.344056
C	-2.035493	-2.107188	1.323044
C	-4.840690	0.281490	1.009880
C	-4.876746	1.792819	0.706777
C	-4.730095	-0.784412	-1.940118
C	-3.719400	-1.027171	-3.078969
C	-6.275276	-0.254848	1.106728
C	-4.154703	0.151228	2.380253
C	-5.785442	-1.898258	-1.932282
C	-5.412769	0.555457	-2.263157
C	-4.320705	-3.121188	1.019702
C	-2.642193	-3.120982	-0.830945
H	0.952126	-1.089807	4.218530
H	1.274345	-5.006726	2.432566
H	4.501583	-4.920930	-1.571595
H	6.072363	-0.953341	-2.197300
H	2.786910	-6.068010	-0.810440
H	1.685442	-6.097002	0.570451
H	3.374732	-5.577903	0.799552
H	1.171220	-4.617743	-2.097521
H	0.551472	-3.107054	-1.386206
H	0.056084	-4.675232	-0.703666
H	0.724661	-3.547212	4.347427
H	6.223149	-3.398301	-2.476161
H	-5.409191	2.291282	1.531078
H	-3.859526	2.205652	0.654224
H	-5.393866	2.041013	-0.222780
H	-4.693821	0.795231	3.090951
H	-4.176266	-0.867861	2.778336
H	-3.113104	0.497248	2.339707
H	-6.785353	0.263516	1.933226
H	-6.852941	-0.059838	0.196260
H	-6.312011	-1.329210	1.316282
H	-2.184012	-4.031374	-0.418958
H	-3.409719	-3.434895	-1.545380
H	-1.858482	-2.567891	-1.363902
H	-1.627624	-3.093273	1.590026
H	-1.226265	-1.528637	0.858870
H	-2.321690	-1.606945	2.251124
H	-3.912477	-4.106437	1.292791
H	-4.679642	-2.652569	1.942421
H	-5.179257	-3.289671	0.359752
H	-6.317554	-1.881397	-2.895999
H	-5.342335	-2.894286	-1.823886
H	-6.530961	-1.764538	-1.140639
H	-5.816394	0.492033	-3.284762

H	-6.248996	0.780635	-1.593576
H	-4.697236	1.388142	-2.237654
H	-4.267360	-0.998270	-4.033006
H	-2.954253	-0.238739	-3.097457
H	-3.214355	-1.993639	-3.014457
H	3.296439	2.078977	3.189108
H	2.400067	3.914188	4.611865
H	-0.050268	4.086055	5.033555
H	-1.594049	2.413663	4.010810
H	-0.685527	0.604735	2.563717
H	4.729451	1.039283	-3.295532
H	6.460534	2.741351	-3.824239
H	7.632318	3.948225	-1.983985
H	7.088727	3.400668	0.386951
H	5.399374	1.648678	0.902772
H	-1.331951	3.659689	0.616727
H	0.105190	4.261056	-0.207213
H	-1.964305	5.239169	-1.213460
H	-1.142754	4.206563	-2.409438
H	-2.581813	3.602260	-1.566495
H	1.738950	3.401049	0.807518
H	3.304334	2.647595	0.631819
C	2.666124	3.659618	-1.176889
H	3.412862	4.451492	-1.002787
H	3.059196	3.020868	-1.978258
H	1.757469	4.134754	-1.568329
C	-0.380118	2.201855	-0.586187
C	-0.783437	3.627979	-0.339525
C	-1.670900	4.206245	-1.446041
C	0.752032	1.554538	-0.562340
C	2.360920	2.867491	0.084230

110

PC$_{side}$

C	-2.417630	-1.565424	3.908401
C	-1.377647	-0.893218	3.252080
C	-0.066124	-1.067955	3.722675
C	0.200457	-1.901933	4.805960
C	-0.841978	-2.582472	5.442411
C	-2.150703	-2.408771	4.987562
N	-1.641118	-0.087896	2.116072
C	-1.408136	1.268833	2.236615
C	-1.599184	2.030885	1.071049
C	-1.573007	3.408136	0.961521
C	-1.234012	4.115843	2.128177
C	-0.982262	3.407868	3.308827
C	-1.073459	2.014281	3.386289
C	-1.923461	4.005039	-0.410697
C	-3.046267	3.146170	-1.012605
C	-2.982576	1.783345	-0.794031

O	-1.956516	1.251729	-0.021237
C	-4.136769	3.603158	-1.772689
C	-5.079980	2.679678	-2.236760
C	-5.004824	1.313836	-1.943396
C	-3.936616	0.820025	-1.164643
N	-3.752865	-0.446445	-0.641981
AL	-2.176861	-0.894776	0.418111
C	-2.332760	5.474377	-0.298976
C	-0.682581	3.893075	-1.332267
C	-4.640062	-1.469422	-1.047569
C	-4.737300	-1.863728	-2.393409
C	-5.559106	-2.925198	-2.765599
C	-6.299127	-3.617981	-1.802252
C	-6.216554	-3.226491	-0.464545
C	-5.404435	-2.155207	-0.092133
AU	1.335970	-0.247738	-0.578980
P	3.413793	0.576226	-0.023208
C	4.298305	1.082162	-1.647346
C	4.663636	-0.190089	-2.430289
C	3.269559	2.084491	1.141613
C	2.796701	3.299409	0.327359
C	4.373406	-0.842334	0.839406
C	3.824313	-1.012823	2.265891
C	2.151961	1.798836	2.157858
C	4.568589	2.443220	1.876039
C	4.070477	-2.166978	0.110837
C	5.891595	-0.627484	0.901791
C	3.304778	1.862033	-2.531146
C	5.564156	1.923602	-1.434337
H	-0.909176	1.505524	4.335133
H	-1.183014	5.203191	2.124693
H	-4.257075	4.661861	-1.995242
H	-5.790515	0.638535	-2.279517
H	-2.563631	5.884838	-1.290175
H	-1.511016	6.070988	0.117418
H	-3.214016	5.597624	0.343630
H	-0.925358	4.265151	-2.337251
H	-0.352846	2.849185	-1.419443
H	0.143894	4.491178	-0.924084
H	-0.724583	3.963453	4.212272
H	-5.922580	3.038852	-2.830142
H	4.548573	-2.979684	0.677994
H	2.990277	-2.363981	0.080920
H	4.455591	-2.202533	-0.910715
H	4.268830	-1.925333	2.689471
H	4.083310	-0.182173	2.929330
H	2.734510	-1.147003	2.264287
H	6.333645	-1.454590	1.477561
H	6.354675	-0.640049	-0.091090
H	6.166178	0.307455	1.402002

H	2.533459	4.095348	1.039071
H	3.568910	3.695122	-0.339882
H	1.895991	3.072313	-0.255214
H	1.965004	2.715621	2.734520
H	1.210891	1.539517	1.656179
H	2.399153	1.005821	2.867165
H	4.385958	3.354105	2.465704
H	4.883731	1.661944	2.576105
H	5.398523	2.652457	1.192085
H	6.022964	2.111155	-2.416922
H	5.345456	2.898985	-0.986565
H	6.309550	1.416077	-0.812826
H	5.011216	0.115896	-3.427851
H	5.471298	-0.761821	-1.962312
H	3.793987	-0.847131	-2.565580
H	3.771029	2.008066	-3.516941
H	2.372124	1.299650	-2.673328
H	3.050137	2.846702	-2.133528
H	-3.438644	-1.424825	3.552520
H	-2.972580	-2.931721	5.478812
H	-0.635071	-3.242138	6.285552
H	1.228133	-2.031920	5.149896
H	0.741458	-0.544191	3.211837
H	-4.139575	-1.338548	-3.139497
H	-5.612158	-3.224979	-3.813579
H	-6.933654	-4.455544	-2.093534
H	-6.792393	-3.755900	0.295922
H	-5.339370	-1.843540	0.950565
H	1.149193	-2.790417	-3.036366
H	-0.516341	-2.681101	-3.594404
H	1.021719	-1.588572	-5.235453
H	-0.060160	-0.363748	-4.522384
H	1.625112	-0.487195	-3.974968
H	-3.257397	-3.811362	-0.844102
H	-1.502579	-3.667488	-0.994407
H	-2.200426	-4.902193	0.066634
H	-1.397089	-3.031675	1.495395
H	-3.144898	-3.047944	1.551937
C	0.333192	-2.077427	-3.233634
C	-0.641861	-1.052581	-0.932612
C	-0.097364	-1.472629	-1.976644
C	-2.295380	-2.850468	0.876962
C	-2.316635	-3.860609	-0.278516
C	0.754463	-1.066533	-4.308203

110

TS$_{syn/anti}$

C	-0.989217	0.709956	3.531984
C	-1.230179	1.433497	2.355626
C	-0.378127	2.503908	2.036173

C	0.683739	2.838832	2.872589
C	0.920762	2.108161	4.040440
C	0.081770	1.041104	4.363552
N	-2.277228	1.054991	1.481025
C	-3.277355	1.977061	1.217290
C	-4.146392	1.661540	0.161102
C	-5.330912	2.291493	-0.166158
C	-5.635257	3.445297	0.575162
C	-4.757141	3.866910	1.580854
C	-3.599941	3.157447	1.917665
C	-6.177726	1.664157	-1.284675
C	-6.074428	0.138415	-1.128876
C	-4.848158	-0.378822	-0.758817
O	-3.775957	0.491551	-0.520677
C	-7.106660	-0.798319	-1.302328
C	-6.848000	-2.154099	-1.070911
C	-5.604142	-2.622091	-0.634403
C	-4.547983	-1.710539	-0.434982
N	-3.294418	-1.928999	0.122083
AL	-2.123899	-0.421051	0.260442
C	-7.630812	2.139206	-1.225417
C	-5.567218	2.066229	-2.651522
C	-2.931038	-3.255032	0.457396
C	-2.824680	-4.254926	-0.523728
C	-2.400113	-5.537684	-0.184465
C	-2.067771	-5.845836	1.138068
C	-2.181455	-4.861326	2.120621
C	-2.622702	-3.580584	1.785983
AU	2.225204	0.216542	-0.929708
P	4.423978	0.034901	-0.200327
C	5.494865	1.327515	-1.127418
C	5.666090	0.871656	-2.586221
C	4.514385	0.354709	1.687293
C	4.307864	1.856617	1.944391
C	5.062168	-1.732509	-0.590209
C	4.425834	-2.721805	0.400726
C	3.319899	-0.351973	2.356126
C	5.822181	-0.095395	2.350966
C	4.541471	-2.139001	-1.983703
C	6.587971	-1.889924	-0.544074
C	4.710813	2.653420	-1.191361
C	6.877870	1.576919	-0.511352
H	-2.969755	3.489762	2.741713
H	-6.547365	4.006153	0.380068
H	-8.103474	-0.478479	-1.600092
H	-5.463550	-3.678592	-0.411639
H	-8.213527	1.690534	-2.039850
H	-7.682143	3.228139	-1.350668
H	-8.102729	1.874348	-0.270436
H	-6.136904	1.602072	-3.468183

H	-4.522026	1.741675	-2.729523
H	-5.601910	3.157856	-2.770280
H	-4.997189	4.769698	2.144692
H	-7.654620	-2.875504	-1.209636
H	4.808084	-3.193524	-2.151401
H	3.447867	-2.048494	-2.036293
H	4.972110	-1.552315	-2.798510
H	4.661135	-3.739825	0.056147
H	4.817413	-2.621306	1.417925
H	3.333284	-2.623681	0.425034
H	6.832012	-2.948400	-0.722538
H	7.092986	-1.305541	-1.320960
H	7.011264	-1.612364	0.427660
H	4.174135	1.996647	3.027325
H	5.163607	2.465823	1.635839
H	3.399570	2.226852	1.451480
H	3.322526	-0.087923	3.424140
H	2.369100	-0.005244	1.933188
H	3.354620	-1.440732	2.279085
H	5.786556	0.190820	3.413463
H	5.957439	-1.181932	2.312940
H	6.705608	0.379056	1.909890
H	7.422469	2.284184	-1.155679
H	6.814510	2.028251	0.484885
H	7.478958	0.663890	-0.439852
H	6.123219	1.700247	-3.147451
H	6.324674	0.003485	-2.689029
H	4.698448	0.644918	-3.053486
H	5.271092	3.350365	-1.832839
H	3.718329	2.498722	-1.635924
H	4.577105	3.129380	-0.217312
H	-1.655484	-0.115376	3.786588
H	0.256001	0.464555	5.273179
H	1.755906	2.367981	4.691720
H	1.339795	3.668556	2.604905
H	-0.552642	3.054331	1.111252
H	-3.057538	-4.005027	-1.559244
H	-2.313286	-6.298963	-0.961049
H	-1.726281	-6.847736	1.399618
H	-1.935481	-5.092781	3.157829
H	-2.735592	-2.814639	2.554702
H	0.703286	0.852993	-3.692465
H	-0.964874	1.213586	-3.223509
H	0.324938	3.328043	-3.557342
H	-0.160166	3.166126	-1.849471
H	1.511560	2.815901	-2.326682
H	0.439499	-0.480105	1.189423
H	-0.830192	-1.540135	1.806777
H	1.059256	-2.974764	1.013803
H	-0.334461	-3.225565	-0.049551

H	1.010478	-2.192364	-0.578484
C	0.278234	0.418662	-1.625927
C	0.081436	1.270264	-2.886102
C	0.462160	2.732394	-2.643888
C	-0.795627	-0.023647	-1.044382
C	-0.270086	-1.255806	0.887994
C	0.393994	-2.477283	0.287851
